# Effects of Platycodins Folium on Depression in Mice Based on a UPLC-Q/TOF-MS Serum Assay and Hippocampus Metabolomics

**DOI:** 10.3390/molecules24091712

**Published:** 2019-05-02

**Authors:** Cuizhu Wang, Hongqiang Lin, Na Yang, Han Wang, Yan Zhao, Pingya Li, Jinping Liu, Fang Wang

**Affiliations:** 1 Department of Pathogen Biology, College of Basic Medical Sciences, Jilin University, Changchun 130021, China; wangcz15@mails.jlu.edu.cn; 2 School of Pharmaceutical Sciences, Jilin University, Fujin Road 1266, Changchun 130021, China; linhq17@mails.jlu.edu.cn (H.L.); yangn0227@163.com (N.Y.); hanw17@mails.jlu.edu.cn (H.W.); lipy@jlu.edu.cn (P.L.); 3 College of Chinese Medicinal Materials, Jilin Agriculture University, Xincheng Street 2888, Changchun 130118, China; zhaoyan@jlau.edu.cn

**Keywords:** Platycodins Folium, LPS-induced depression, metabolomics, UPLC-Q/TOF-MS

## Abstract

Major depressive disorder (MDD), also known as depression, is a state characterized by low mood and aversion to activity. Platycodins Folium (PF) is the dried leaf of *Platycodon grandiflorum*, with anti-inflammatory and antioxidative activities. Our previous research suggested that PF was rich in flavonoids, phenols, organic acids, triterpenoid saponins, coumarins and terpenoids. This study aimed to investigate the antidepressant effect of PF using lipopolysaccharide (LPS)-induced depressive mice. Several behavior tests (sucrose preference test (SPT), forced swimming test (FST) and tail suspension test (TST)) and biochemical parameters (IL-6, TNF-α and SOD levels) were used to evaluate the antidepressive effect of PF on LPS-induced depression model. Furthermore, a UPLC-Q/TOF-MS-based metabolomics approach was applied to explore the latent mechanism of PF in attenuating depression. As a result, a total of 21 and 11 metabolites that potentially contribute to MDD progress and PF treatment were identified in serum and hippocampus, respectively. The analysis of metabolic pathways revealed that lipid metabolism, amino acid metabolism, energy metabolism, arachidonic acid metabolism, glutathione metabolism and inositol phosphate metabolism were disturbed in a model of mice undergoing MDD and PF treatment. These results help us to understand the pathogenesis of depression in depth, and to discover targets for clinical diagnosis and treatment. They also provide the possibility of developing PF into an anti-depressantive agent.

## 1. Introduction

Major depressive disorder (MDD), also known simply as depression, is a state of low mood and aversion to activity that represents a very serious global health problem. It is usually accompanied by a loss of appetite, low self-esteem, loss of interest in generally delightful activities, weight loss, fatigue, insomnia and low energy [1,2]. MDD interferes with the daily routine of patients and imposes a heavy burden on patients, their family and society [3]. However, the cause of depression remains unclear due to the complexity of its pathogenesis [4]. Due to the limited proper understanding of MDD, the available treatments using synthetic chemical antidepressants are generally accompanied by high toxicity and some side effects, for instance, hypertensive crisis, hepatotoxicity and somnolence commonly occur [5,6]. Compared to synthetic antidepressants, traditional Chinese medicine plays a crucial role in the prevention and treatment of diseases, because it has fewer side effects and improved safety [7]. *Platycodon grandiflorum* (Jacq.) A. DC. is a traditional Chinese medicine, which has been extensively used for the treatment of excessive phlegm, cough, and sore throat [8]. Platycodins Folium (PF) is the dried leaf of *Platycodon grandiflorum.* PF displayed obvious anti-inflammatory effects in four kinds of animal models of acute experimental inflammation, and increased dose-dependent DPPH free radical scavenging activity [9,10]. PF also exerts anti-tumor effects by improving the immune system and inducing cell apoptosis in H22 tumor-bearing mice [11]. In addition, PF is rich in chemical components, such as essential amino acids, especially glutamic acid and arginine, dietary fiber and minerals, polyphenols, triterpenoid saponins, phenolic acids, polyacetylenes, sterols, and flavonoids [10,12]. Our previous studies showed that components such as flavonoids, phenols, organic acids, triterpenoid saponins, coumarins and terpenoids could be identified in PF [13]. 

Metabolomics has been proposed as a powerful tool to explore the potential mechanism of medicines containing a multi-biochemical component [14,15,16]. Various analytical techniques have been widely applied in metabolomics studies, such as nuclear magnetic resonance (NMR), gas chromatography mass spectrometry (GC-MS) and ultra-high-performance liquid chromatography combined with quadrupole time-of-flight mass spectrometry (UPLC-Q/TOF-MS). Especially, the latter technique combined with multivariate statistics have made it easier to screen and identify the functional metabolites [17]. Most importantly, the main advantage of this method is that it provides an overall overview of multiple biochemical pathways that could underlie a pathophysiological state. Therefore, it is beneficial for the study of multifunctional disorders, such as depression. In this study, the metabolomics strategy was used to investigate the anti-depressive effect of PF in LPS-stimulated mouse models and demonstrate the potential biomarkers and relevant metabolic pathways.

## 2. Results

### 2.1. Effects of PF on Behavioral Evaluations 

#### 2.1.1. Effects of PF on SPT and BW

**Figure 1:**
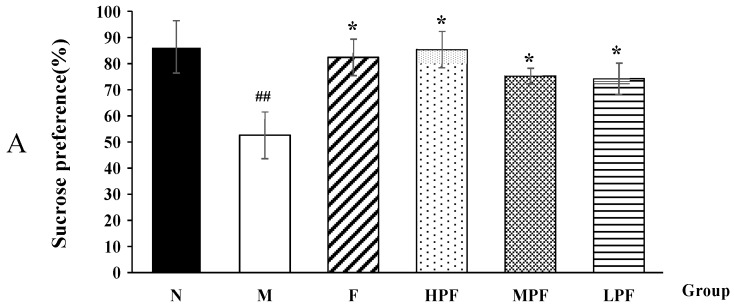

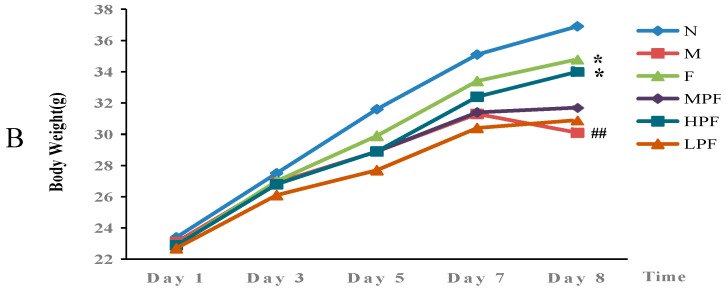
Effects of PF on the SPT (**A**) and BW (**B**) in mice. (compared with the normal control group, ^##^ *p* < 0.01; compared with the LPS-induced model group, * *p* < 0.05).

After LPS stimulation, the model group consumed less sucrose in SPT and showed a significant decrease in body weight, compared with the normal control group (Figure 1). This indicated that the model was well established and the mice exhibited depression-related symptoms of anhedonia and loss of appetite. However, compared with the model group, the interventions of fluoxetine and PF significantly increased sucrose preference and body weight. The HPF group showed a similar effect on the F group. 

#### 2.1.2. Effects of PF on the Immobility Time in FST

**Figure 2:**
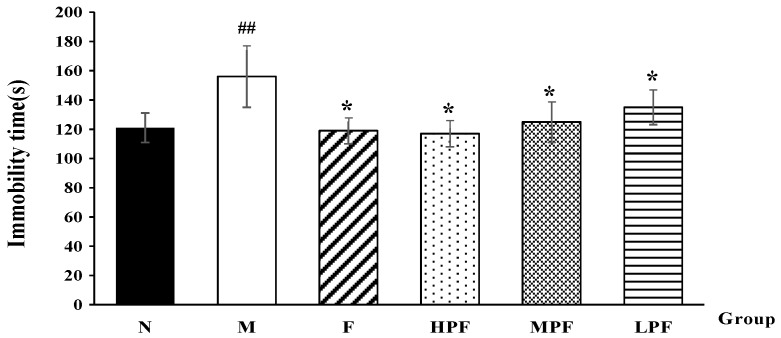
Effects of PF on the immobility time in FST (compared with the normal control group, ^##^ *p* < 0.01; compared with the LPS-induced model group, * *p* < 0.05).

The immobility time in FST was widely used to evaluate the depressive state of animals. Compared with the normal control group, the LPS-induced model group showed a significantly longer immobility time, indicating that LPS could exert a depressive-like effect in mice. Nevertheless, fluoxetine and PF treatment remarkably decreased the immobility time, compared with the model group, as shown in Figure 2. In addition, the immobility times of the HPF and MPF groups were much shorter than that of the LPF group.

#### 2.1.3. Effects of PF on the Immobility Time in TST

**Figure 3:**
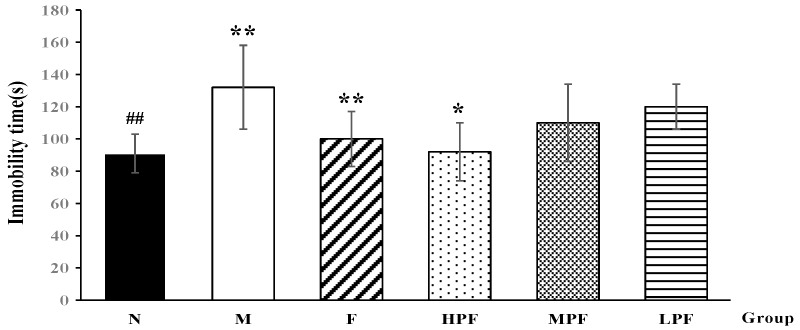
Effects of PF on the immobility time in FST (compared with the normal control group, ^##^ *p* < 0.01; compared with the LPS-induced model group, * *p* < 0.05, ** *p* < 0.01).

The immobility time in the TST could also reflect the state of depression in mice. Consistent with previous reports [18], LPS-induced depressive mice showed an obvious increase in the immobility time in TST in this study, which suggested behavioral despair in these mice. However, as a result of interventions, the immobility time of the F and HPF groups became much shorter than that of the model group (*p* < 0.05), as shown in Figure 3. 

### 2.2. Effects of PF on Biochemical Parameters

**Figure 4:**
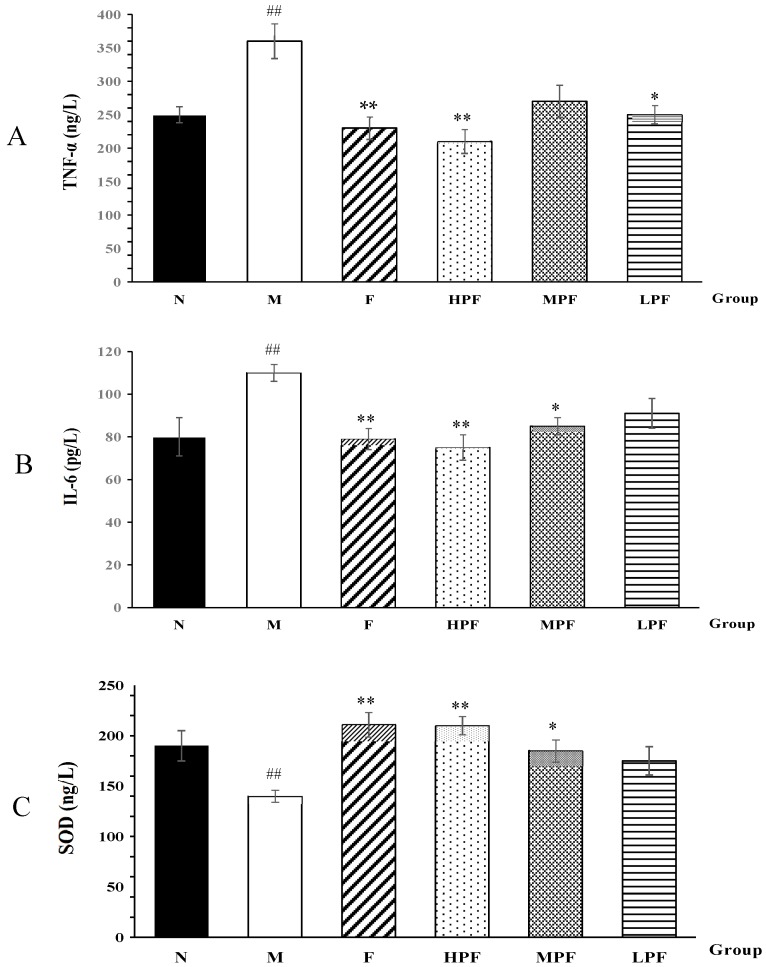
Effects of PF on TNF-*α* (**A**), IL-6 (**B**) and SOD (**C**) activity (compared with normal control group, ^##^ *p* < 0.01; compared with the LPS-induced model group, * *p* < 0.05, ** *p* < 0.01).

Inflammation is always regarded as one of the potential pathophysiological causes of depression [18]. After the mice underwent LPS stimulation, it was observed that the concentrations of IL-6 and TNF-α were significantly elevated, compared with the normal control group (Figure 4). In contrast to the M group, the serum TNF-α and IL-6 levels were significantly reduced after F, HPF and MPF treatment. There was no significant difference in the levels of IL-6 and TNF-α between the fluoxetine and HPF groups. In addition, in the hippocampus tissue, the SOD activity in the M group was significantly decreased, compared to the N group. Furthermore, the levels in the F and HPF and MPF groups were significantly increased, compared to the M group.

### 2.3. Metabolic Profiles of LPS-induced Depression under HPF Treatment

#### 2.3.1. Validation of UPLC-Q/TOF-MS

The metabolic profiles of the serum and hippocampus samples were obtained using UPLC-Q/TOF-MS in both the positive and negative mode. Before analyzing the experimental samples, the applied method must be validated. In order to monitor the stability of the system, the QC sample was run every eight samples in the analysis. We selected eight ions from different spectral regions: *m*/*z* 203.0451, 0.72 min; *m*/*z* 771.2939, 4.44 min; *m*/*z* 274.2619, 12.77 min; *m*/*z* 415.2084, 14.35 min; *m*/*z* 520.3399, 16.93 min; *m*/*z* 496.3402, 17.98 min; *m*/*z* 524.3692, 20.01 min; *m*/*z* 758.5780, 28.19 min in positive ion mode and *m*/*z* 268.7998, 0.59 min; *m*/*z* 809.2533, 4.51 min; *m*/*z* 824.2493, 5.77 min; *m*/*z* 514.2839, 9.68 min; *m*/*z* 564.3312, 16.57 min; *m*/*z* 540.3318, 17.98 min; *m*/*z* 568.3619, 20.59 min; and *m*/*z* 802.5616, 28.22 min in the negative ion mode, which covers the entire analysis process. Then, for the above ions in the QC sample, the relative standard deviations (RSDs) of the retention times and area intensities were calculated.

The injection precision was estimated by detecting five replicates of the QC sample in succession. For the serum samples, the RSDs of the area intensity ranged from 0.66% to 2.56%, and the RSDs of the retention time ranged from 0.01% to 0.22% in ESI+, while in the ESI−, the RSDs were from 0.17% to 2.76% for the area intensity and from 0.03% to 0.20% for the retention time. For the hippocampus samples, the RSDs of the area intensity and the retention time were 0.24–1.38% and 0.03−0.23% in ESI+, and 0.28–2.19% and 0.01–0.43% in ESI−, respectively.

The reproducibility of the sample preparation was assessed by analyzing five successive injections of a serum and a hippocampus sample, respectively. For the serum samples, the RSDs of the retention time were 0.19–0.67%, and those of the area intensities were 1.81–3.52% in ESI+, while they were 0.12–0.59% and 0.92–2.76% in ESI−. Besides, for the hippocampus samples, the RSDs of the retention times ranged from 0.10% to 3.98% in ESI+ and from 0.09% to 1.24% in ESI−, and the RSDs of the area intensities were 0.71–4.13% in ESI+ and 0.21–1.97% in ESI−.

The stability of the post-preparation of the samples was evaluated by detecting one sample settled in the auto-sampler for 0, 4, 8, 10, and 12 h at 4 °C [19]. The RSDs of the retention time and the area intensity were 0.12–0.21% and 1.21–4.99% in ESI+ and 0.09–0.31% and 0.95–5.42% in ESI−, respectively. 

It can be seen, from the above, that the injection precision, reproducibility of the sample preparation and stability of the post-preparation were good in the UPLC-Q/TOF-MS method. Thus, this method could be used for analyzing a large number of samples.

#### 2.3.2. Identification of the Differential Metabolites and Metabolic Pathways

**Figure 5:**
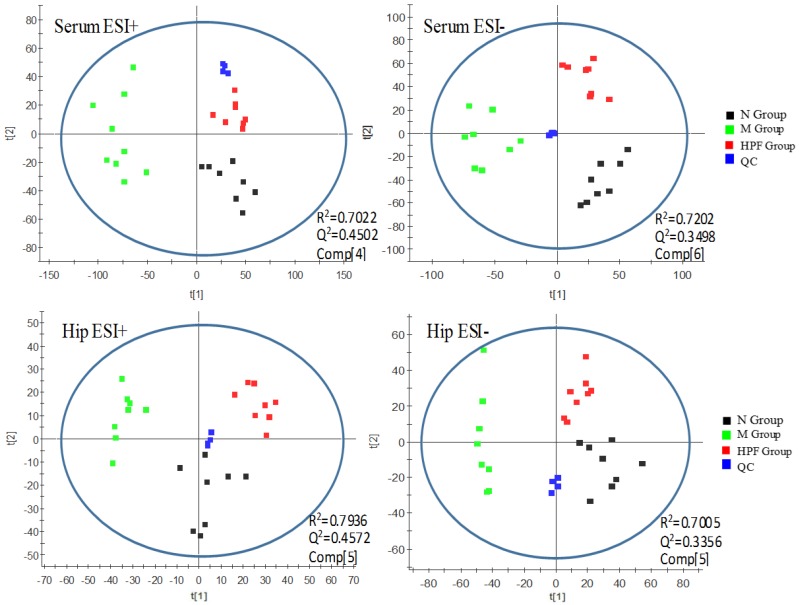
PCA score plots of serum and hippocampus metabolic profiling of the N, M, HPF groups.

Principal component analysis (PCA) is an unsupervised pattern recognition approach and could be applied to select distinct variables and look for possible biomarkers. As shown in Figure 5, a clear metabolites separation of the normal control group (N), LPS-induced model group (M) and high-dose PF group (HPF) could be observed through the PCA analysis in both the serum and hippocampus samples in ESI+ and ESI−. The HPF group was located between the M group and N group tending toward the N group, which revealed that there was an alteration in the specific biomarkers in the model, and LPS-induced metabolic disturbances were significantly regulated by PF treatment. R^2^ (model’s goodness of fit) was 0.7022 (serum, ESI+), 0.7202 (serum, ESI−), 0.7936 (hippocampus, ESI+) and 0.7005 (hippocampus, ESI−). Q^2^ (goodness of prediction) was 0.4502 (serum, ESI+), 0.3498 (serum, ESI−), 0.4572(hippocampus, ESI+) and 0.3356 (hippocampus, ESI−), respectively. 

**Figure 6:**
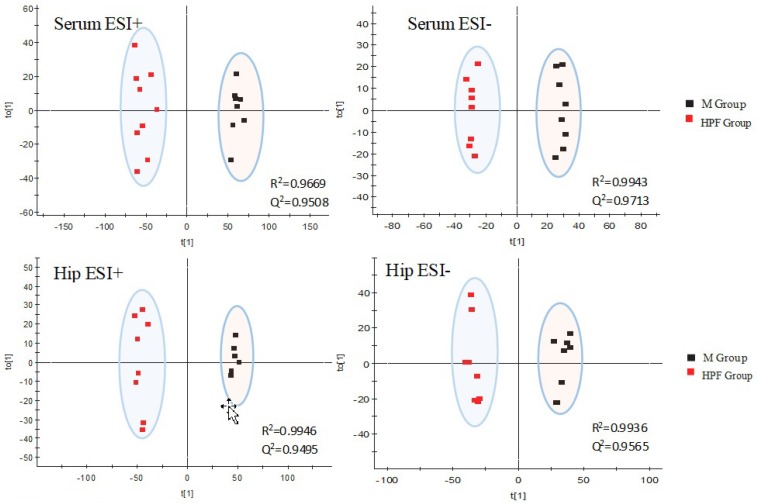
OPLS-DA score plots of serum and hippocampus metabolic profiling of the M and HPF group.

Orthogonal Projections to Latent Structures Discriminant Analysis (OPLS-DA), a supervised method of pattern recognition, could allow the general metabolic variation between the M and HPF groups to be visualized and depicted. As shown in Figure 6, the M and HPF groups were separated with a satisfactory goodness of fit (R^2^ = 0.9669, Q^2^ = 0.9508 in ESI+, R^2^ = 0.9943, Q^2^ = 0.9713 in ESI− in serum; R^2^ = 0.9946, Q^2^ = 0.9495 in ESI+, R^2^ = 0.9936, Q^2^ = 0.9565 in ESI− in hippocampus).

**Figure 7:**
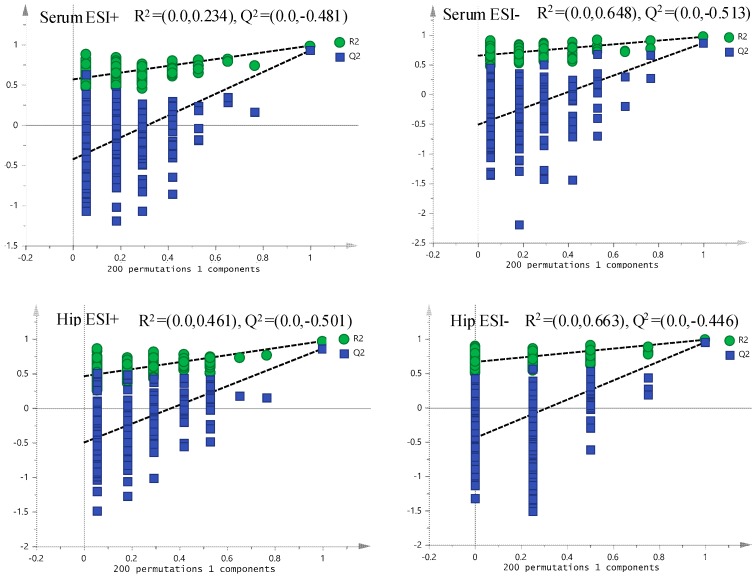
The permutations plots of the OPLS-DA models of serum and the hippocampus.

**Figure 8:**
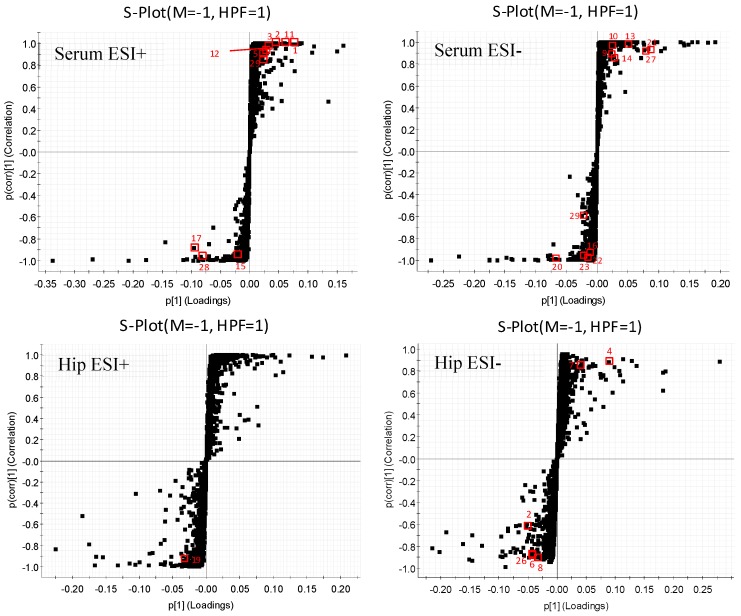
OPLS-DA *S*-plots of serum and hippocampus metabolic profiling. (Numbers marked are consistent with the No. of each biomarker in Table 1).

**Figure 9:**
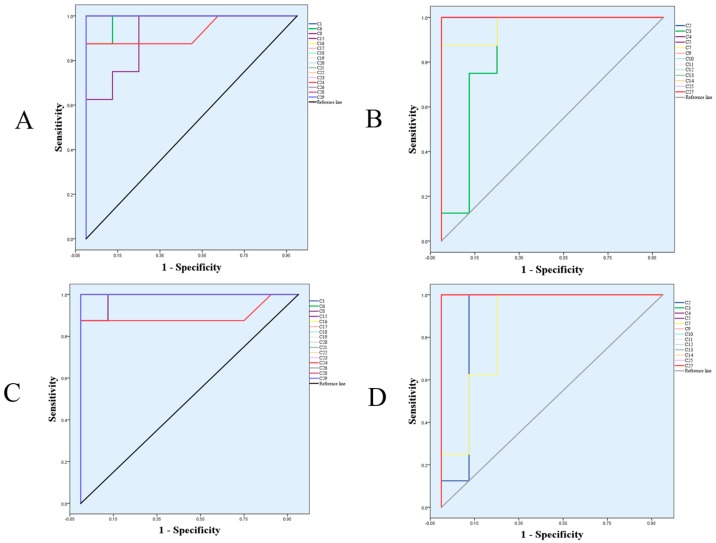
The predictive ROC curves generated using 29 biomarkers contributing to MDD progress and PF treatment. (**A**) C_M_ > C_N_; (**B**) C_M_ < C_N_; (**C**) C_M_ > C_HPF_; (**D**) C_M_ < C_HPF_. The numbers are consistent with the No. in Table 1.

Each spot in the OPLS-DA score plot is representative of a sample. From the permutations plots, all blue Q^2^-values to the left were lower than the original points to the right, suggesting the validity of the original models (Figure 7). The *S*-plots were generated to identify the potential metabolites (Figure 8). *S*-plot is a tool for visualizing the covariance and correlation between the metabolites and the modeled class. The *S*-plots were generated to identify the potential metabolites statistically and biochemically under the OPLS-DA model, which could contribute to the screen of the differential variables between groups. In the *S*-plots, each spot is representative of a compound. The farther away the spots in the *S*-plots from the origin, the more significantly they contribute to the clustering of the M and HPF groups. Only if the variable importance in the projection (VIP) value was above 1.0, and the *p*-value was below 0.05, could the metabolites be considered as potential biomarkers. A total of 29 endogenous metabolites were chosen as potential biomarkers in both the serum and hippocampus samples (Table 1). Pareto scaling was used to establish the PCA, OPLS-DA and *S*-plot. The predictive ROC curves were generated using the 29 metabolites identified (the biomarkers between the N and M group & M and HPF group). The ROC analysis between the M and N groups showed that most of them were potential diagnostic markers for MDD except *α*-ketoglutarate with *p* > 0.01 (Figure 9, Table 2). Another predictive ROC curve was generated between the M and HPF groups (Figure 9, Table 2), indicating that most of the metabolites contributed to PF treatment except pantetheine 4′-phosphate with *p* > 0.01. A correlation plot between potential biomarkers observed in the serum and hippocampus, has been generated based on O2PLS. O2PLS is a generalization of OPLS. It is bidirectional, i.e. X (22 potential biomarkers observed in serum) and ↔Y (11 potential biomarkers observed in hippocampus). 

**Figure 10:**
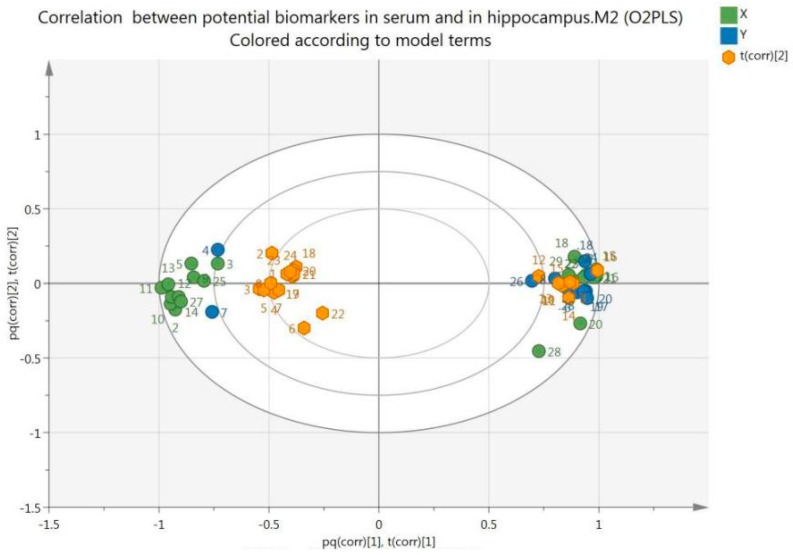
Bi-plot of biomarkers observed in serum (S, green spots) and hippocampus (H, blue spots).

Based on the O2PLS, a bi-plot was generated (Figure 10), which showed the correlation between the potential biomarkers observed in serum and the hippocampus. It displays a bi-plot that simultaneously shows the relationship among scores and loadings. The scores and loadings are expressed using correlation scaling. Observations situated near variables are high in these variables and are low in variables situated opposite. 

**Figure 11:**
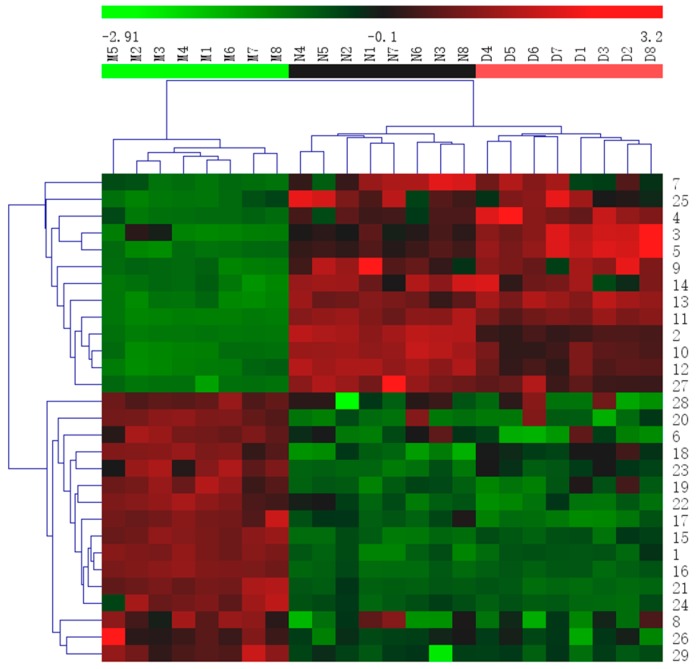
Heatmap of all potential biomarkers. The color indicates the relative abundance of the biomarkers. Green, lowest; red, highest. Rows indicate potential biomarkers, and columns indicate samples. The numbers marked are consistent with the No. of each biomarker in Table 1.

Then, a heat map was generated from the biomarkers to effectively visualize and depict the distinction between groups (Figure 11).

**Figure 12:**
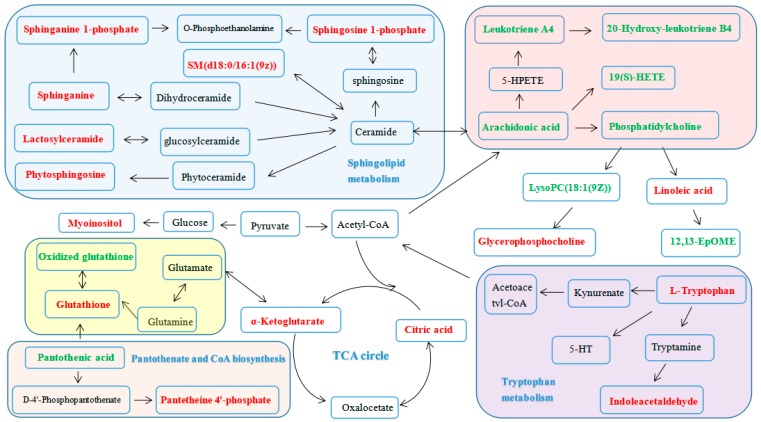
The metabolic pathways of serum and the hippocampus. (Red labeled metabolites: potential biomarkers with increased levels in the HPF group, compared to the M group. Green labeled metabolites: potential biomarkers with decreased levels in the HPF group, compared to those of the M group).

The metabolic network of these biomarkers was established (Figure 12), which clearly showed that PF could regulate the alterations in glycerophospholipid metabolism (GlyM), tryptophan metabolism (TM), sphingolipid metabolism (SphM), arachidonic acid metabolism (AM), linoleic acid metabolism (LM), glutathione metabolism (GluM), TCA cycle (TCA), pantothenate and CoA biosynthesis (PCB) and inositol phosphate metabolism (IM) (Table 3).

## 3. Discussion

The LPS-induced depressive model is widely used to evaluate the activities of anti-depressants. The animals’ behavior [18,20], inflammation [21,22,23,24] and oxidative stress [25] are all the important factors in the evaluation of an antidepressant-like effect. Body weight, sucrose preference, mobility time in the forced swim test (FST) and tail suspension test (TST), as well as the levels of IL-6, TNF-α and SOD, are common indexes used [18].

The antidepressant-like effects of PF on the LPS-induced depression model were investigated for the first time. The results demonstrated that PF intervention could significantly increase sucrose preference and body weight, decrease immobility time, reduce pro-inflammatory cytokines TNF-*α* and IL-6 levels, and increase the SOD level. The behavioral and biochemical results showed that the behavioral modulation effects of PF were accompanied by the PF-induced modulation of IL-6 and TNF-α levels in serum and SOD level in hippocampus. It was also demonstrated that the anti-depressive effect of PF was associated with anti-inflammation and anti-oxidation activities. 

The metabolomics study based on UPLC-Q/TOF-MS combined with multivariate statistical analysis further illustrated the anti-depressive effect of PF. Firstly, the clear separation of the normal control group, LPS-induced model group and HPF group (located between the normal and model groups, tending toward the normal group), observed in PCA analysis revealed that LPS-induced metabolic disturbances were significantly regulated by PF treatment. Secondly, twenty-nine potential biomarkers were identified in our study, which clearly showed that PF could regulate the alterations in glycerophospholipid metabolism, tryptophan metabolism, sphingolipid metabolism, arachidonic acid metabolism, linoleic acid metabolism, glutathione metabolism, the TCA cycle, pantothenate and CoA biosynthesis and inositol phosphate metabolism. These metabolisms were related to depression in the following ways.

### 3.1. Lipid Metabolism 

It was found that sphingolipid metabolism (SphM), glycerophospholipid metabolism (GlyM) and linoleic acid metabolism (LinM) were all involved in the therapeutic effect of PF. Firstly, the model group had decreased serum levels of phytosphingosine (**11**), sphinganine (**12**), sphingosine 1-phosphate (**13**), sphinganine 1-phosphate (**14**), lactosylceramide (**25**) and SM (**27**), which indicated that depression could cause the perturbation of SphM. This is in accordance with previous research [26,27,28]. Both **13** and **14** are important intermediates in SphM. SM (**27**) could induce synaptic abnormalities associated with neurological disorders [28,29]. These sphingolipids are highly enriched in the central nervous system and are very important for various brain functions. The up-regulation of them indicated an anti-depressive effect of PF. Secondly, the model group had decreased levels of PCs (**19**, **23**, **24**, **26**, **28**, **29**) and LysoPC (**18**) and an increased level of glycerophosphocholine (**1**), which suggesting that the metabolic abnormality of GlyM played an important role in LPS-induced depression. Glycerophospholipids, including PCs and LysoPC, were reported to be related to multiple neurodegenerative diseases including depression [30,31]. Generally, PCs are the most abundant phospholipids in membranes and are expected to alter mitochondrial lipid profiles and consequently impact the development of depression [30,32]. The regulation of them also indicated an anti-depressive effect of PF. Thirdly, PCs are also important metabolites in LinM, which played an important role in depression [33,34]. Previous studies have suggested that linoleic acid (**21**) and its derivative 12,13-Epome (**16**) could affect the occurrence and development of depression, which is consistent with our results. The results in our study suggested that there are important connections between lipid metabolism, the pathogenesis of depression and the anti-depressive effect of PF. 

### 3.2. Amino Acid Metabolism 

Amino acids play vital roles in the life activities of an organism. Tryptophan could be transported into the brain by blood [34]. As the precursor of 5-hydroxytryptamine (5-HT), tryptophan plays an important role in the development of depression [1,35,36]. Our results revealed that the tryptophan (**9**) and indoleacetaldehyde (**10**) levels in serum were markedly decreased in the model group, suggesting an imbalanced tryptophan metabolism and lower conversion ratio of tryptophan to 5-HT, which may therefore affect the 5-HT content in the mice. However, PF was observed to prevent the reduction tendency of these two metabolites, suggesting the therapeutic effects of PF.

### 3.3. Energy Metabolism

Energy deficiency plays an important role in the development of depression [37]. The TCA cycle could release stored energy via the oxidation of acetyl-CoA into chemical energy and carbon dioxide by all aerobic organisms [38]. CoA has been regarded as an indispensable cofactor of biochemical reactions in lots of organisms for long time [39]. Pantothenic acid, the precursor of coenzyme A (CoA), may contribute to the depressive process by increasing the CoA level, inhibiting inflammation and reducing oxidative stress [40]. Moreover, 2-oxoglutarate [41], citric acid and α-ketoglutarate [42] were also reported to be related to depression. In the current study, the reduction of α-ketoglutarate (**3**), citric acid (**5**) and pantetheine 4′-phosphate (**7**) and the increase of pantothenic acid (**8**) were observed after LPS-stimulation alone, which could be regulated by PF treatment.

### 3.4. Other Metabolisms 

Inflammation was reported to be related to the onset of various diseases such as cancer and depression [43,44]. Arachidonic acid metabolism, plays a vital role in the progression of inflammatory responses [45] and is associated with depression. When the body was stimulated, arachidonic acid was hydrolyzed and released, which could be generated into a variety of active substances. Among them, leukotriene A4 could be generated from arachidonic acid by cyclooxygenase and further converted into leukotriene B4 by leukotriene A4 hydrolase. In this study, elevated levels of leukotriene A4 (**15**), 19(*S*)-Hete (**17**), arachidonic acid (**20**), 20-hydroxyleukotriene B4 (**22**), and PCs **19**, **22**, **23**, **24**, **26**, **28**, **29** were observed in the model group, which implied that imbalance of arachinodic acid metabolism could be a symptom of depression. Meanwhile, reduced levels of the above metabolites were found in the PF treatment group, indicating that PF intervened in the depression response and inhibited the perturbed metabolism. 

Glutathione (GSH) could prevent some thiol-containing proteins or enzymes from damage by some oxidants such as peroxide [46]. It was shown that GSH may be a potential marker of major depressive disorder in the early stages of illness [47]. We observed a decreased GSH (**4**) and increased oxidized GSH (**6**) in the model group, which was consistent with the tendency of the SOD levels, indicating that oxidative stress is an important factor associated with the pathogenesis of depression, and PF had the ability to regulate the antioxidant capacity of organisms. 

Myoinositol is identified as a glia-specific marker, and previous studies reported that the resonance of myoinositol in the prefrontal cortex and hippocampus of depressed patients was reduced [48]. Adding myoinositol to the diet could relieve depression [49]. In the present study, low level of myoinositol (**2**) was detected in the serum of depressive mice, which was up-regulated by PF.

In a word, the regulation of the above metabolisms could indicate the anti-depressive effect of PF. 

## 4. Materials and Methods

### 4.1. Chemicals

PF was collected from Jilin Province, Northeastern China and identified by Professor Ping-Ya Li (School of Pharmaceutical Sciences, Jilin University). PF was air-dried, grinded and sieved (40 mesh) to obtain a homogenous powder. Then the powder was extracted thrice with 70% methanol at 80 °C (3 h each time). After being filtered, the extracted solution was combined, concentrated and evaporated to obtain the PF extract. The chemical components of PF were elucidated in a previous study [13].

Acetonitrile and methanol were of UPLC-MS grade and purchased from Fisher Chemical Company (Geel, Belgium). Formic acid suitable for UPLC was from the Sigma-Aldrich Company (St. Louis, MO, USA). Deionized water was purchased from the A.S. Watson Group Ltd (Hong Kong, China). Other chemicals were all of analytical grade. LPS was bought from Sigma-Aldrich (*Escherichia coli* O127: B8, batch number: 20171102). Four standard compounds, including glycerophosphocholine (110512-201703), pantothenic acid (111765-201709), 12,13-EpOME (112450-201702), and lactosylceramide (110831-201745), were purchased from Shanghai Zhen Zhun Biological Technology Co., Ltd. (Shanghai, China). Two standard compounds, including phytosphingosine (101302-201704) and sphinganine (111853-201709) were purchased from Beijing Century Aoke Biological Technology Co., Ltd. (Beijing, China). Five standard compounds, including sphingosine 1-phosphate (100120-201710), arachidonic acid (10931), myoinositol (1340960), glutathione (1294820) and linoleic acid (100218-201603), were purchased from Sigma-Aldrich. 20-Hydroxyleukotriene B4 (111201-201608) was provided by Shanghai Yifei Biotechnology Co., Ltd. (Shanghai, China). Oxidized glutathione (101302-201701) was purchased from Shanghai Yiji Industrial Co., Ltd. (Shanghai, China). 19 (*S*)-HETE (111802-201606) was purchased from Xi’an Ruixi Biological Technology Co.,Ltd. (Xi’an, China). Additionally, *α*-ketoglutarate (102001-201707) was purchased from Beijing Fubo Biotechnology Co., Ltd. (Beijing, China). Citric acid (111679-201602) and L-tryptophan (140686-201303) were bought from the National Institutes for Food and Drug Control (Beijing, China). The IL-6 ELISA Kit and TNF-α ELISA Kit were bought from R&D Systems, Ltd. (Minneapolis, MN, USA), and the superoxide dismutase (SOD) ELISA Kit was purchased from the Nanjing Jiancheng Bioengineering Institute (Nanjing, China).

### 4.2. Animals and Administration

Animal experiments were conducted in accordance with the protocols approved by the Review Committee of Animal Care and Use of Jilin University. This study was carried out according to the ethical principles for animal use and care. Adult male ICR mice, weighing 20 ± 2 g, were purchased from Changchun Yisi Experimental Animal Technology Co., Ltd (Changchun, China). All of the mice had free access to standard laboratory food and water and were maintained under standardized laboratory conditions (temperature 21–23 °C, relative humidity 40–60%, 12 h light/dark cycle, with the light cycle beginning at 7:00 a.m.) for 7 days, before the experiment.

After one week of acclimatization, the mice were randomly divided into six groups (with 16 in each group): normal control group (N), LPS-induced model group (M), LPS + Fluoxetine (20 mg·kg^−1^·day) group (F), LPS + PF (100 mg·kg^−1^·day) group (LPF), LPS + PF (200 mg·kg^−1^·day) group (MPF), LPS + PF (400 mg·kg^−1^·day) group (HPF). N group and M group were administered a normal saline solution (0.9% NaCl aqueous). The F group was administered fluoxetine. The LPS + PF groups were administered PF extract. The doses were determined based on the unpublished acute toxicity test and dose screening experiment. All the mice were administered their respective solutions once daily (i.g. 10 mL/kg) for 7 consecutive days. On the 7th day, all mice received a single LPS (0.83 mg/kg, i.p.) or vehicle (0.9% NaCl aqueous) at 0.5 h after the last drug treatment [50]. LPS was dissolved in isotonic saline solution that was sterile and endotoxin-free. After fasting for 8 h, behavioral evaluation, including body weight (BW), sucrose preference test (SPT), tail suspension test (TST) and forced swimming test (FST) was performed on the 8th day (LPS stimulation after 24 h). During the test, the mice had free access to water. The mice were permitted to rest for 1h between the TST and FST. 

Thereafter, 1 mL of blood was collected from the retroorbital plexus immediately after the FST, then clotted at 4 °C for 1 h and centrifuged at 3500 *g* for 15 min at 4 °C to obtain the serum. The brains were rapidly isolated on ice after the blood collection, followed by immediately taking the hippocampus samples. Then the serum and hippocampus were quickly frozen in liquid nitrogen and then stored at −80 °C. In order to investigate the anti-depressive effect of PF, the serum and hippocampal tissues from all of the groups were prepared for pharmacological assess. While aiming at finding the potential biomarkers among model group, most effective dose group and normal group, the serum and hippocampal tissue in N, M and HPF group were also used for metabolomics assess.

### 4.3. Behavioral Tests

#### 4.3.1. Sucrose Preference Test (SPT) and Body Weight (BW)

SPT was performed to assess whether the mouse depression model was successfully established as previously described [51]. In order to ensure the stability of the baseline consumption, the test procedure was conducted to reduce the reaction to novelty of the mice [52]. On the testing day, the mice water-deprived for 12 h were exposed to two pre-weighed bottles of 1% sucrose and water. After 12 h, the two bottles were taken away and weighed to calculate the consumption of water and sucrose. The sucrose preference was the percentage of sucrose solution consumed, relative to the total liquid consumption. The formula was as follows: sucrose preference = (sucrose intake/[sucrose consumption + water consumption]) × 100%. Moreover, body weight is also an important index for evaluating the depressive state of mice, because the depressive mice usually lose appetite and weight, which was measured before and 24 h after the LPS treatment.

#### 4.3.2. Forced Swimming Test (FST)

The FST was performed, according to the procedure described previously, in order to assess the behavioral despair of the mice [53]. The mice were forced to swim for 6 min individually in an open cylinder-shaped flask (height 25 cm, diameter 10 cm) with 15 cm of fresh water at 23–25 °C. The duration of immobility was assayed during the last 4 min of the test. 

#### 4.3.3. Tail Suspension Test (TST)

The procedure of TST was the same as described in a previous study [18]. Mice were suspended by taping the tail at 1 cm from the tip and 75 cm above the floor. Mice were suspended for 6 min and considered immovable if they did not have an escape-oriented behavior. The duration of immobility was measured during the last 4 min of the test.

### 4.4. ELISA Determination

The serum levels of IL-6 and TNF-*α* as well as the hippocampus levels of SOD were assessed using a commercial ELISA kit according to the manufacturer’s instructions. 

### 4.5. Spectrum Acquisition

#### 4.5.1. Sample Collection and Preparation

Serum: Before analysis, the serum and hippocampus samples were thawed at room temperature. Methanol (900 µL) was added to the serum (300 µL) and vortex-mixed for 3 min [54,55]. Then, the mixture was settled at room temperature for 10 min and centrifuged at 10,000 rpm for 10 min at 4 °C. After that, the supernatant was blow-dried with a gentle stream of nitrogen at 37 °C. After being dissolved in 500 µL of methanol/water (4:1) and centrifuged at 10,000 rpm for 10 min at 4 °C, the serum was obtained.

Hippocampus: The hippocampus was washed with a 0.9% saline solution and homogenized with methanol/water (4:1), then centrifuged at 10,000 rpm for 10 min at 4 °C. The following steps were the same in the procedure for the serum.

The serum and hippocampus samples were filtrated through 0.22 µm before UPLC-Q/TOF-MS analysis. Meanwhile, 20-μL aliquot of each serum and hippocampus sample was mixed to acquire a quality control (QC) sample for method validation. As a consequence, the QC sample contained nearly all of the metabolites in this analysis.

#### 4.5.2. UPLC-Q/TOF-MS Conditions

UPLC-Q/TOF-MS analysis was performed on a Waters Xevo G2-XS QTOF mass spectrometer (Waters Co., Milford, MA, USA), equipped with a UPLC system by an electrospray ionization (ESI) interface. Chromatographic separation was performed on an ACQUITY UPLC BEH C18 (100 mm × 2.1 mm, 1.7 μm) from the Waters Co. The mobile phases were composed of eluent A (0.1% formic acid in water, *v*/*v*) and eluent B (0.1% formic acid in acetonitrile, *v*/*v*), and the flow rate was set at 0.4 mL/min. The elution conditions applied were: 0–2 min, 10% B; 2–26 min, 10–90% B; 26–28 min, 90% B; and 28–28.1 min, 90–10% B. The temperature of the UPLC column was set at 30 °C and the temperature of the sample was 15 °C. Mixtures of 90/10 and 10/90 water/acetonitrile were used as the weak wash solvent and the strong wash solvent respectively. The positive mode conditions were: capillary voltage, 2.6 kV; cone voltage, 40 V; source temperature, 150 °C; desolvation temperature, 400 °C; cone gas flow, 50 L/h; desolvation gas flow, 800 L/h. The mass spectrum was obtained from 100 to 1200 Da in the MS^E^ mode. There was only one condition that was different from the positive mode compared with the negative mode: the capillary voltage was 2.2 kV. The ramp collision energy of the high energy function was 20–40 V, while that of the low energy function was 6 V. During a single LC run, data acquisition was conducted through a mass spectrometer by quickly switching from a low-collision energy scan to a high-collision energy scan. Leucine encephalin with a constant flow of 10 μL/min was used as an external reference of the Lock Spray™. What is more, the MS data scanning format was an MS^E^ continuum.

### 4.6. Data Analysis

For behavioral and biochemical assessments, the results were presented as the mean ± standard deviation (SD). Tukey’s test was used in the statistical analysis and a result was accepted as statistically significant if the P value was less than 0.05.

For metabolomics analysis, the MarkerLynx XS V4.1 software, a post-acquisition processing package applied to MS data files, was used for quantitative analysis in metabolomics study (see Supplementary Materials). The software uses a combination of spectral deconvolution, peak integration, normalisation and sample alignment automatically [56]. It determines the abundance of each mass-retention pair in terms of area intensity across all samples and then subjects the abundance matrix to principal component analysis. It detects chromatographic peaks using ApexPeak Track, the peak width at a height of 5% and the peak-to-peak baseline noise will be automatically determined. The main collection parameters were as follows: marker intensity threshold: 2000 counts; mass window: 0.10 Da; and retention time window: 0.20. Moreover, the noise elimination level was set at 6× the std dev of the noise, and the data were deisotoped before processing to prevent the assignment of isotope peaks as markers. The other parameters are as follows: the mass range was 100–1200 Da, retention time range was 0–28 min. After processing the data in MarkerLynx, the results could be shown in the Extended Statistics (XS) Viewer. *m*/*z*-RT pairs with corresponding intensities for all the detected peaks from each datum were listed. The probable molecular formulas of the biomarkers were calculated by high-accuracy quasi-molecular ion, and the mass tolerance was within ±10 ppm. The MS/MS fragments of the matched components, which were acquired from 10 to 40 eV were compared with the fragments in Metlin. Potential biomarkers were eventually confirmed by comparing reference substances with retention time (tolerance was 0.1 min), *m*/*z* (tolerance was 0.1 Da) and MS fragmentation patterns. The same value of RT, *m*/*z* and similar fragments were regarded as belonging to the same component. Then, some biochemical databases, including HMDB (http://www.hmdb.ca/), METLIN (http://metlin.scripps.edu/), KEGG (http://www.kegg.com/) and Metabo-Analyst (http://www.metaboanalyst.ca/), were used to further identify potential biomarkers. Base on the data of MetaboAnalyst, the metabolic pathways with the impact-value threshold above 0.10 were selected as the most potential ones [21]. 

## 5. Conclusions

MDD is a psychiatric disorder that is involved in several metabolic pathways. Several behavioral tests (SPT, BW, FST and TST) and biochemical parameters (IL-6, TNF-α and SOD) were used to evaluate the antidepressive effects of PF on an LPS-induced depression model for the first time. The UPLC-Q/TOF-MS-based serum and hippocampus metabolomics methods were established and used to explore the mechanism. The anti-depressive effect of PF was closely associated with the regulation of some metabolic pathways, including lipid metabolism, amino acid metabolism, energy metabolism, arachidonic acid metabolism, glutathione metabolism and inositol phosphate metabolism. 

In the current study, metabolomics, combined with multivariate statistics analysis, was helpful in understanding the potential biomarkers involved in LPS-induced depression and the regulation of metabolic deviations of the biomarkers, after PF treatment. This is the first time that the anti-depressive effects of PF and its metabolomics have been investigated. Twenty-nine metabolites, with remarkable changes compared to the the model group, were regarded as potential biomarkers which could be regulated by PF treatment, suggesting that PF had a therapeutic effect on depression, because it regulated these metabolic pathways. In short, the discovered metabolites are potential targets for further laboratory investigations, and PF is a potential anti-depressive agent. 

## Figures and Tables

**Table 1:** Identification results of potential biomarkers in serum and the hippocampus.

No.	t_R_/min	Measured Mass (Da)	VIP	Formula	Mass Error (ppm)	Adducts	Biomarkers	HMDB ID	Pathway	Content Level	Source and Mode
1 *	0.58	280.0929	2.17	C_8_H_20_NO_6_P	1.07	M + Na	Glycerophosphocholine	HMDB00086	Gly M	C_M_ < C_D_	Serum-ESI+
2 *	0.60	203.0528	2.56	C_6_H_12_O_6_	0.03	M + Na	Myoinositol	HMDB00211	IM	C_M_ < C_D_	Serum-ESI+
3 *	0.67	169.0117	1.23	C_5_H_6_O_5_	2.37	M + Na	*α*-Ketoglutarate	HMDB00208	TCA cycle	C_M_ < C_D_	Serum-ESI+
4 *	0.72	306.0746	5.14	C_10_H_17_N_3_O_6_S	−4.57	M − H	Glutathione	HMDB00125	Glu M	C_M_ < C_D_	Hip-ESI−
5 *	0.74	215.017	1.15	C_6_H_8_O_7_	0.93	M + Na	Citric acid	HMDB00094	TCA cycle	C_M_ < C_D_	Serum-ESI+
6 *	0.78	611.144	2.45	C_20_H_32_N_6_O_12_S_2_	−0.16	M − H	Oxidized glutathione	HMDB03337	Glu M	C_M_ > C_D_	Hip-ESI−
7 ^a^	0.85	357.0904	2.26	C_11_H_23_N_2_O_7_PS	5.32	M − H	Pantetheine 4′-phosphate	HMDB01416	PCB	C_M_ < C_D_	Hip-ESI−
8 *	0.9	218.1015	2.03	C_9_H_17_NO_5_	−5.96	M − H	Pantothenic acid	HMDB62717	PCB	C_M_ > C_D_	Hip-ESI−
9 *	1.46	203.0816	1.33	C_11_H_12_N_2_O_2_	−2.46	M − H	L-Tryptophan	HMDB00929	TM	C_M_ < C_D_	Serum-ESI−
10 ^a^	5.16	204.0651	1.45	C_10_H_9_NO	−4.90	M + FA − H	Indoleacetaldehyde	HMDB01190	TM	C_M_ < C_D_	Serum-ESI−
11 *	13.00	318.3011	3.61	C_18_H_39_NO_3_	0.94	M + H	Phytosphingosine	HMDB04610	SM	C_M_ < C_D_	Serum-ESI+
12 *	15.33	302.3054	1.82	C_18_H_39_NO_2_	−1.65	M + H	Sphinganine	HMDB00269	SM	C_M_ < C_D_	Serum-ESI+
13 *	15.43	378.2399	2.21	C_18_H_38_NO_5_P	−2.64	M − H	Sphingosine 1-phosphate	HMDB00277	SM	C_M_ < C_D_	Serum-ESI−
14 ^a^	15.98	380.2556	1.4	C_18_H_40_NO_5_P	−2.63	M − H	Sphinganine 1-phosphate	HMDB01383	SM	C_M_ < C_D_	Serum-ESI−
15 ^a^	16.79	341.2102	1.23	C_20_H_30_O_3_	2.64	M + Na	Leukotriene A4	HMDB01337	AM	C_M_ > C_D_	Serum-ESI+
	16.79	317.212	5.94	C_20_H_30_O_3_	0.95	M − H	Leukotriene A4	HMDB01337	AM	C_M_ > C_D_	Serum-ESI−
16 *	17.18	295.2265	1.24	C_18_H_32_O_3_	−2.71	M − H	12,13-EpOME	HMDB04702	LM	C_M_ > C_D_	Serum-ESI−
17 *	18.14	319.2272	11.62	C_20_H_32_O_3_	−0.31	M − H	19(*S*)-HETE	HMDB11136	AM	C_M_ > C_D_	Serum-ESI−
			1.68								Hip-ESI−
	18.14	343.224	2.67	C_20_H_32_O_3_	−2.62	M + Na	19(*S*)-HETE	HMDB11136	AM	C_M_ > C_D_	Serum-ESI+
18 ^a^	18.55	522.3565	5.75	C_26_H_52_NO_7_P	0.96	M + H	LysoPC (18:1(9Z))	HMDB02815	Gly M	C_M_ > C_D_	Serum-ESI+
			8.05								Hip-ESI+
	18.49	566.3466	4.92	C_26_H_52_NO_7_P	1.41	M + FA − H	LysoPC(18:1(9Z))	HMDB02815	Gly M	C_M_ > C_D_	Serum-ESI−
			4.99								Hip-ESI−
19 ^a^	22.75	782.5717	1.61	C_44_H_80_NO_8_P	2.17	M + H	18:1/18:3 phosphatidylcholine	HMDB08107	AM, Gly M	C_M_ > C_D_	Hip-ESI+
20 *	22.81	303.2330	4.88	C_20_H_32_O_2_	1.98	M − H	Arachidonic acid	HMDB01043	AM	C_M_ > C_D_	Serum-ESI−
			2.87								Hip-ESI−
21 *	23.14	279.2321	4.18	C_18_H_32_O_2_	−1.07	M − H	Linoleic acid	HMDB00673	LM	C_M_ < C_D_	Serum-ESI−
22 *	23.26	397.2243	1.1	C_20_H_32_O_5_	4.28	M + FA − H	20-Hydroxy-leukotriene B_4_	HMDB01381	AM	C_M_ > C_D_	Serum-ESI−
23 ^a^	25.4	828.5760	1.49	C_44_H_82_NO_8_P	0.60	M + FA − H	18:1/18:2 phosphatidylcholine	HMDB08137	AM, LM, Gly M	C_M_ > C_D_	Serum-ESI−
24 ^a^	25.91	826.5605	2.38	C_44_H_80_NO_8_P	0.85	M + FA − H	18:3/18:1 phosphatidylcholine	HMDB08203	AM, Gly M	C_M_ > C_D_	Hip-ESI−
25 *	26.11	806.5628	1.39	C_42_H_79_NO_13_	−0.25	M + H	Lactosylceramide	HMDB04866	SM	C_M_ < C_D_	Serum-ESI+
26 ^a^	26.38	824.5463	2.20	C_44_H_78_NO_8_P	2.55	M + FA − H	18:2/18:3 phosphatidylcholine	HMDB08141	AM, Gly M	C_M_ > C_D_	Hip-ESI−
27 ^a^	26.93	747.5648	2.91	C_39_H_79_N_2_O_6_P	−0.54	M + FA − H	SM (d18:0/16:1)	HMDB13464	SM	C_M_ < C_D_	Serum-ESI−
28 ^a^	27.13	782.5672	3.34	C_42_H_82_NO_8_P	−0.51	M + Na	16:0/18:1 phosphatidylcholine	HMDB07972	AM, LM, Gly M	C_M_ > C_D_	Serum-ESI+
	27.20	804.577	1.79	C_42_H_82_NO_8_P	1.86	M + FA − H	16:0/18:1 phosphatidylcholine	HMDB07972	AM, LM, Gly M	C_M_ > C_D_	Hip-ESI−
29 ^a^	28.61	828.5736	1.53	C_44_H_82_NO_8_P	−2.29	M + FA − H	18:2/18:1 phosphatidylcholine	HMDB08105	AM, LM, Gly M	C_M_ > C_D_	Serum-ESI−

* Metabolites validated with standards. ^a^ Metabolites confirmed by MS/MS fragments. “D” represents drug intervention group (HPF group); “M” represents model group; “N” represents normal control group; “<” indicates that the content level of the potential biomarker was significantly lower than that of the other group (*p* < 0.05); “>” indicates that the content level was significantly higher than that of the other group (*p* < 0.05); “≈” indicates that the content level was regulated tending to the normal level.

**Table 2:** The AUCs and *p* values of the biomarkers in different predictive ROC curves.

Compound No.	M & N		M & HPF	
	AUC	*p*	AUC	*p*
**1**	1.000	0.001	1.000	0.001
**2**	1.000	0.001	0.891	0.009
**3**	0.859	0.016	1.000	0.001
**4**	1.000	0.001	1.000	0.001
**5**	1.000	0.001	1.000	0.001
**6**	0.984	0.001	0.984	0.001
**7**	0.969	0.002	0.859	0.016
**8**	0.922	0.005	0.984	0.001
**9**	1.000	0.001	1.000	0.001
**10**	1.000	0.001	1.000	0.001
**11**	1.000	0.001	1.000	0.001
**12**	1.000	0.001	1.000	0.001
**13**	1.000	0.001	1.000	0.001
**14**	1.000	0.001	1.000	0.001
**15**	1.000	0.001	1.000	0.001
**16**	1.000	0.001	1.000	0.001
**17**	1.000	0.001	1.000	0.001
**18**	1.000	0.001	1.000	0.001
**19**	1.000	0.001	1.000	0.001
**20**	1.000	0.001	1.000	0.001
**21**	1.000	0.001	1.000	0.001
**22**	1.000	0.001	1.000	0.001
**23**	1.000	0.001	1.000	0.001
**24**	0.930	0.004	0.898	0.007
**25**	1.000	0.001	1.000	0.001
**26**	1.000	0.001	1.000	0.001
**27**	1.000	0.001	1.000	0.001
**28**	1.000	0.001	1.000	0.001
**29**	1.000	0.001	1.000	0.001

“M” represents model group; “N” represents normal control group; “HPF group” represents high-dose PF treatment group; “AUC” represents area under curve.

**Table 3:** Metabolic pathways of differential metabolites.

Pathway Name	Match Status	*p*	−log (*p*)	Holm *p*	FDR	Impact
Sphingolipid metabolism	6/21	1.1904 × 10^−6^	13.6410	9.7613 × 10^−5^	9.7613 × 10^−5^	0.1955
Linoleic acid metabolism	3/6	1.1897 × 10^−4^	9.0367	0.0096	0.0045	1.0
Rachidonic acid metabolism	5/36	4.2879 × 10^−4^	7.7545	0.0339	0.0088	0.5012
Glycerophospholipid metabolism	3/30	0.0178	4.0294	1.0	0.2917	0.2065
TCA cycle	2/20	0.0539	2.9224	1.0	0.7353	0.1216
Glutathione metabolism	2/26	0.0858	2.4552	1.0	0.9413	0.3979
Tryptophan metabolism	2/40	0.1755	1.7402	1.0	1.0	0.1954
Pantothenate and CoA biosynthesis	2/15	0.0315	3.4571	1.0	0.5169	0.3265
Inositol phosphate metabolism	1/28	0.4195	0.8686	1.0	1.0	0.1116

## Supplementary Materials

The following are available online.
