# Supplementary material for: Effects of Platycodins Folium on Depression in Mice Based on a UPLC-Q/TOF-MS Serum Assay and Hippocampus Metabolomics
Source: Molecules. 2019 May 2;24(9):1712. doi: 10.3390/molecules24091712 (PMC6540008; doi:10.3390/molecules24091712)

Supplementary Materials I

Each BPI Chromatogram of Every Serum/Hippocampus Sample in

Control/Model/HPF Group in ESI^+/ESI^- Mode

Supplementary Materials II

MS Spectra of Each Potential Biomarker

in Function 1 (Low Energy) and Function 2 (High Energy)

Supplementary Materials I

Each BPI Chromatogram of Every Serum/Hippocampus Sample in Control/Model/HPF Group in ESI^+/ESI^- Mode

1. In ESI^+ mode, each BPI chromatogram of every serum sample in normal control group (ESI+/ serum/ normal control group).
2. In ESI^+ mode, each BPI chromatogram of every serum sample in model group (ESI+/ serum/ model group).
3. In ESI^+ mode, each BPI chromatogram of every serum sample in HPF group (ESI+/ serum/ HPF group).
4. In ESI^+ mode, each BPI chromatogram of every hippocampus sample in normal control group (ESI+/ hip/ normal control group).
5. In ESI^+ mode, each BPI chromatogram of every hippocampus sample in model group (ESI+/ hip/ model group).
6. In ESI^+ mode, each BPI chromatogram of every hippocampus sample in HPF group (ESI+/ hip/ HPF group).
7. In ESI^- mode, each BPI chromatogram of every serum sample in normal control group (ESI-/ serum/ normal control group).
8. In ESI^- mode, each BPI chromatogram of every serum sample in model group (ESI-/ serum/ model group).
9. In ESI^- mode, each BPI chromatogram of every serum sample in HPF group (ESI-/ serum/ HPF group).

10. In ESI^- mode, each BPI chromatogram of every hippocampus sample in normal control group (ESI-/ hip/ normal control group).

11. In ESI^- mode, each BPI chromatogram of every hippocampus sample in model group (ESI-/ hip/ model group).

12. In ESI^- mode, each BPI chromatogram of every hippocampus sample in HPF group (ESI-/ hip/ HPF group).

1. ESI+/ serum/ normal control group

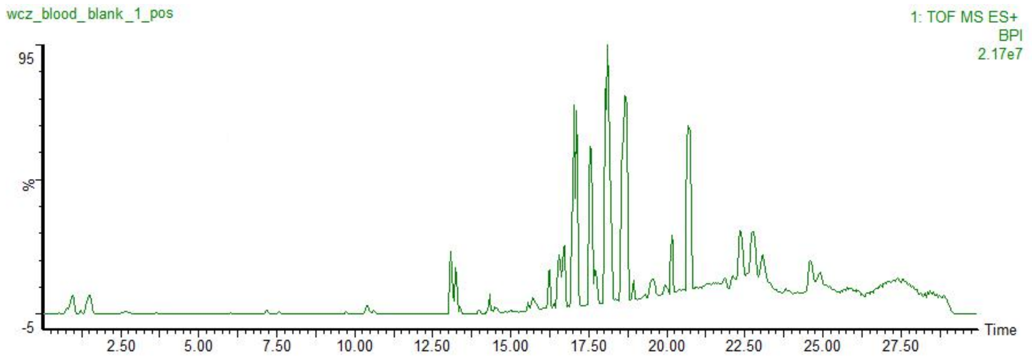

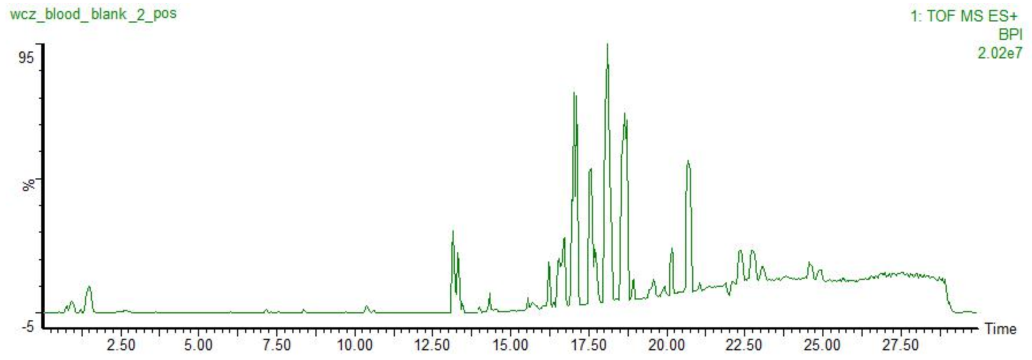

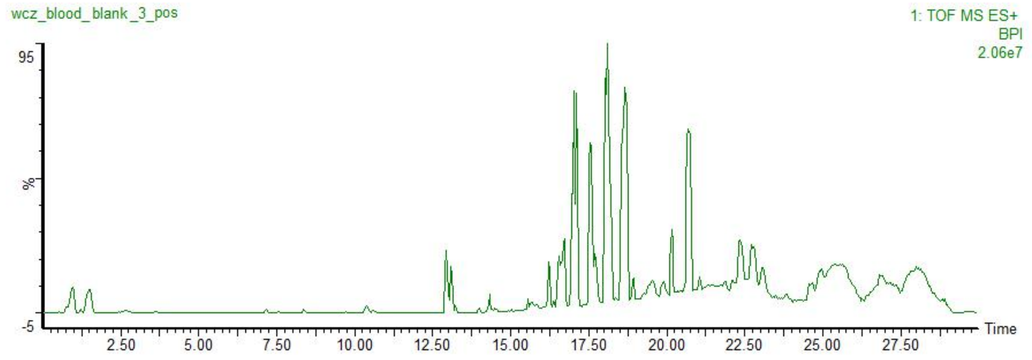

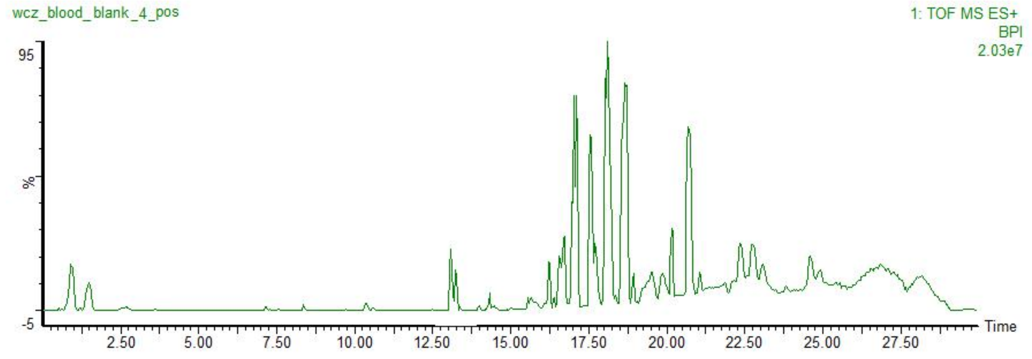

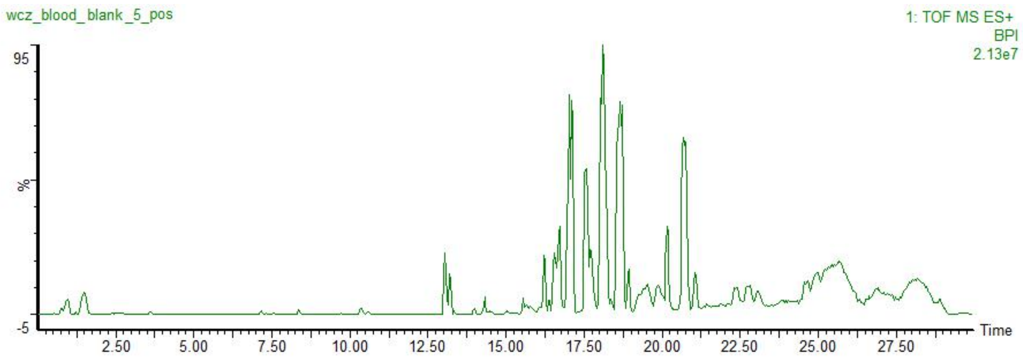
wcz_blood_blank_5_pos
1: TOF MS ES+
BPI
2.13e7
95
%
-5
2.50
5.00
7.50
10.00
12.50
15.00
17.50
20.00
22.50
25.00
27.50
Time

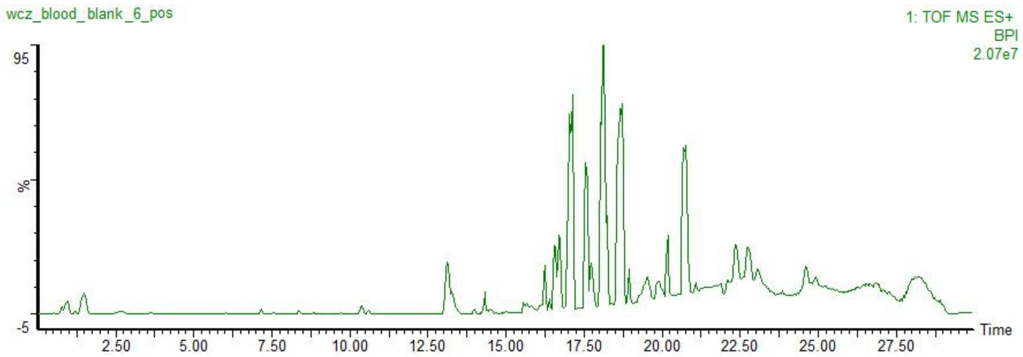
wcz_blood_blank_6_pos
1: TOF MS ES+
BPI
2.07e7
95
%
-5
2.50
5.00
7.50
10.00
12.50
15.00
17.50
20.00
22.50
25.00
27.50
Time

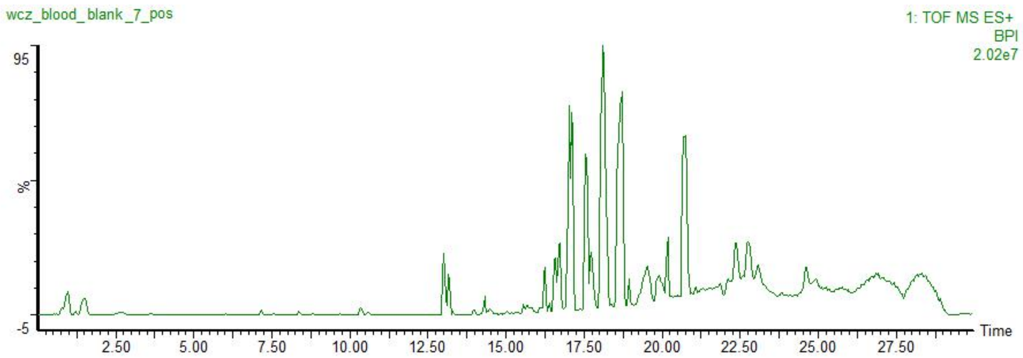
wcz_blood_blank_7_pos
1: TOF MS ES+
BPI
2.02e7
95
%
-5
2.50
5.00
7.50
10.00
12.50
15.00
17.50
20.00
22.50
25.00
27.50
Time

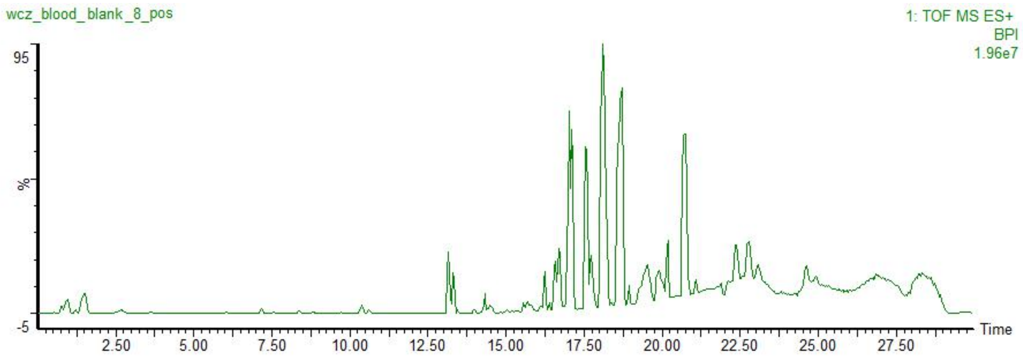
wcz_blood_blank_8_pos
1: TOF MS ES+
BPI
1.96e7
95
%
-5
2.50
5.00
7.50
10.00
12.50
15.00
17.50
20.00
22.50
25.00
27.50
Time

2. **ESI+/ serum/ model group**

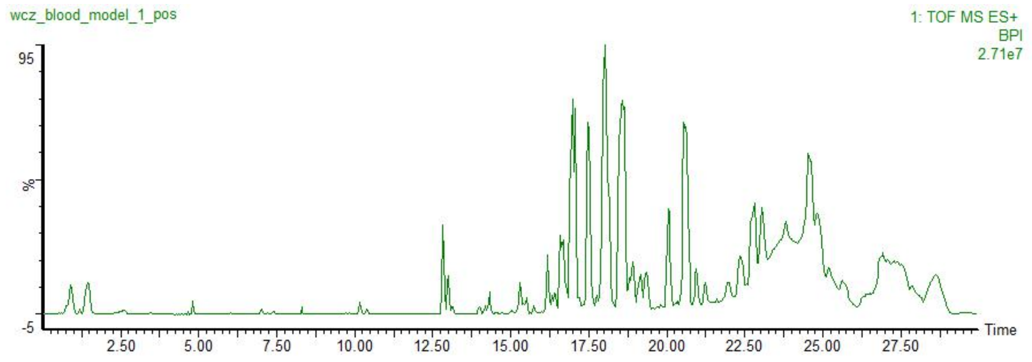

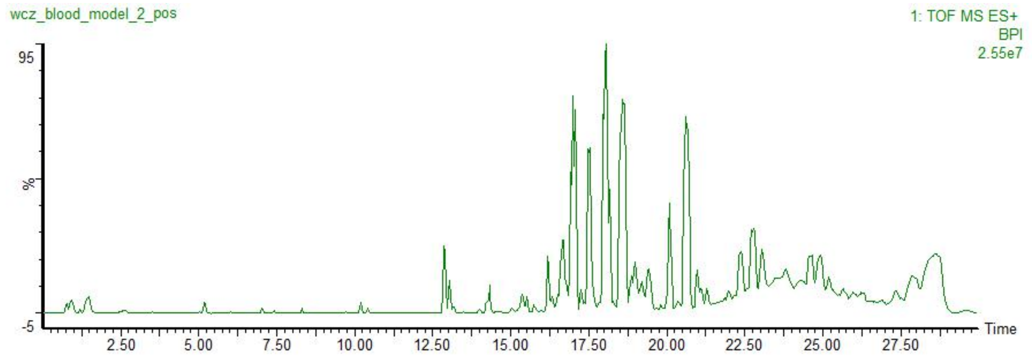

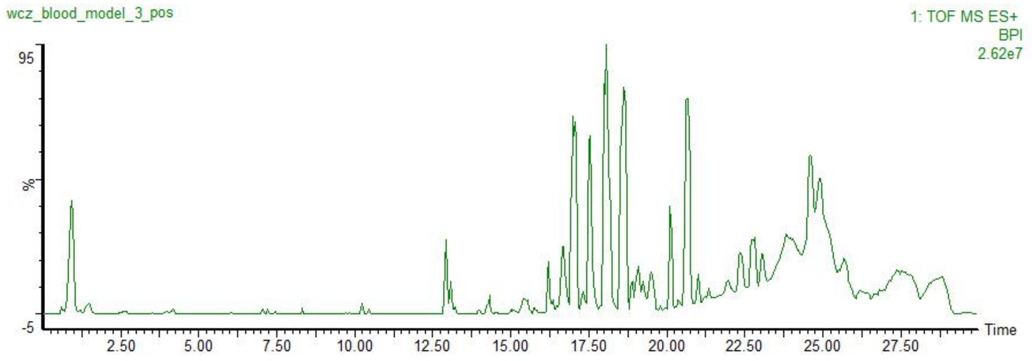

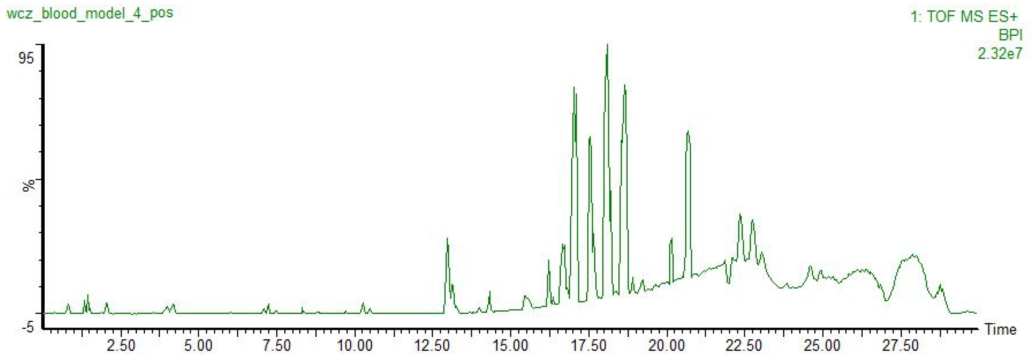

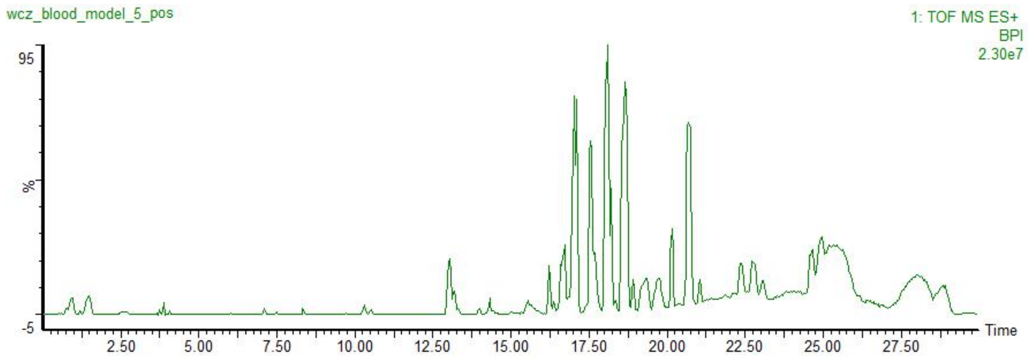
wcz_blood_model_5_pos
1: TOF MS ES+
BPI
2.30e7
95
%
-5
2.50
5.00
7.50
10.00
12.50
15.00
17.50
20.00
22.50
25.00
27.50
Time

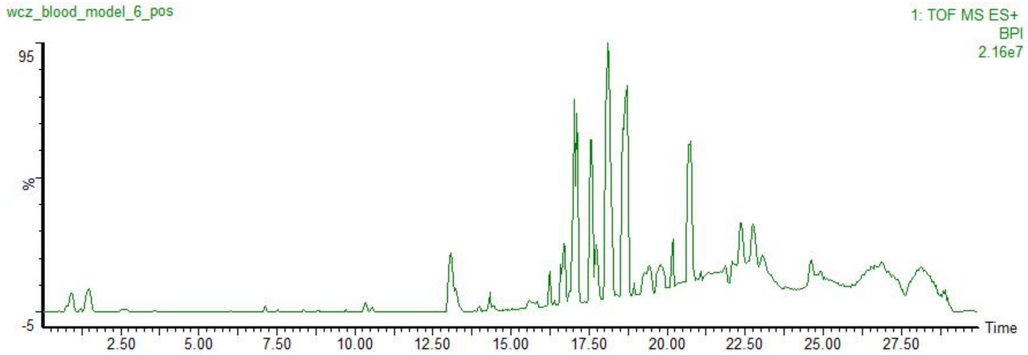
wcz_blood_model_6_pos
1: TOF MS ES+
BPI
2.16e7
95
%
-5
2.50
5.00
7.50
10.00
12.50
15.00
17.50
20.00
22.50
25.00
27.50
Time

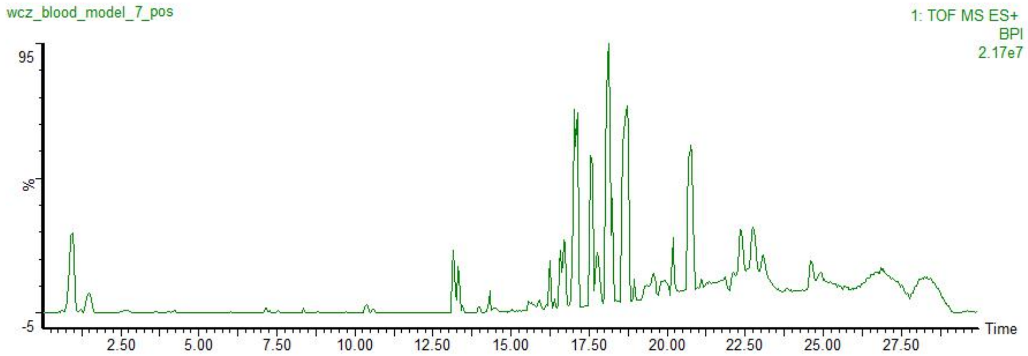
wcz_blood_model_7_pos
1: TOF MS ES+
BPI
2.17e7
95
%
-5
2.50
5.00
7.50
10.00
12.50
15.00
17.50
20.00
22.50
25.00
27.50
Time

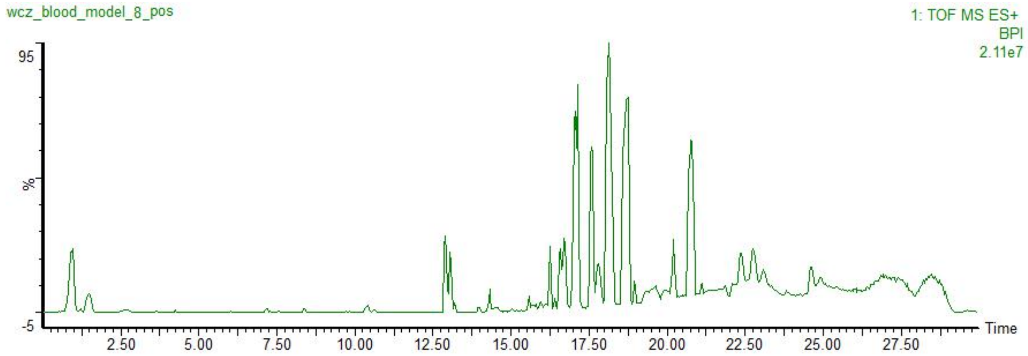
wcz_blood_model_8_pos
1: TOF MS ES+
BPI
2.11e7
95
%
-5
2.50
5.00
7.50
10.00
12.50
15.00
17.50
20.00
22.50
25.00
27.50
Time

3. **ESI+/ serum/ HPF group**

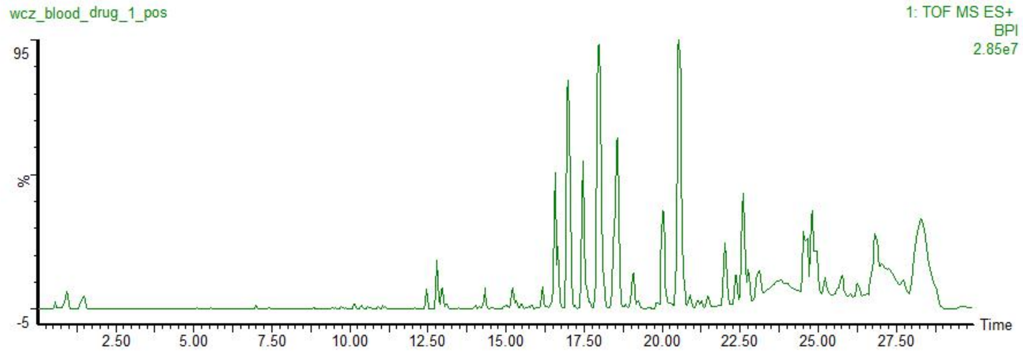

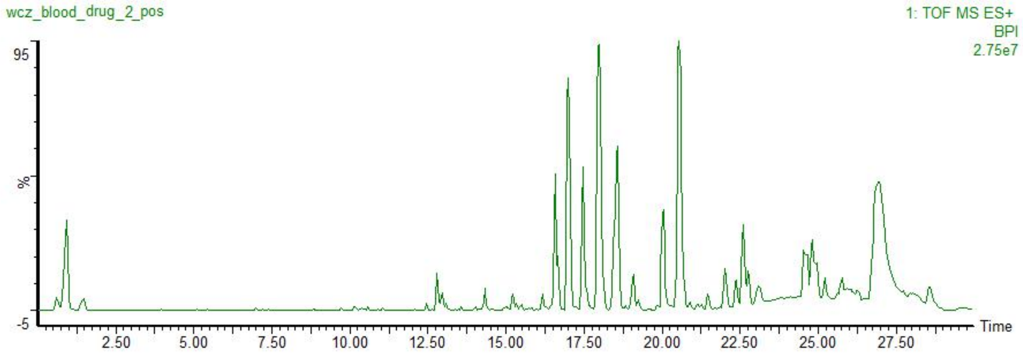

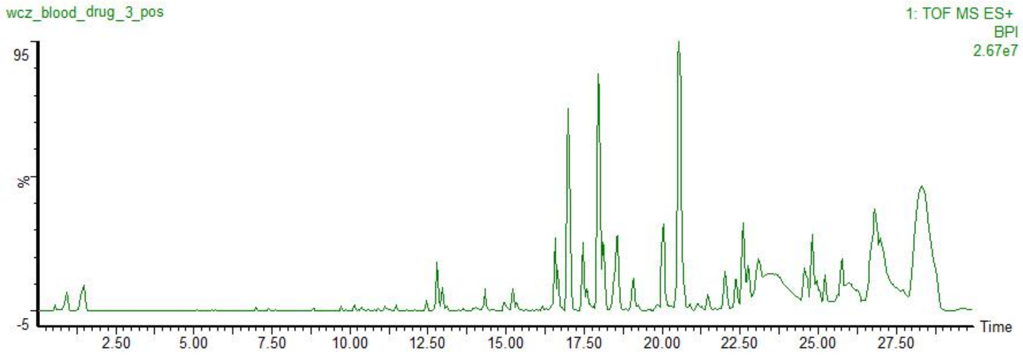

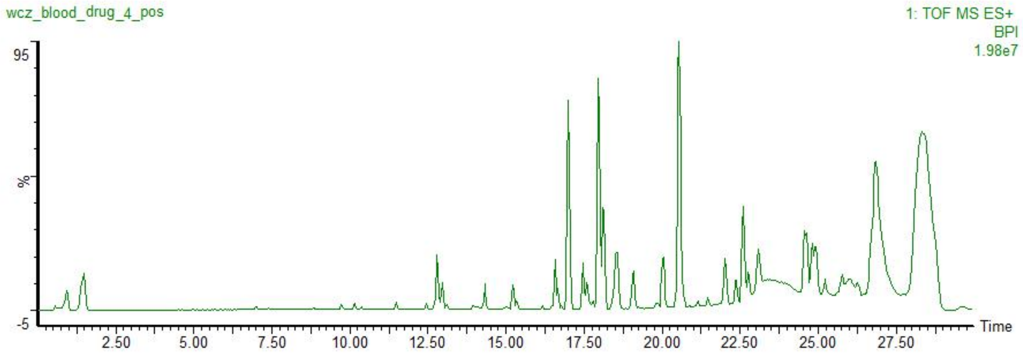

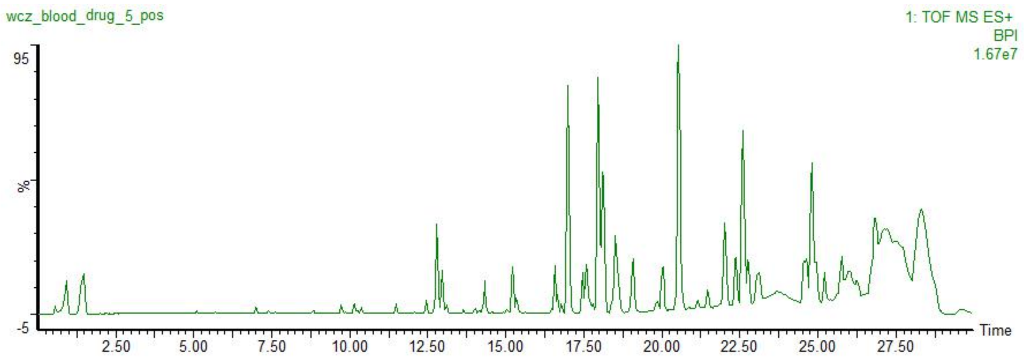
wcz_blood_drug_5_pos
1: TOF MS ES+
BPI
1.67e7
95
%
-5
2.50
5.00
7.50
10.00
12.50
15.00
17.50
20.00
22.50
25.00
27.50
Time

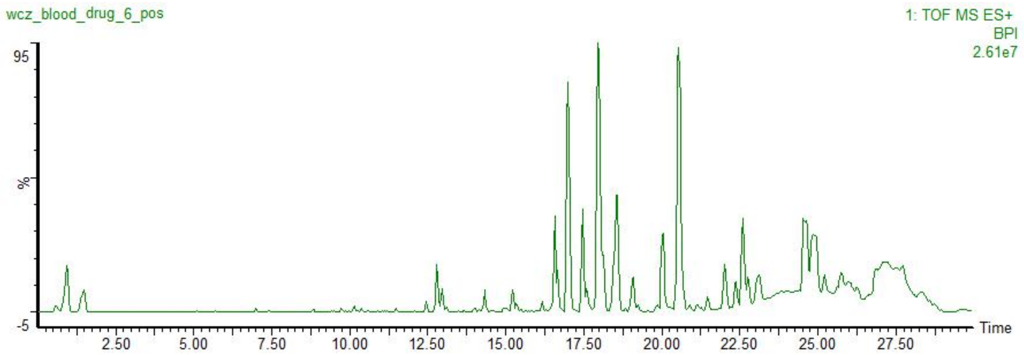
wcz_blood_drug_6_pos
1: TOF MS ES+
BPI
2.61e7
95
%
-5
2.50
5.00
7.50
10.00
12.50
15.00
17.50
20.00
22.50
25.00
27.50
Time

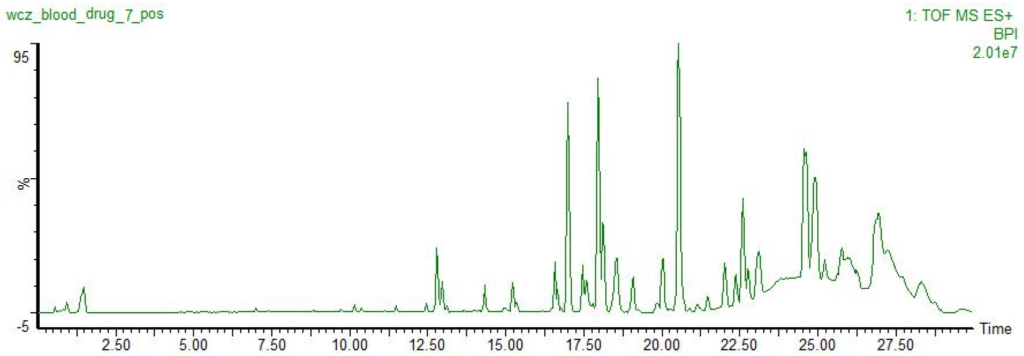
wcz_blood_drug_7_pos
1: TOF MS ES+
BPI
2.01e7
95
%
-5
2.50
5.00
7.50
10.00
12.50
15.00
17.50
20.00
22.50
25.00
27.50
Time

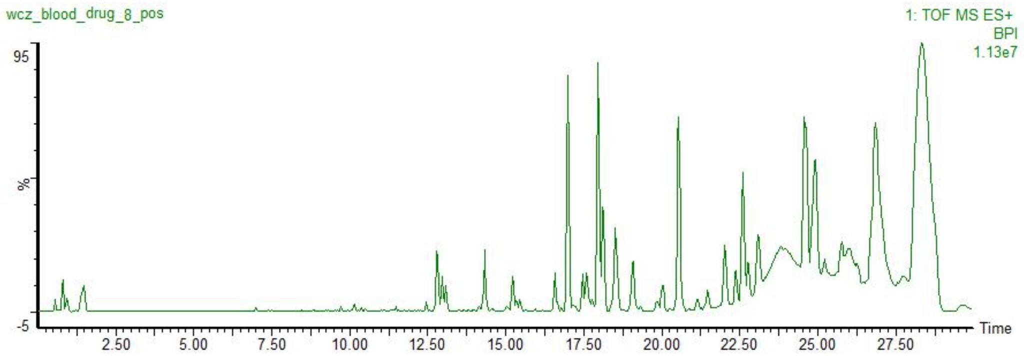
wcz_blood_drug_8_pos
1: TOF MS ES+
BPI
1.13e7
95
%
-5
2.50
5.00
7.50
10.00
12.50
15.00
17.50
20.00
22.50
25.00
27.50
Time

4. ESI+/ hip/ normal control group

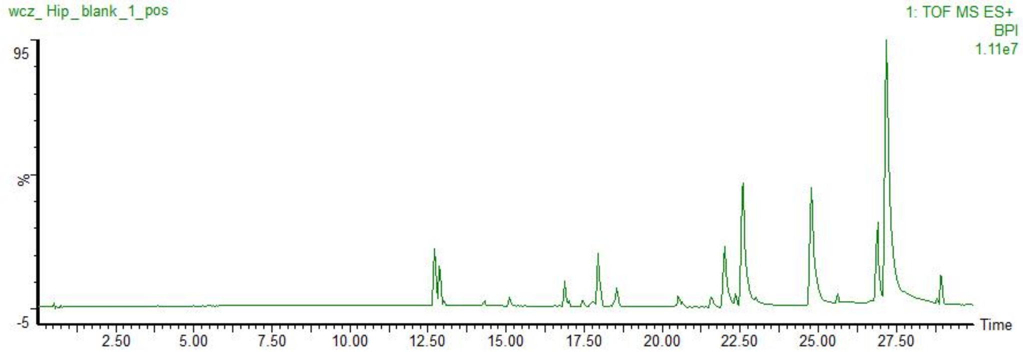

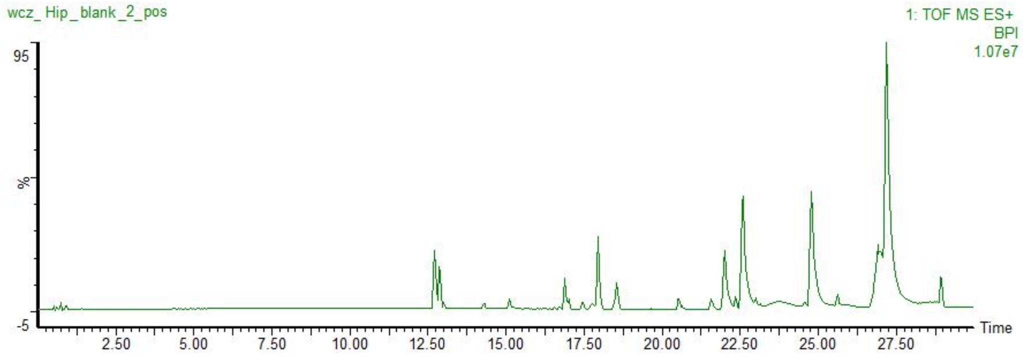

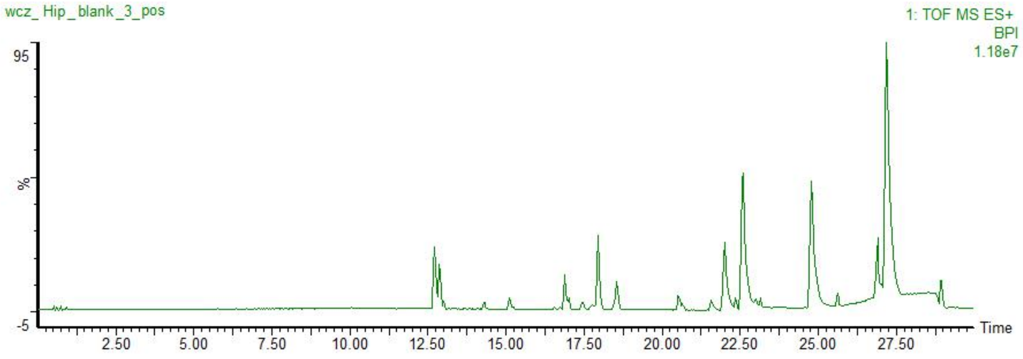

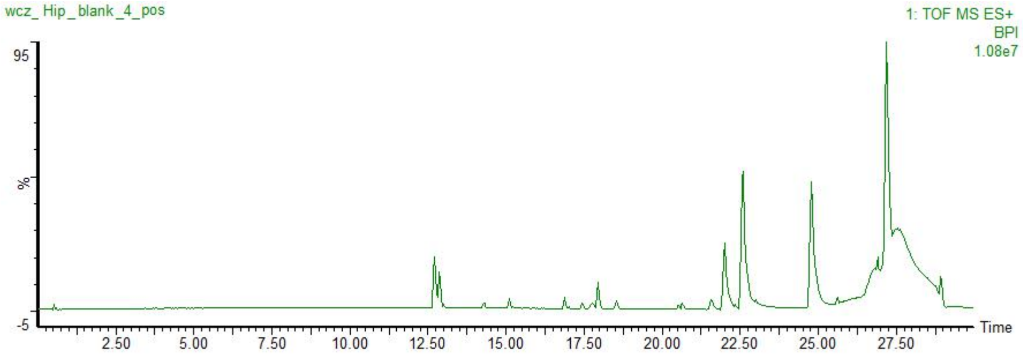

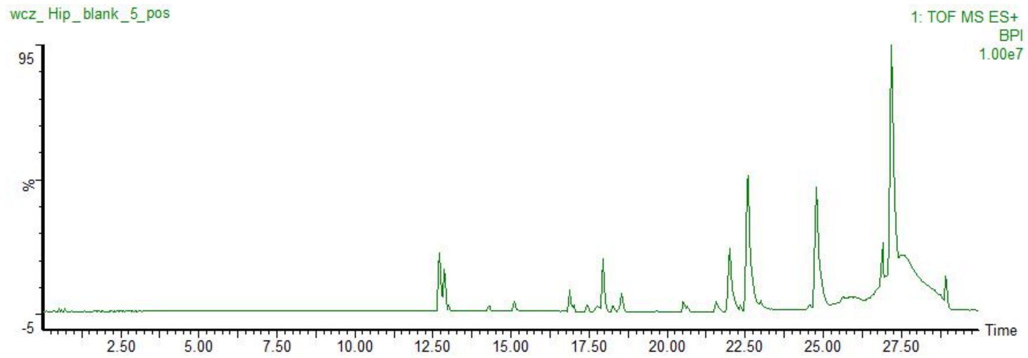
wcz_ Hip_ blank_5_pos
1: TOF MS ES+
BPI
1.00e7
95
%
-5
2.50
5.00
7.50
10.00
12.50
15.00
17.50
20.00
22.50
25.00
27.50
Time

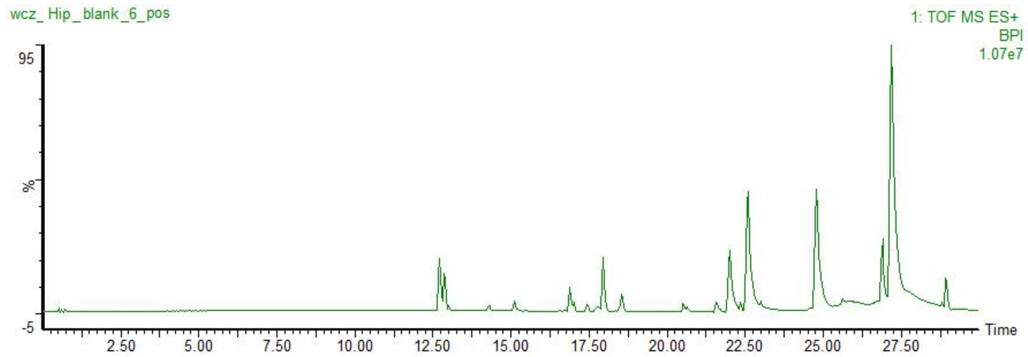
wcz_ Hip_ blank_6_pos
1: TOF MS ES+
BPI
1.07e7
95
%
-5
2.50
5.00
7.50
10.00
12.50
15.00
17.50
20.00
22.50
25.00
27.50
Time

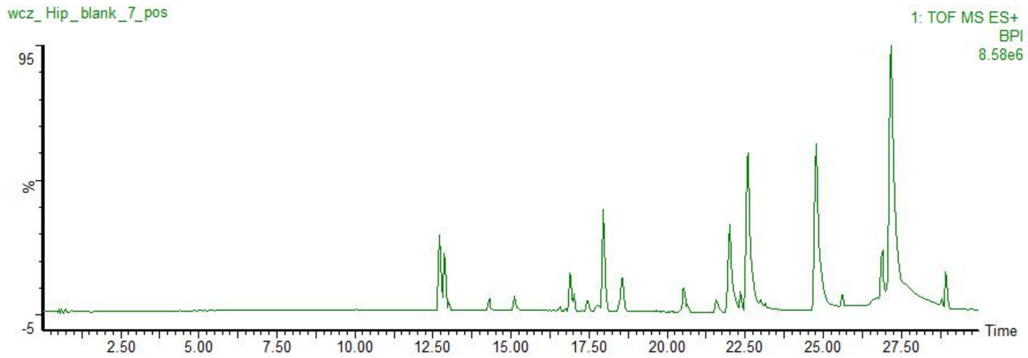
wcz_ Hip_ blank_7_pos
1: TOF MS ES+
BPI
8.58e6
95
%
-5
2.50
5.00
7.50
10.00
12.50
15.00
17.50
20.00
22.50
25.00
27.50
Time

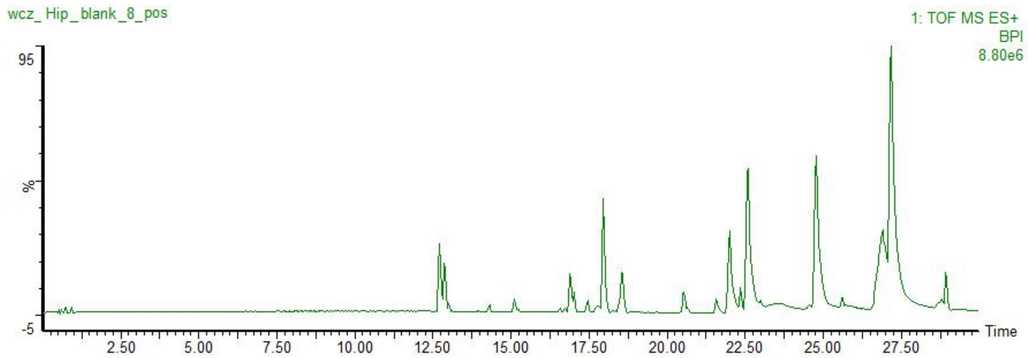
wcz_ Hip_ blank_8_pos
1: TOF MS ES+
BPI
8.80e6
95
%
-5
2.50
5.00
7.50
10.00
12.50
15.00
17.50
20.00
22.50
25.00
27.50
Time

5. **ESI+/ hip/ model group**

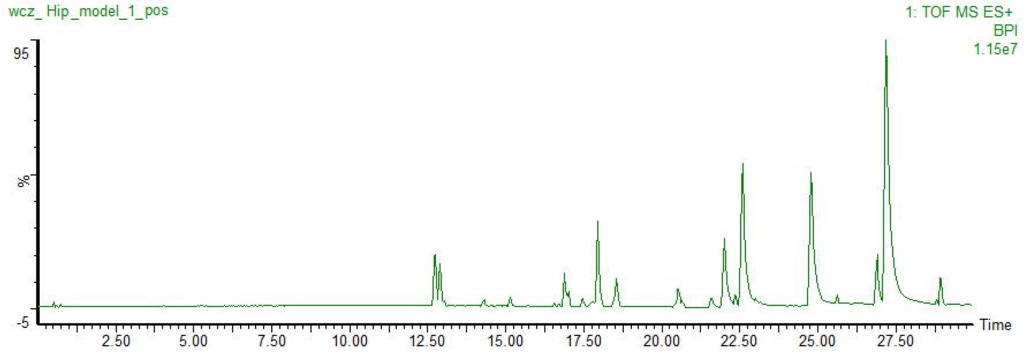

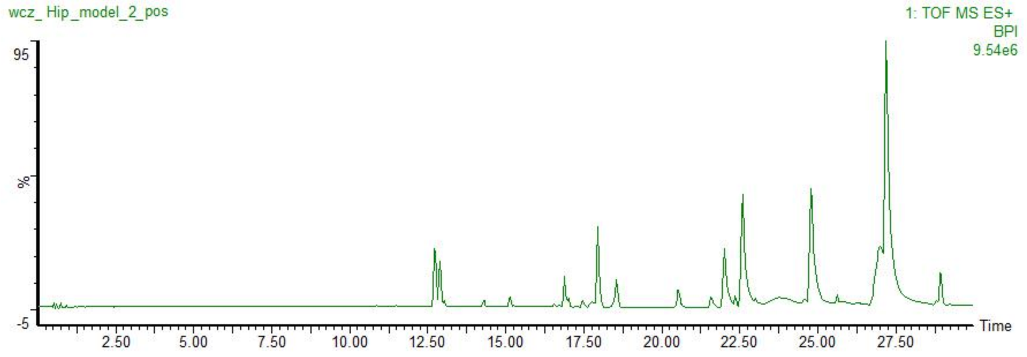

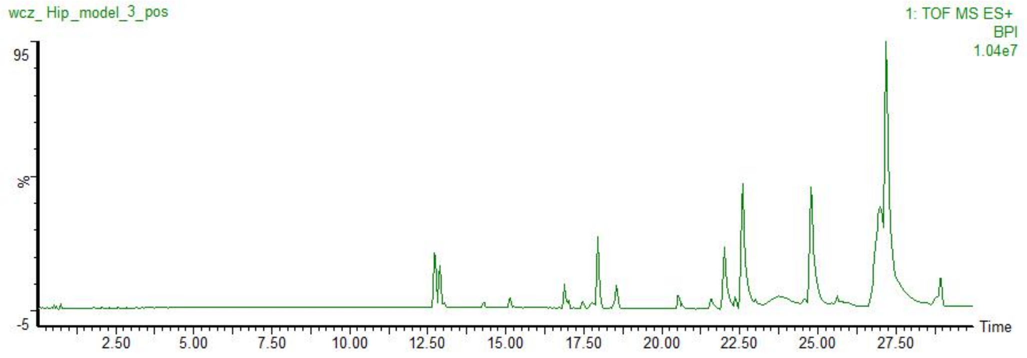

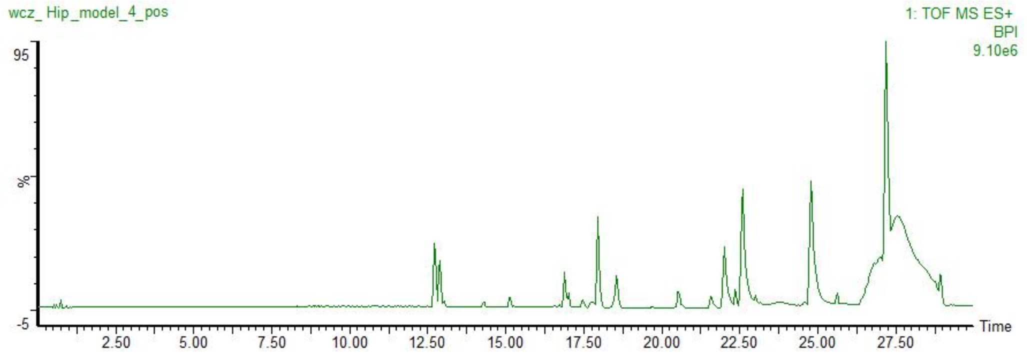

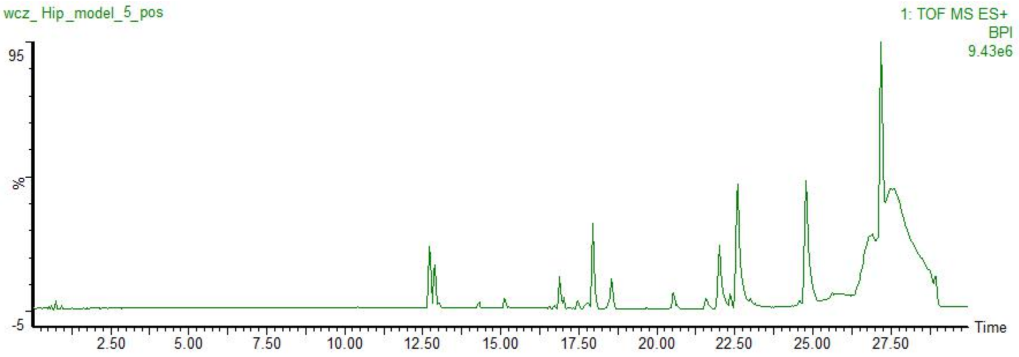
wcz_ Hip_model_5_pos
1: TOF MS ES+
BPI
9.43e6
95
%
-5
2.50
5.00
7.50
10.00
12.50
15.00
17.50
20.00
22.50
25.00
27.50
Time

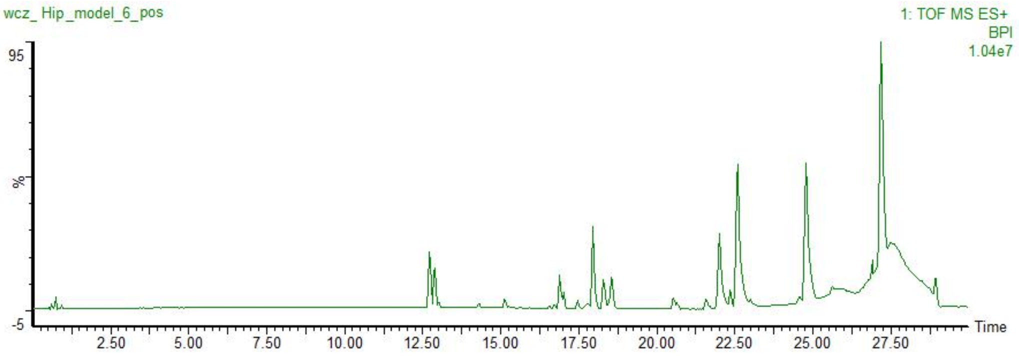
wcz_ Hip_model_6_pos
1: TOF MS ES+
BPI
1.04e7
95
%
-5
2.50
5.00
7.50
10.00
12.50
15.00
17.50
20.00
22.50
25.00
27.50
Time

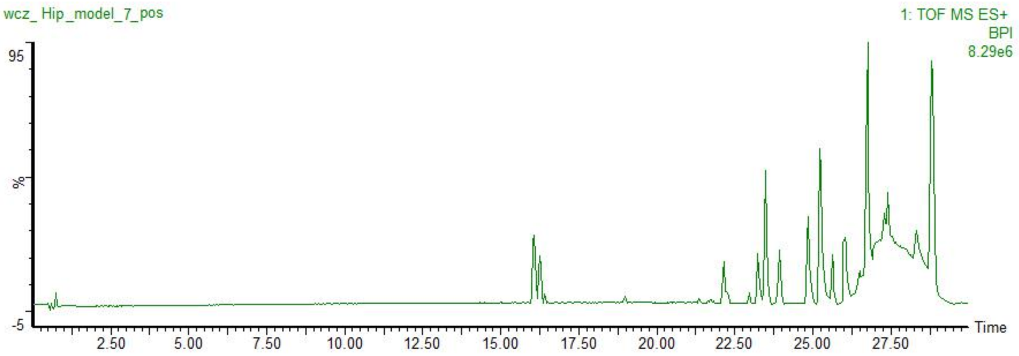
wcz_ Hip_model_7_pos
1: TOF MS ES+
BPI
8.29e6
95
%
-5
2.50
5.00
7.50
10.00
12.50
15.00
17.50
20.00
22.50
25.00
27.50
Time

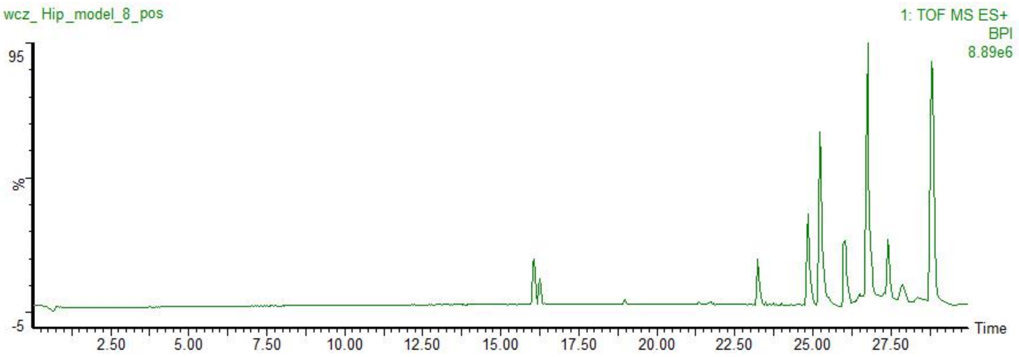
wcz_ Hip_model_8_pos
1: TOF MS ES+
BPI
8.89e6
95
%
-5
2.50
5.00
7.50
10.00
12.50
15.00
17.50
20.00
22.50
25.00
27.50
Time

6. ESI+/ hip/ HPF group

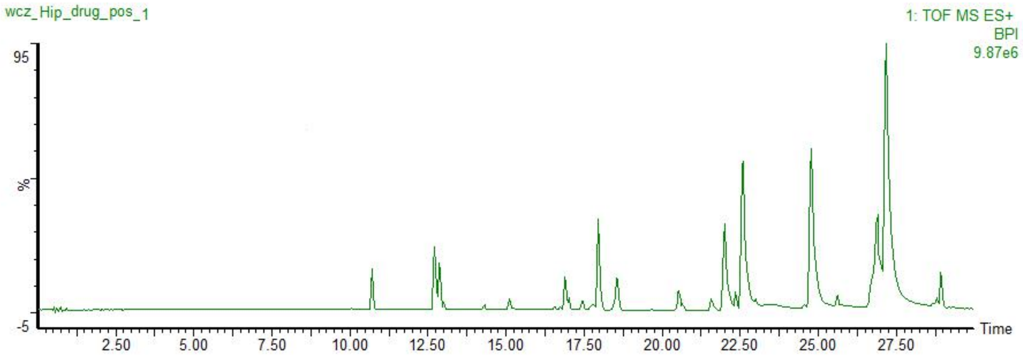

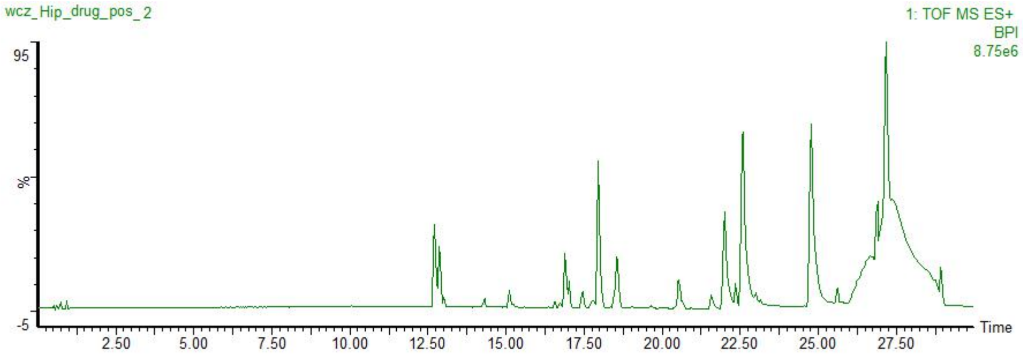

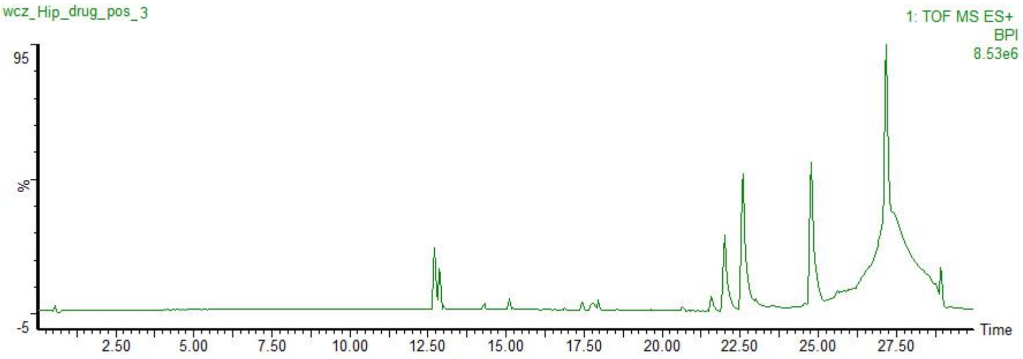

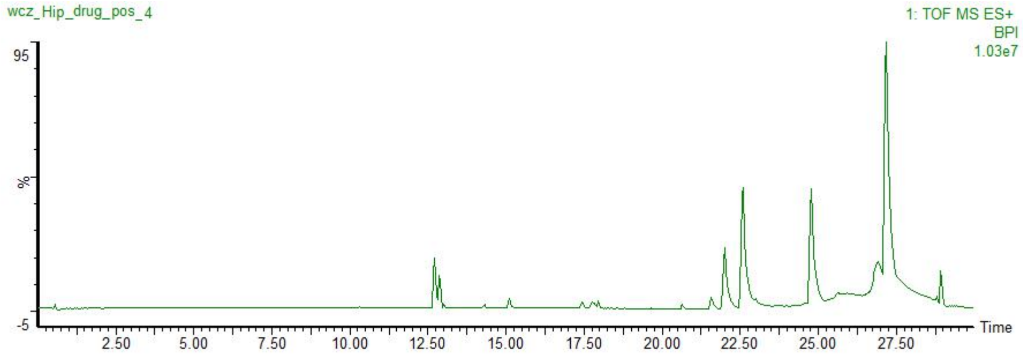

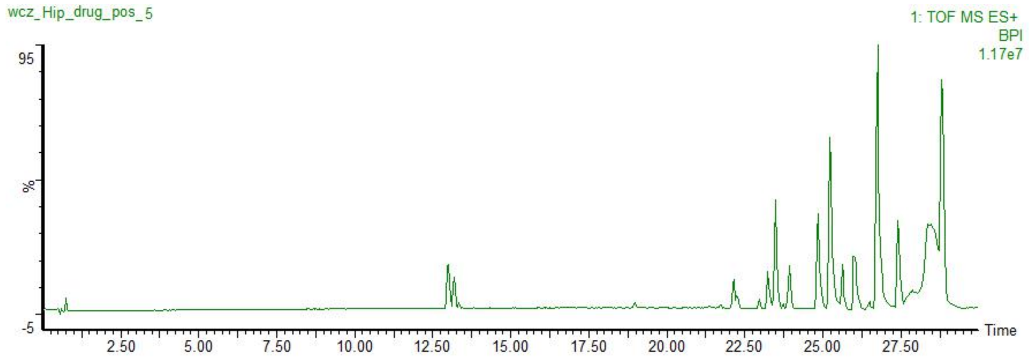
wcz_Hip_drug_pos_5
1: TOF MS ES+
BPI
1.17e7
95
%
-5
2.50 5.00 7.50 10.00 12.50 15.00 17.50 20.00 22.50 25.00 27.50
Time

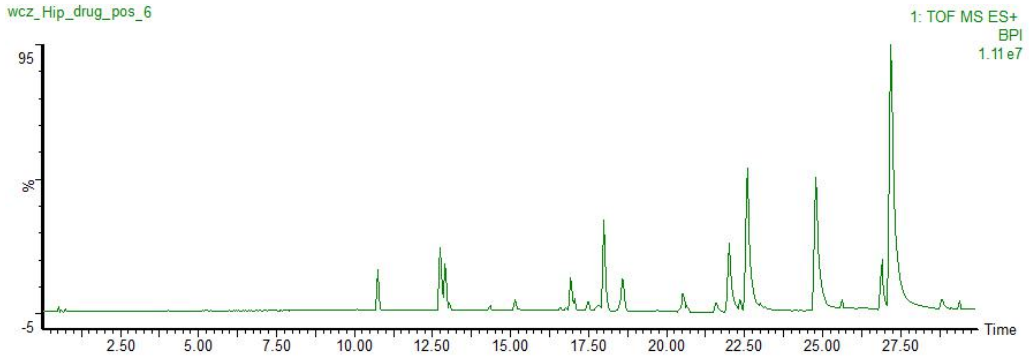
wcz_Hip_drug_pos_6
1: TOF MS ES+
BPI
1.11e7
95
%
-5
2.50 5.00 7.50 10.00 12.50 15.00 17.50 20.00 22.50 25.00 27.50
Time

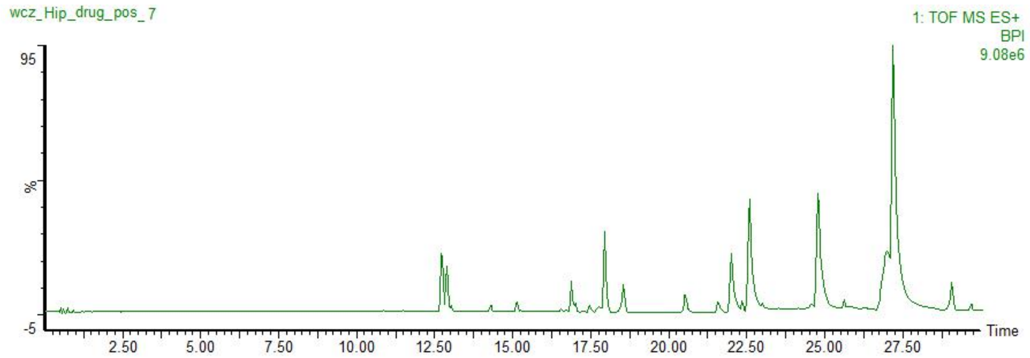
wcz_Hip_drug_pos_7
1: TOF MS ES+
BPI
9.08e6
95
%
-5
2.50 5.00 7.50 10.00 12.50 15.00 17.50 20.00 22.50 25.00 27.50
Time

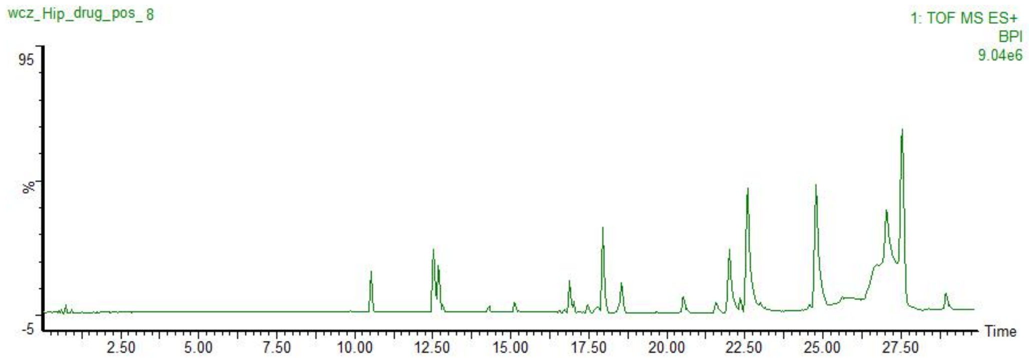
wcz_Hip_drug_pos_8
1: TOF MS ES+
BPI
9.04e6
95
%
-5
2.50 5.00 7.50 10.00 12.50 15.00 17.50 20.00 22.50 25.00 27.50
Time

7. **ESI-/ serum/ normal control group**

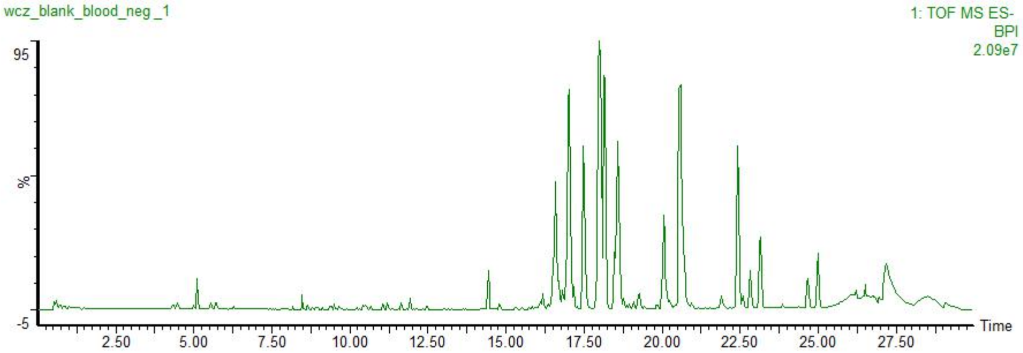

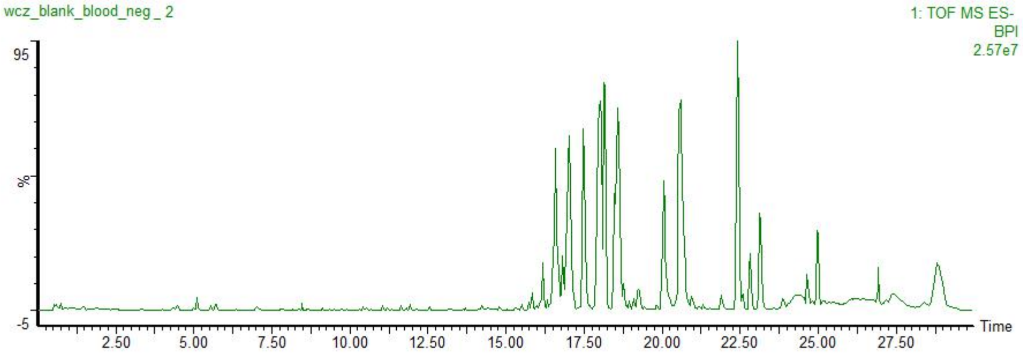

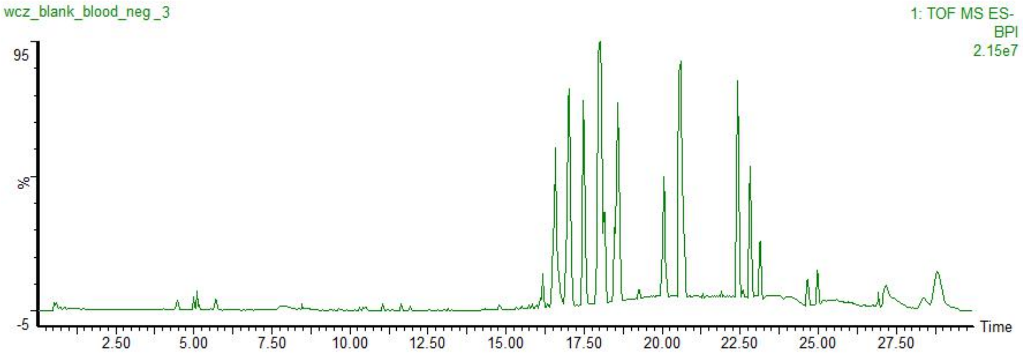

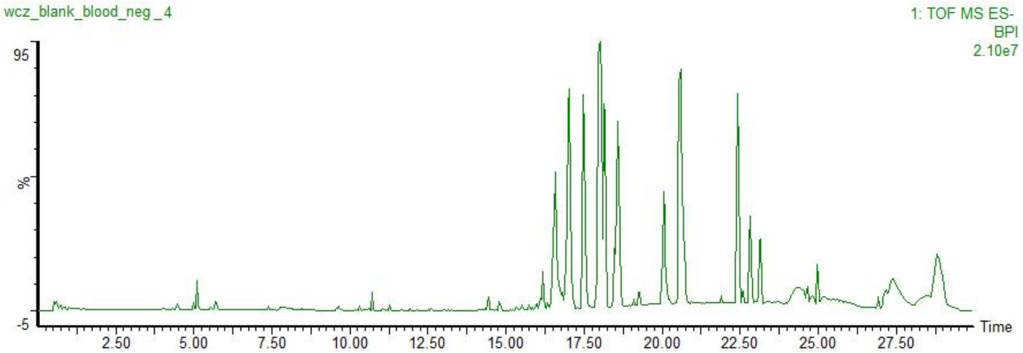

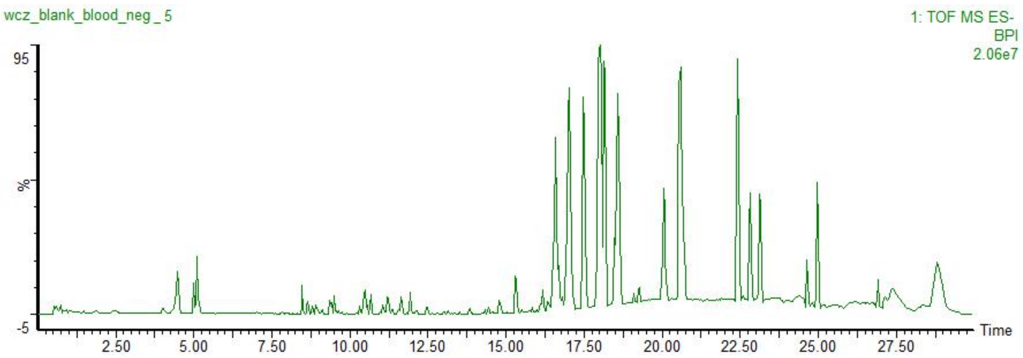
wcz_blank_blood_neg _5
1: TOF MS ES-
BPI
2.06e7
95
%
-5
2.50
5.00
7.50
10.00
12.50
15.00
17.50
20.00
22.50
25.00
27.50
Time

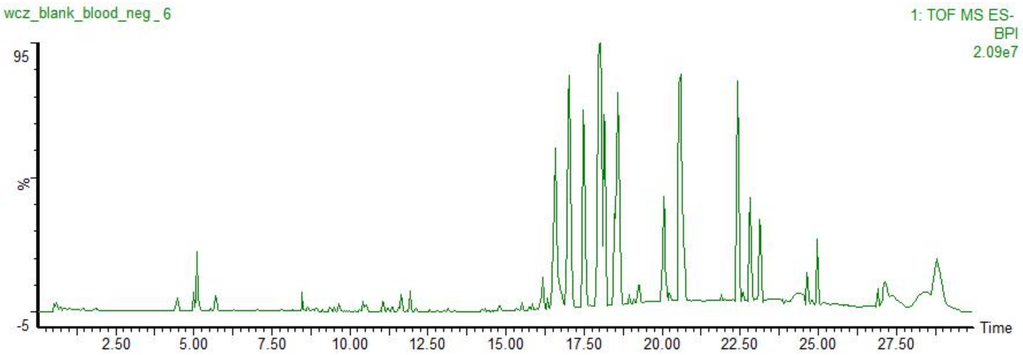
wcz_blank_blood_neg _6
1: TOF MS ES-
BPI
2.09e7
95
%
-5
2.50
5.00
7.50
10.00
12.50
15.00
17.50
20.00
22.50
25.00
27.50
Time

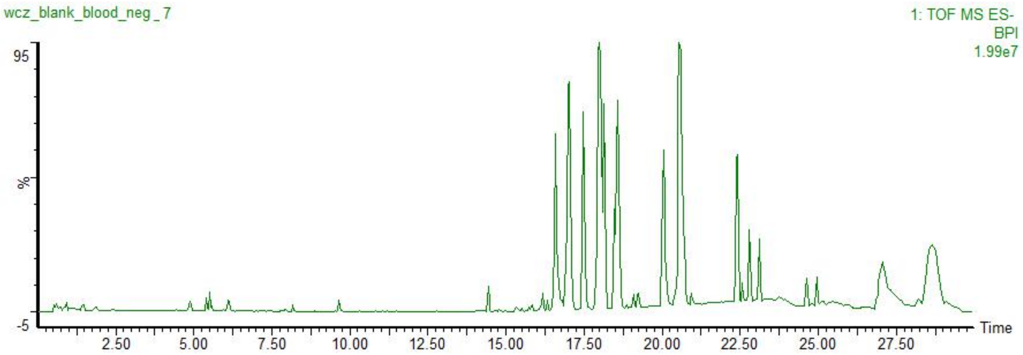
wcz_blank_blood_neg _7
1: TOF MS ES-
BPI
1.99e7
95
%
-5
2.50
5.00
7.50
10.00
12.50
15.00
17.50
20.00
22.50
25.00
27.50
Time

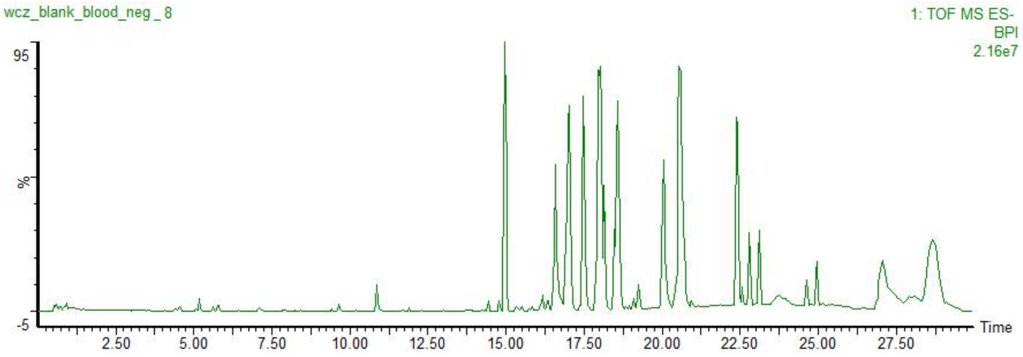
wcz_blank_blood_neg _8
1: TOF MS ES-
BPI
2.16e7
95
%
-5
2.50
5.00
7.50
10.00
12.50
15.00
17.50
20.00
22.50
25.00
27.50
Time

8. ESI-/ serum/ model group

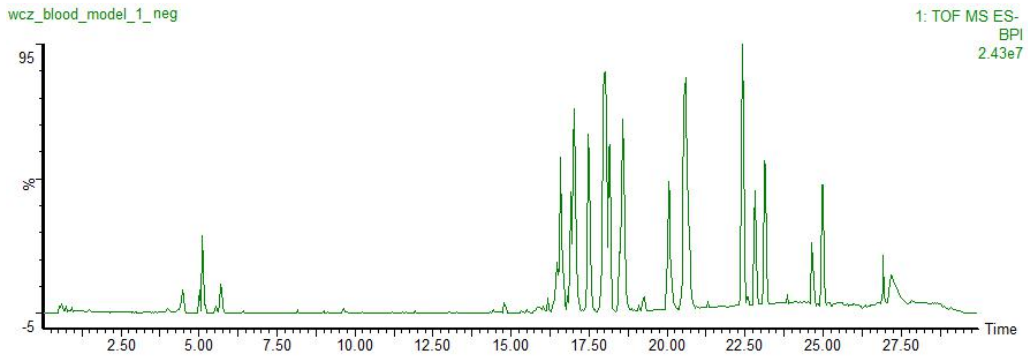

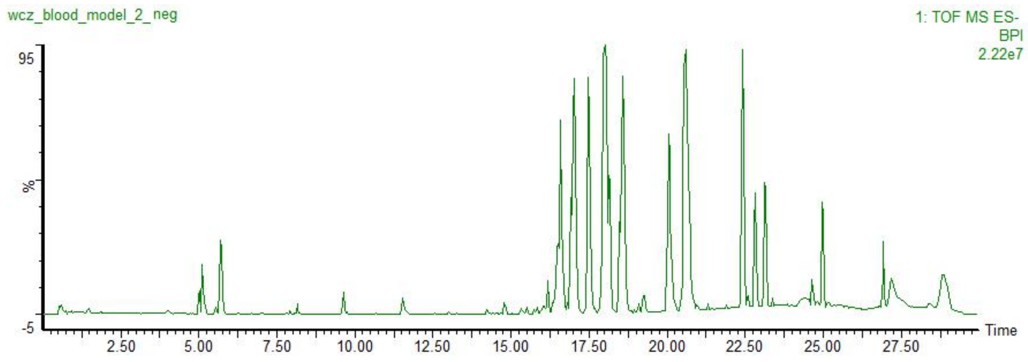

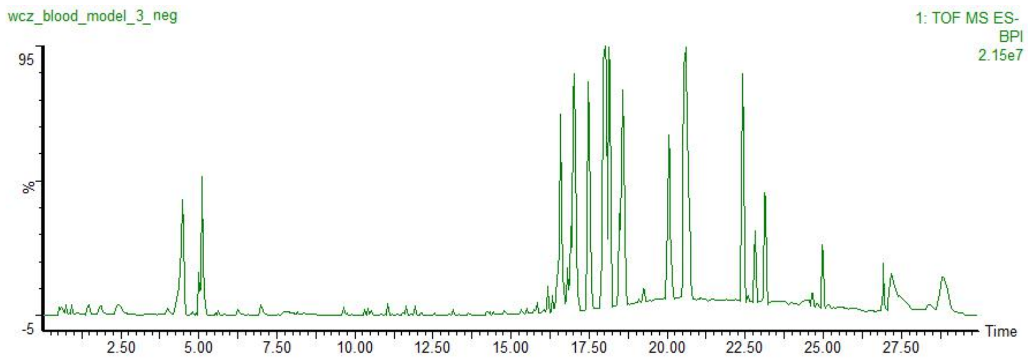

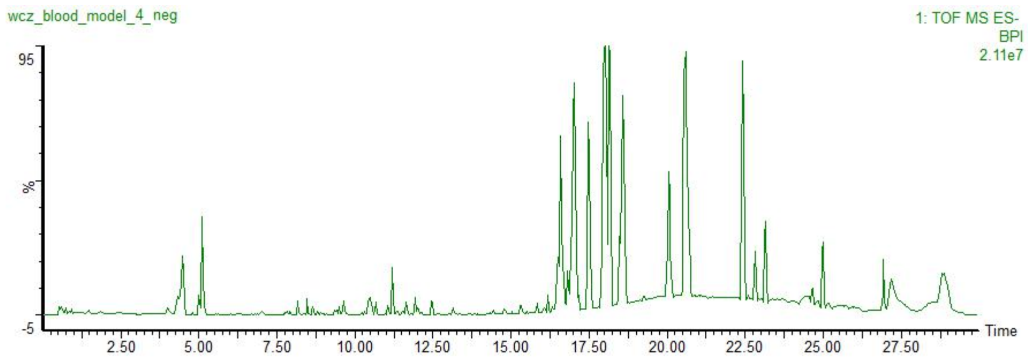

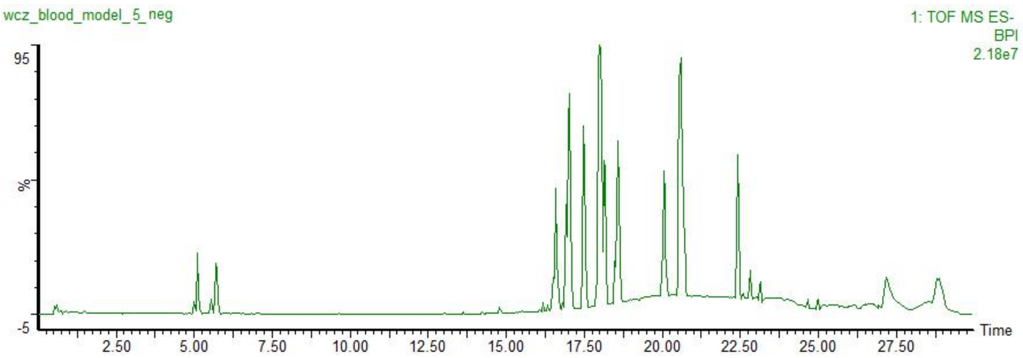
wcz_blood_model_5_neg
1: TOF MS ES-
BPI
2.18e7
95
%
-5
2.50 5.00 7.50 10.00 12.50 15.00 17.50 20.00 22.50 25.00 27.50
Time

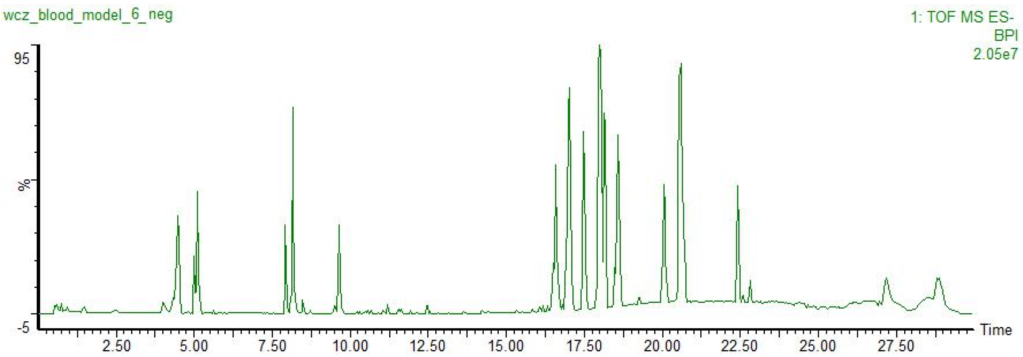
wcz_blood_model_6_neg
1: TOF MS ES-
BPI
2.05e7
95
%
-5
2.50 5.00 7.50 10.00 12.50 15.00 17.50 20.00 22.50 25.00 27.50
Time

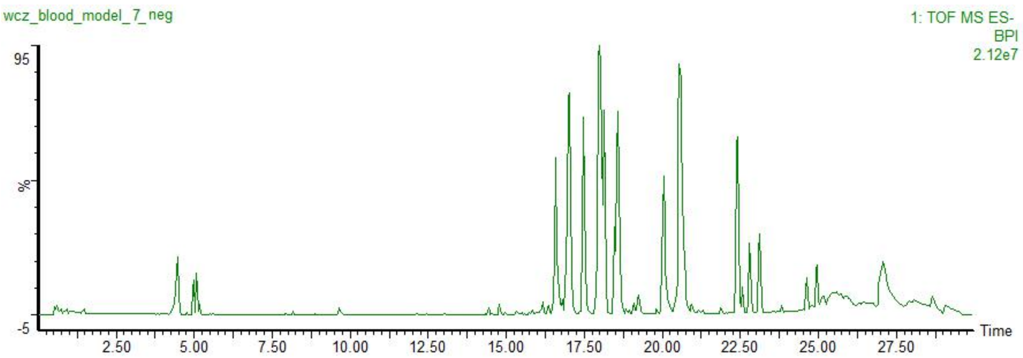
wcz_blood_model_7_neg
1: TOF MS ES-
BPI
2.12e7
95
%
-5
2.50 5.00 7.50 10.00 12.50 15.00 17.50 20.00 22.50 25.00 27.50
Time

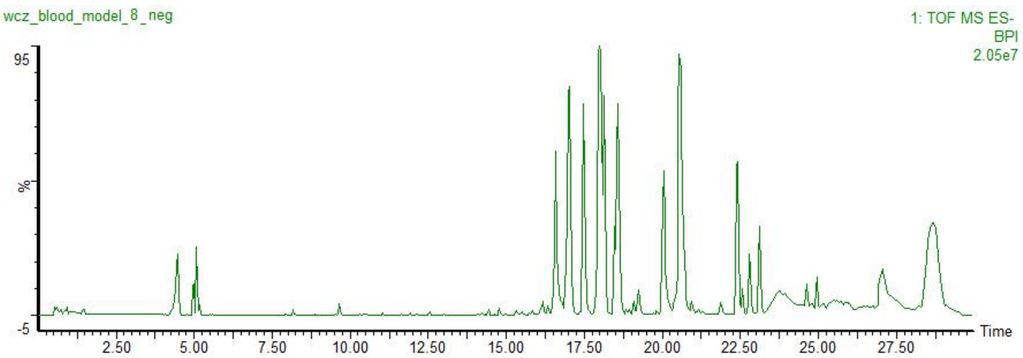
wcz_blood_model_8_neg
1: TOF MS ES-
BPI
2.05e7
95
%
-5
2.50 5.00 7.50 10.00 12.50 15.00 17.50 20.00 22.50 25.00 27.50
Time

9. ESI-/ serum/ HPF group

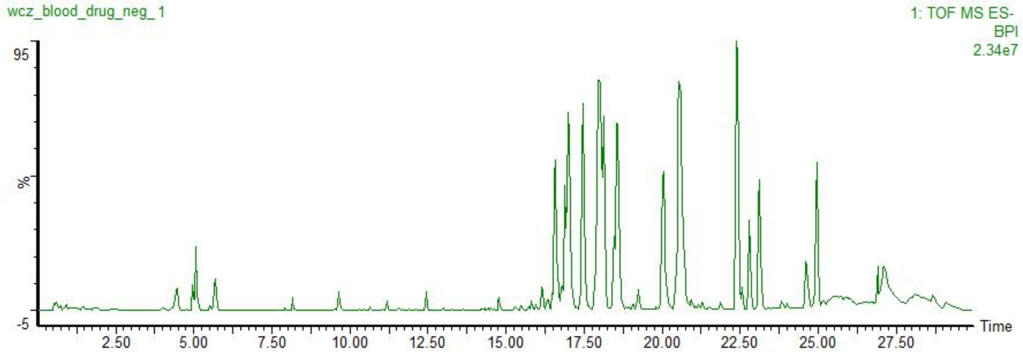

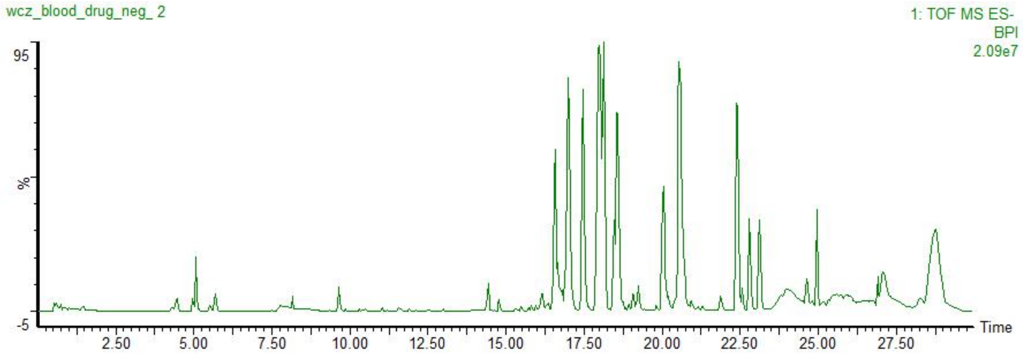

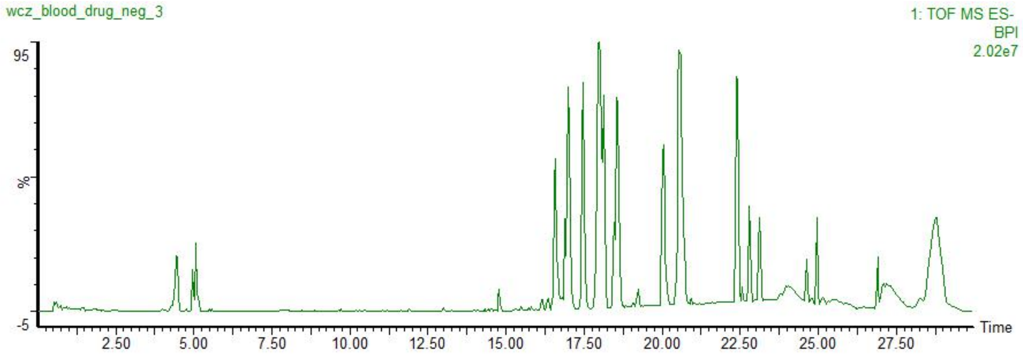

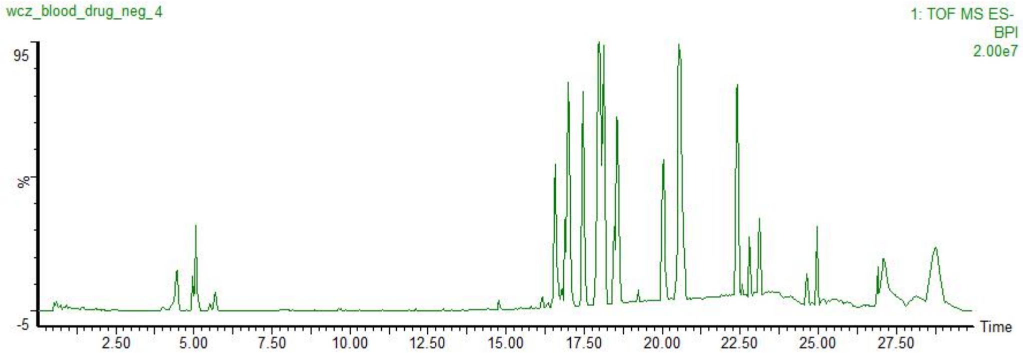

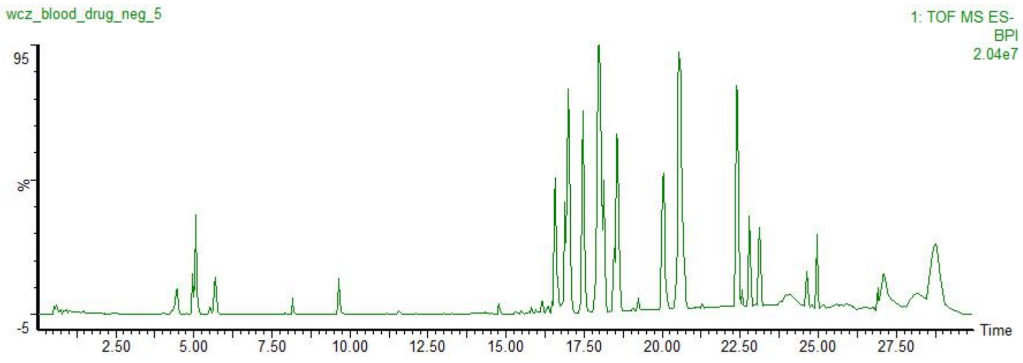
wcz_blood_drug_neg_5
1: TOF MS ES-
BPI
2.04e7
95
%
-5
2.50
5.00
7.50
10.00
12.50
15.00
17.50
20.00
22.50
25.00
27.50
Time

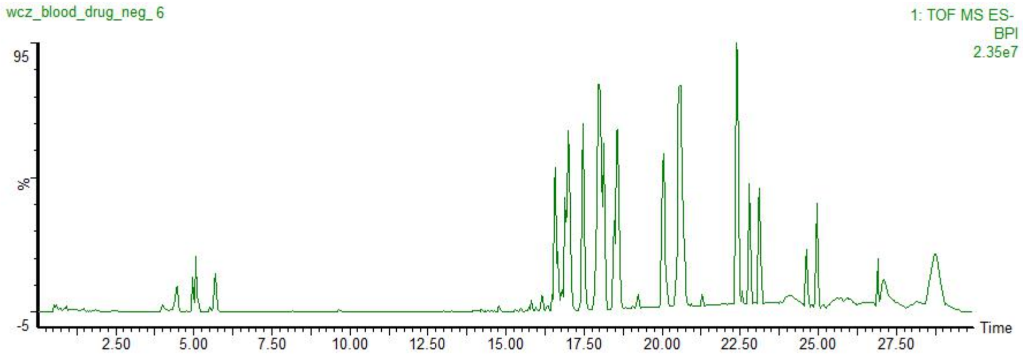
wcz_blood_drug_neg_6
1: TOF MS ES-
BPI
2.35e7
95
%
-5
2.50
5.00
7.50
10.00
12.50
15.00
17.50
20.00
22.50
25.00
27.50
Time

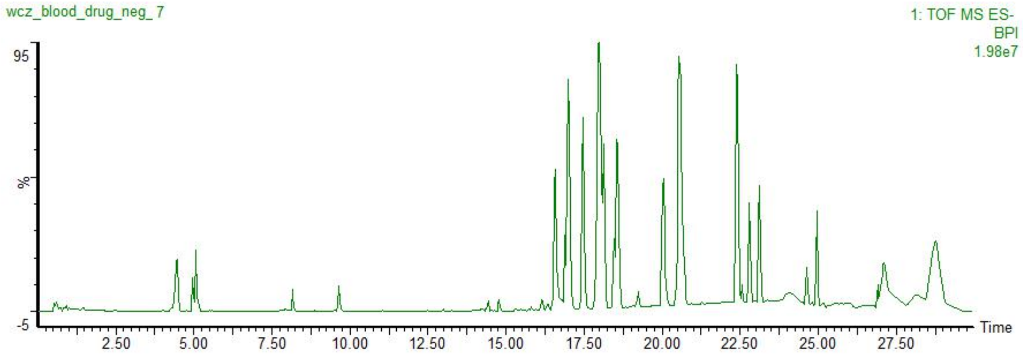
wcz_blood_drug_neg_7
1: TOF MS ES-
BPI
1.98e7
95
%
-5
2.50
5.00
7.50
10.00
12.50
15.00
17.50
20.00
22.50
25.00
27.50
Time

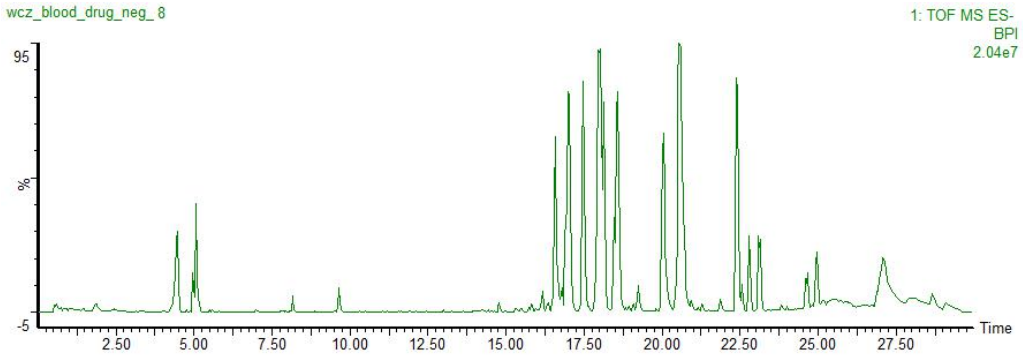
wcz_blood_drug_neg_8
1: TOF MS ES-
BPI
2.04e7
95
%
-5
2.50
5.00
7.50
10.00
12.50
15.00
17.50
20.00
22.50
25.00
27.50
Time

10. ESI-/ hip/ normal control group

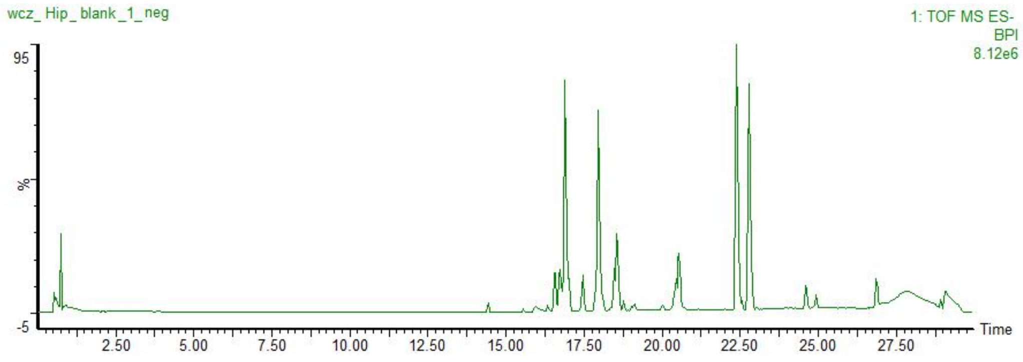

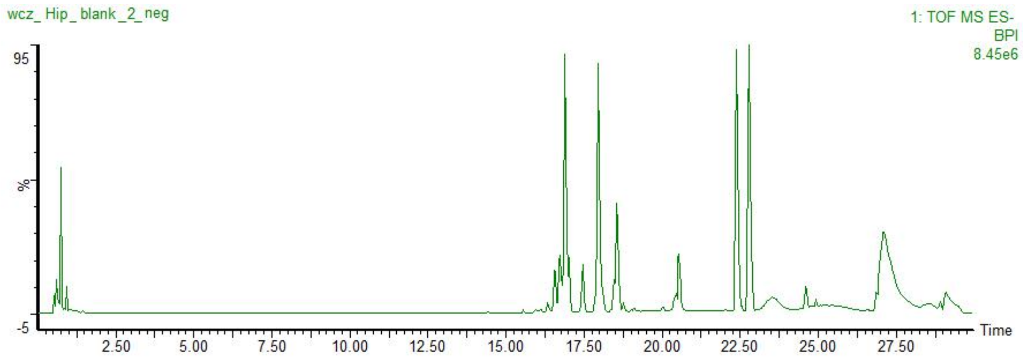

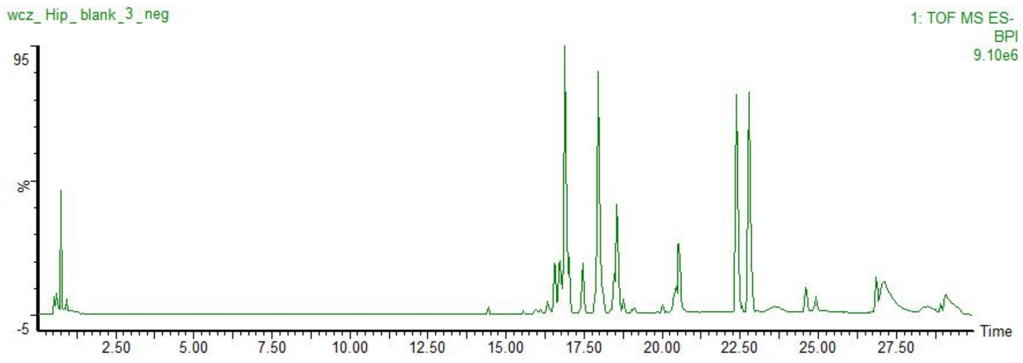

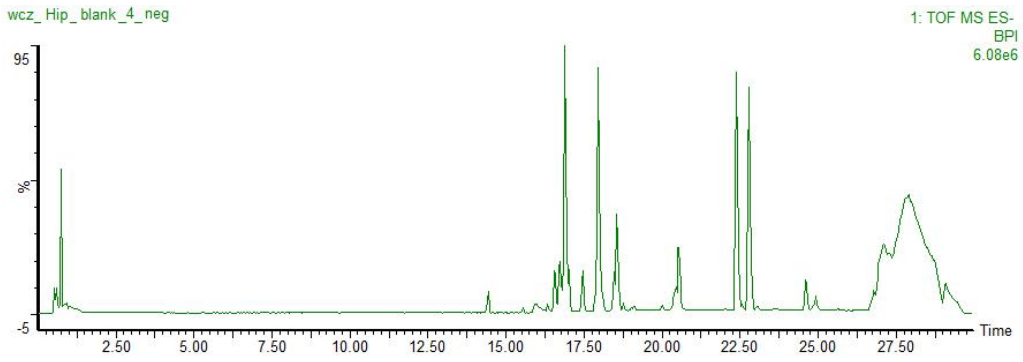

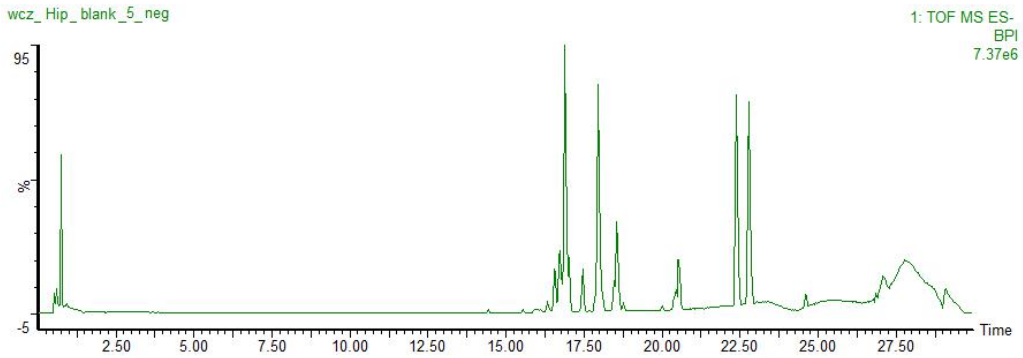
wcz_ Hip_ blank_5_ neg
1: TOF MS ES-
BPI
7.37e6
95
%
-5
2.50
5.00
7.50
10.00
12.50
15.00
17.50
20.00
22.50
25.00
27.50
Time

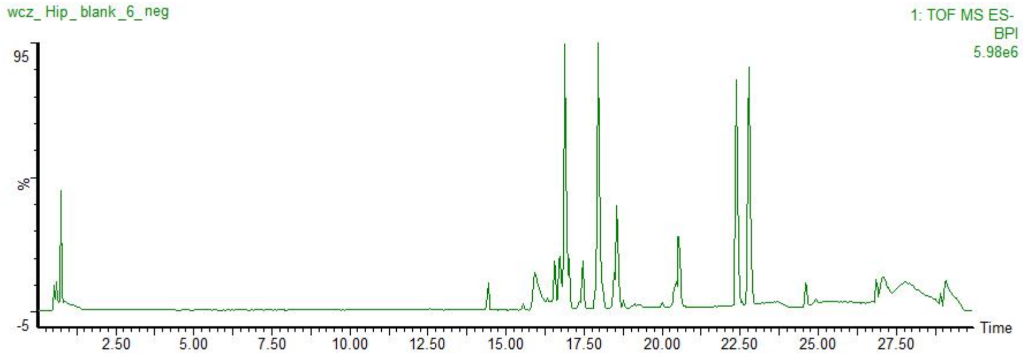
wcz_ Hip_ blank_6_ neg
1: TOF MS ES-
BPI
5.98e6
95
%
-5
2.50
5.00
7.50
10.00
12.50
15.00
17.50
20.00
22.50
25.00
27.50
Time

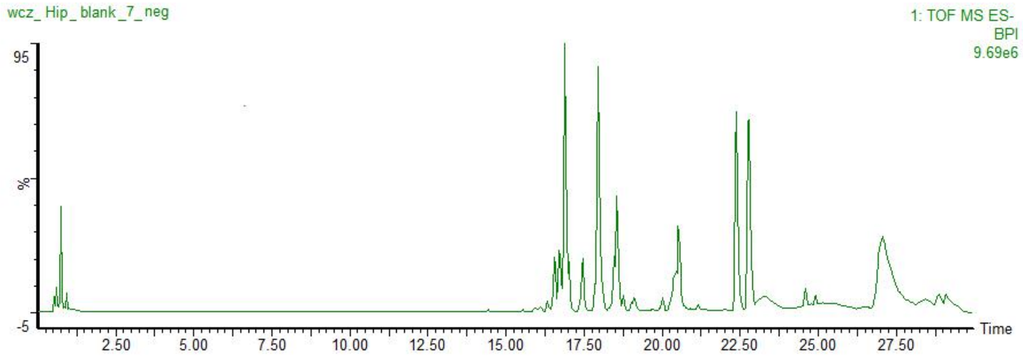
wcz_ Hip_ blank_7_ neg
1: TOF MS ES-
BPI
9.69e6
95
%
-5
2.50
5.00
7.50
10.00
12.50
15.00
17.50
20.00
22.50
25.00
27.50
Time

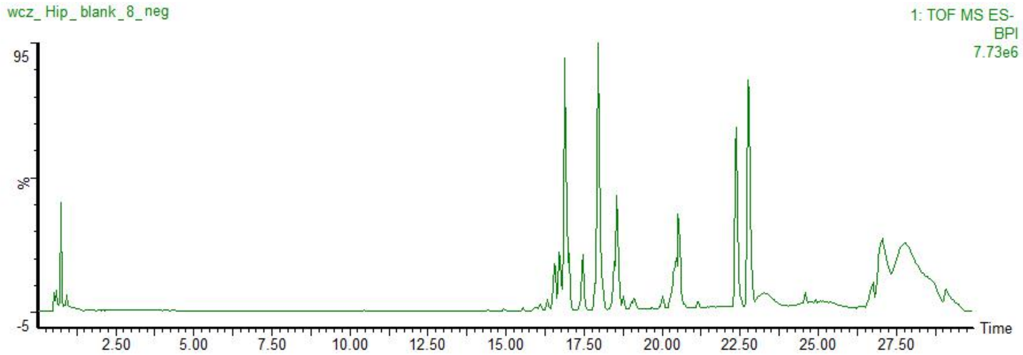
wcz_ Hip_ blank_8_ neg
1: TOF MS ES-
BPI
7.73e6
95
%
-5
2.50
5.00
7.50
10.00
12.50
15.00
17.50
20.00
22.50
25.00
27.50
Time

11. ESI-/ hip/ model group

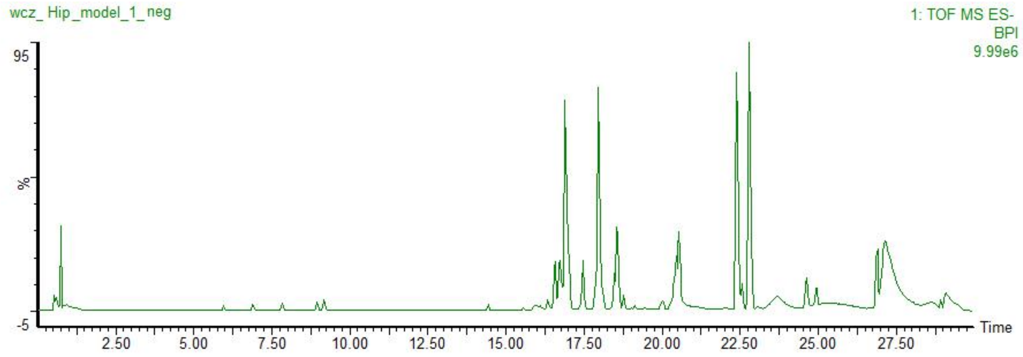

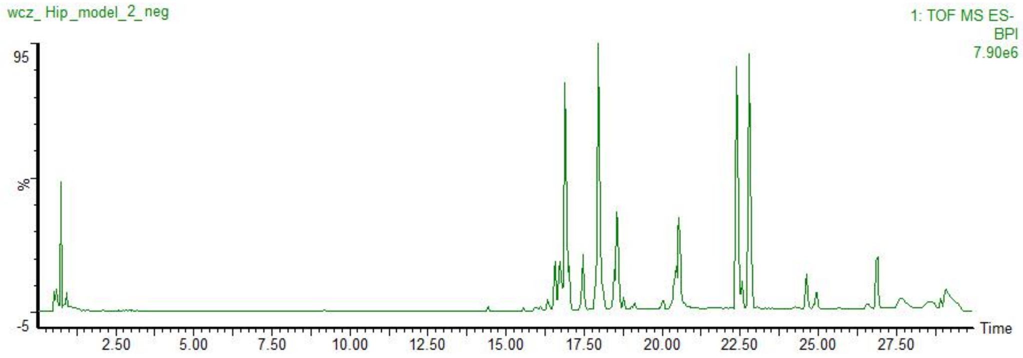

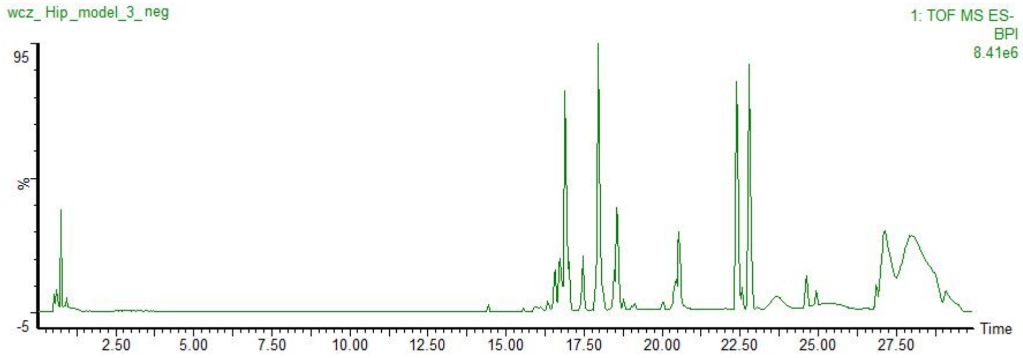

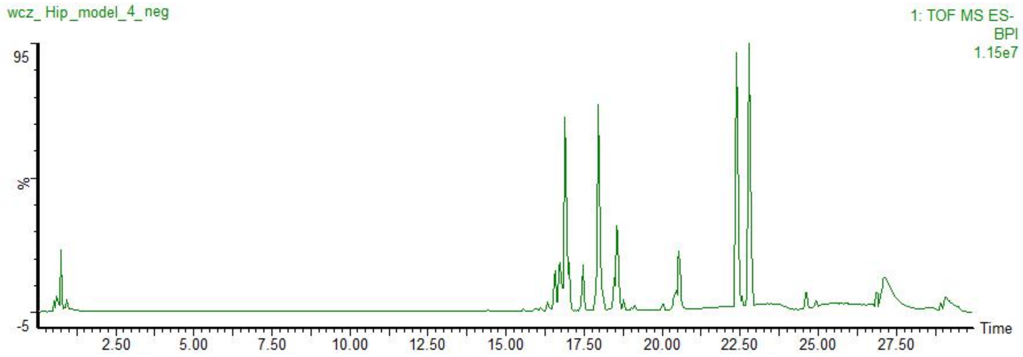

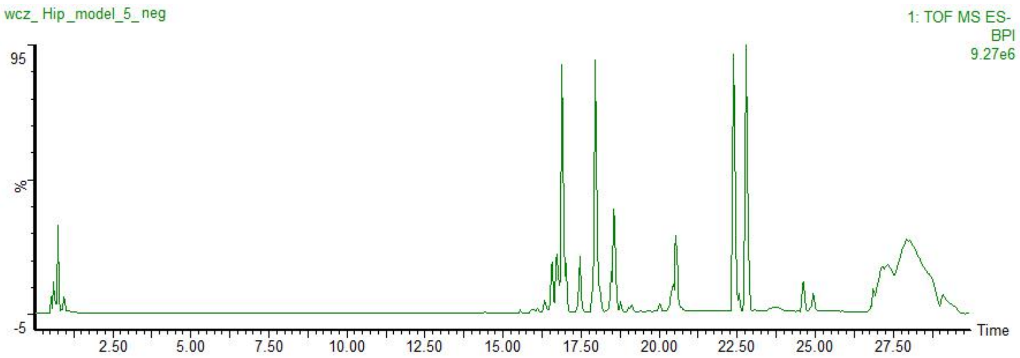
wcz_ Hip_model_5_neg
1: TOF MS ES-
BPI
9.27e6
95
%
-5
2.50
5.00
7.50
10.00
12.50
15.00
17.50
20.00
22.50
25.00
27.50
Time

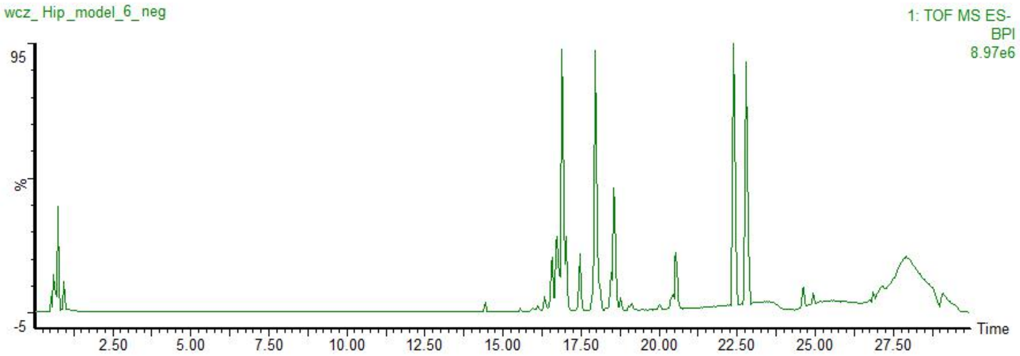
wcz_ Hip_model_6_neg
1: TOF MS ES-
BPI
8.97e6
95
%
-5
2.50
5.00
7.50
10.00
12.50
15.00
17.50
20.00
22.50
25.00
27.50
Time

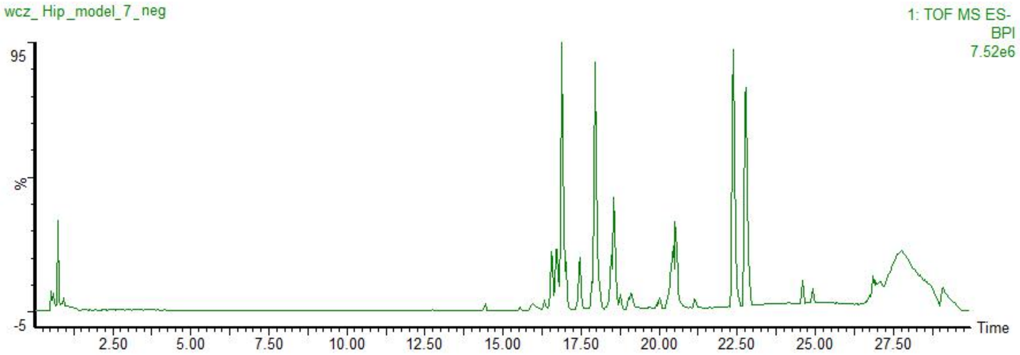
wcz_ Hip_model_7_neg
1: TOF MS ES-
BPI
7.52e6
95
%
-5
2.50
5.00
7.50
10.00
12.50
15.00
17.50
20.00
22.50
25.00
27.50
Time

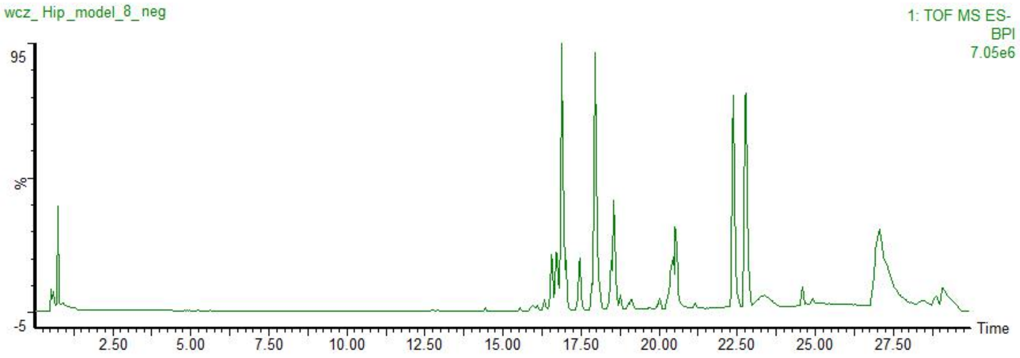
wcz_ Hip_model_8_neg
1: TOF MS ES-
BPI
7.05e6
95
%
-5
2.50
5.00
7.50
10.00
12.50
15.00
17.50
20.00
22.50
25.00
27.50
Time

12. ESI-/ hip/ HPF group

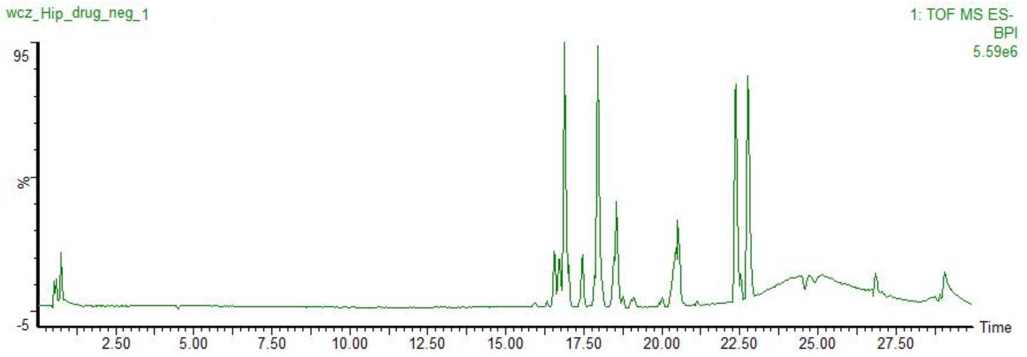

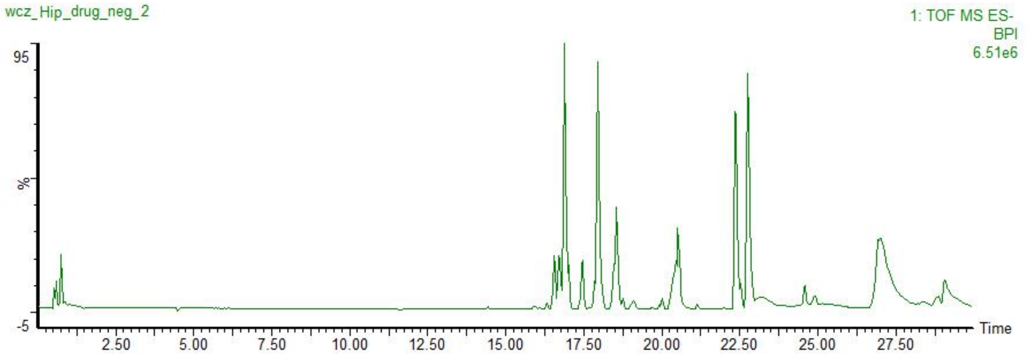

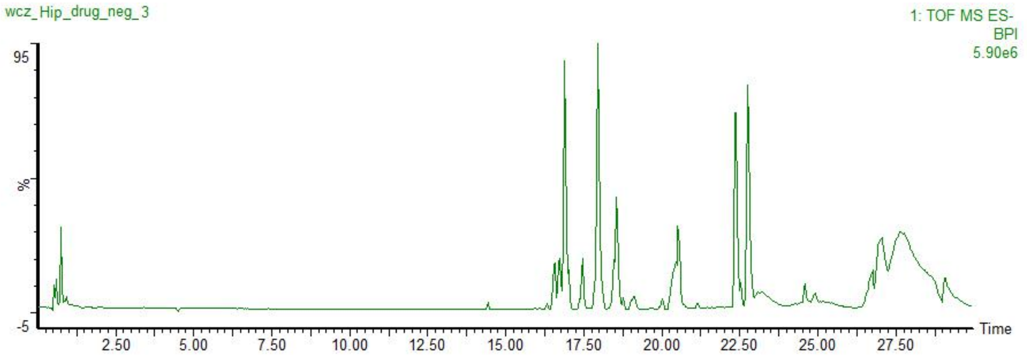

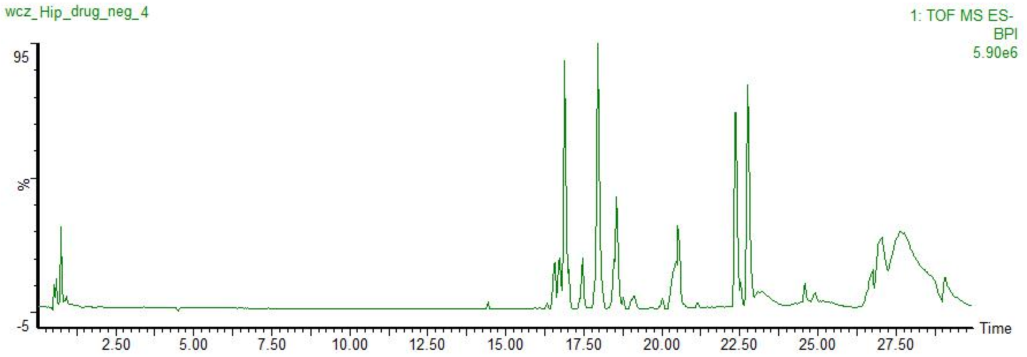

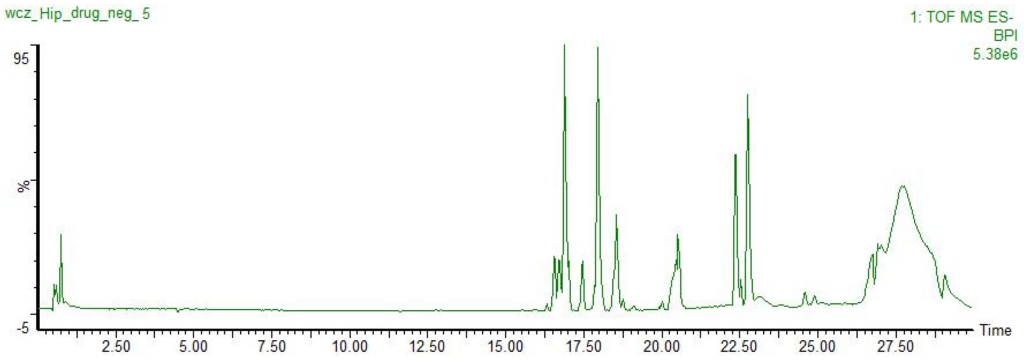
wcz_Hip_drug_neg_ 5
1: TOF MS ES-
BPI
5.38e6
95
%
-5
2.50 5.00 7.50 10.00 12.50 15.00 17.50 20.00 22.50 25.00 27.50
Time

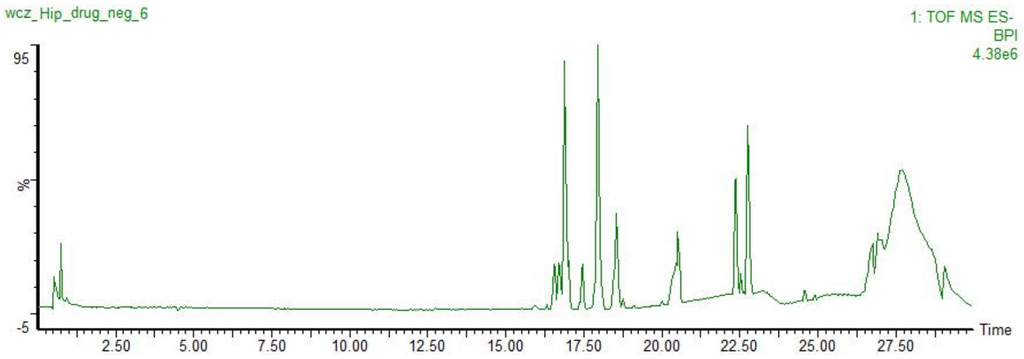
wcz_Hip_drug_neg_6
1: TOF MS ES-
BPI
4.38e6
95
%
-5
2.50 5.00 7.50 10.00 12.50 15.00 17.50 20.00 22.50 25.00 27.50
Time

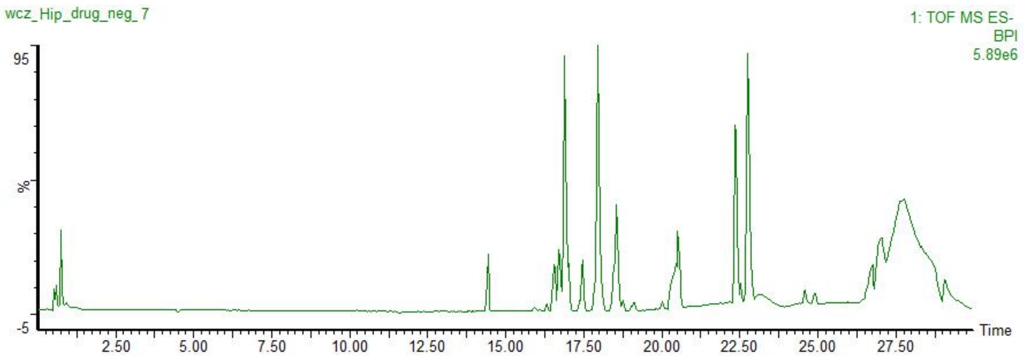
wcz_Hip_drug_neg_7
1: TOF MS ES-
BPI
5.89e6
95
%
-5
2.50 5.00 7.50 10.00 12.50 15.00 17.50 20.00 22.50 25.00 27.50
Time

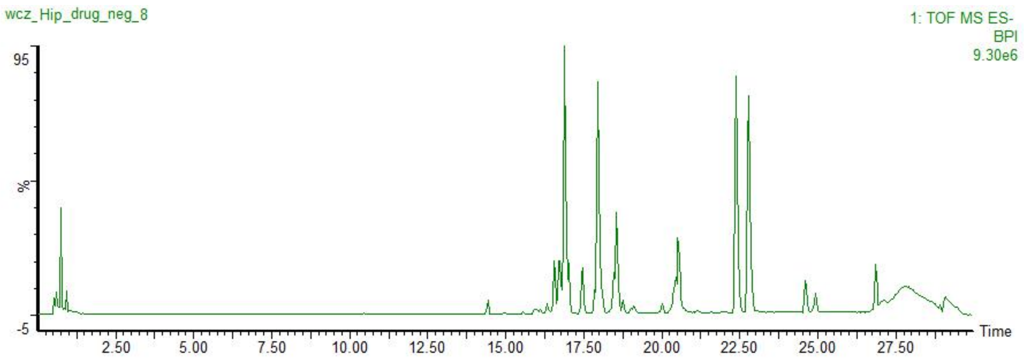
wcz_Hip_drug_neg_8
1: TOF MS ES-
BPI
9.30e6
95
%
-5
2.50 5.00 7.50 10.00 12.50 15.00 17.50 20.00 22.50 25.00 27.50
Time

Supplementary Materials II

MS Spectra of Each Potential Biomarker in Function 1 (Low Energy) and Function 2 (High Energy)

1. MS Spectra of *Glycerophosphocholine* in Function 1 and Function 2.
2. MS Spectra of *Myoinositol* in Function 1 and Function 2.
3. MS Spectra of *α-Ketoglutarate* in Function 1 and Function 2.
4. MS Spectra of *Glutathione* in Function 1 and Function 2.MS Spectra of *Citric Acid* in Function 1 and Function 2.
5. MS Spectra of *Oxidized Glutathione* in Function 1 and Function 2.
6. MS Spectra of *Pantetheine 4′-phosphate* in Function 1 and Function 2.
7. MS Spectra of *Pantothenic acid* in Function 1 and Function 2.
8. MS Spectra of *L-Tryptophan* in Function 1 and Function 2.
9. MS Spectra of *Indoleacetaldehyde* in Function 1 and Function 2.
10. MS Spectra of *Phytosphingosin* in Function 1 and Function 2.
11. MS Spectra of *Sphinganine* in Function 1 and Function 2.
12. MS Spectra of *Sphingosine 1-phosphate* in Function 1 and Function 2.
13. MS Spectra of *Sphinganine 1-phosphate* in Function 1 and Function 2.
14. MS Spectra of *Leukotriene A4* in Function 1 and Function 2.
15. MS Spectra of *12,13-EpOME* in Function 1 and Function 2.
16. MS Spectra of *19 (S)-HETE* in Function 1 and Function 2.
17. MS Spectra of *LysoPC (18:1(9Z))* in Function 1 and Function 2.

18. MS Spectra of *18:1/18:3 phosphatidylcholine* in Function 1 and Function 2.

19. MS Spectra of *Arachidonic acid* in Function 1 and Function 2.

20. MS Spectra of *Linoleic acid* in Function 1 and Function 2.

21. MS Spectra of *20-Hydroxy-leukotriene B4* in Function 1 and Function 2.

22. MS Spectra of *18:1/18:2 phosphatidylcholine* in Function 1 and Function 2.

23. MS Spectra of *32 Pantetheine 4′-phosphate* in Function 1 and Function 2.

24. MS Spectra of *Lactosylceramide* in Function 1 and Function 2.

25. MS Spectra of *18:2/18:3 phosphatidylcholine* in Function 1 and Function 2.

26. MS Spectra of *SM (d18:0/16:1)* in Function 1 and Function 2.

27. MS Spectra of *16:0/18:1 phosphatidylcholine* in Function 1 and Function 2.

28. MS Spectra of *18:2/18:1 phosphatidylcholine* in Function 1 and Function 2.

1. MS Spectra of Glycerophosphocholine in Function 1 and Function 2

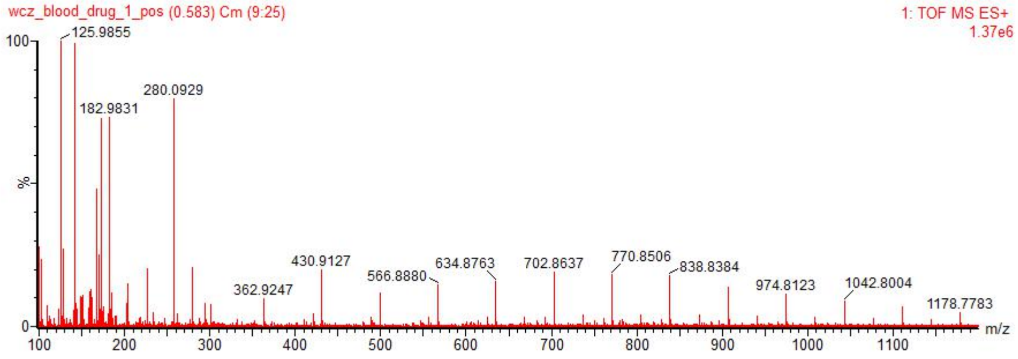

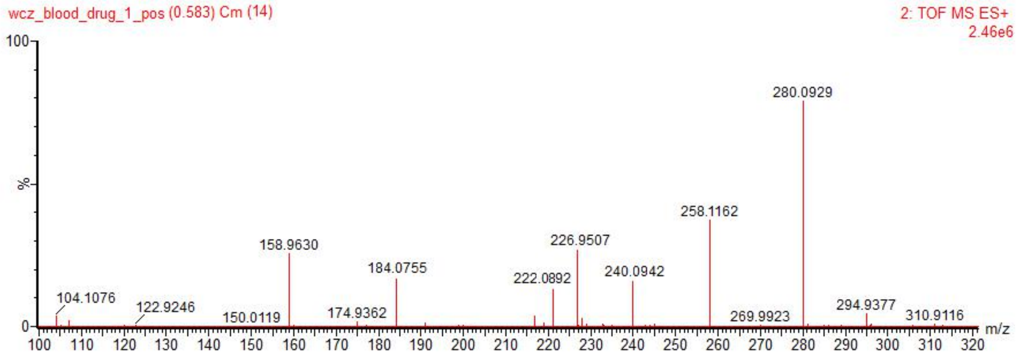

2. MS Spectra of Myoinositol in Function 1 and Function 2

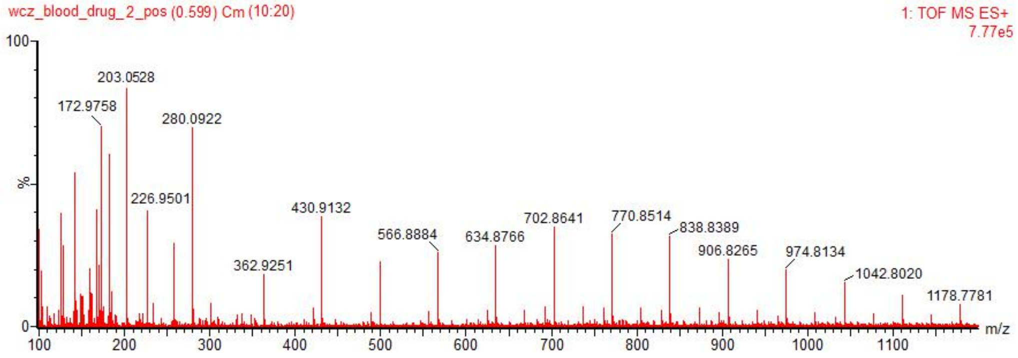

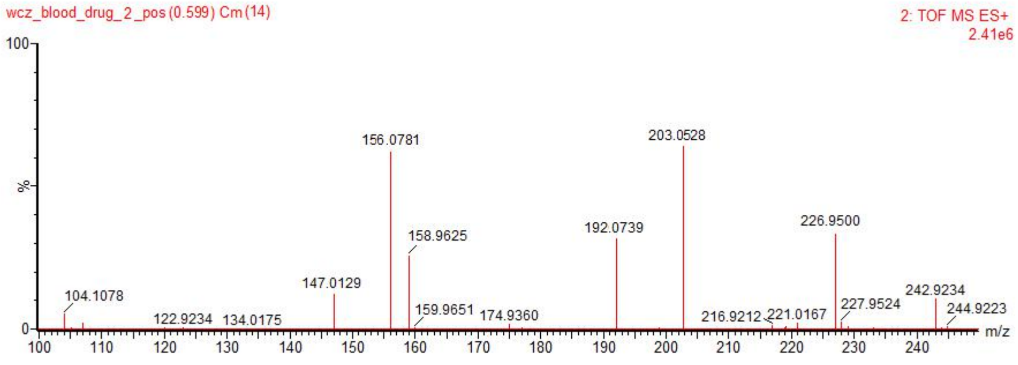

3. MS Spectra of α-Ketoglutarate in Function 1 and Function 2

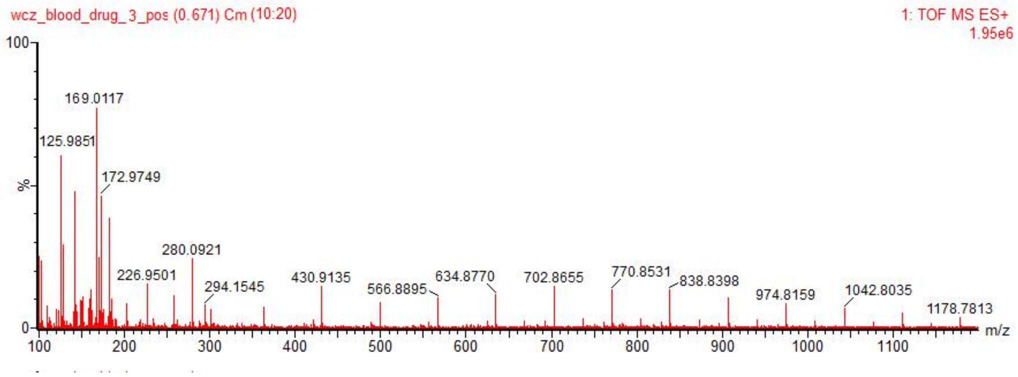

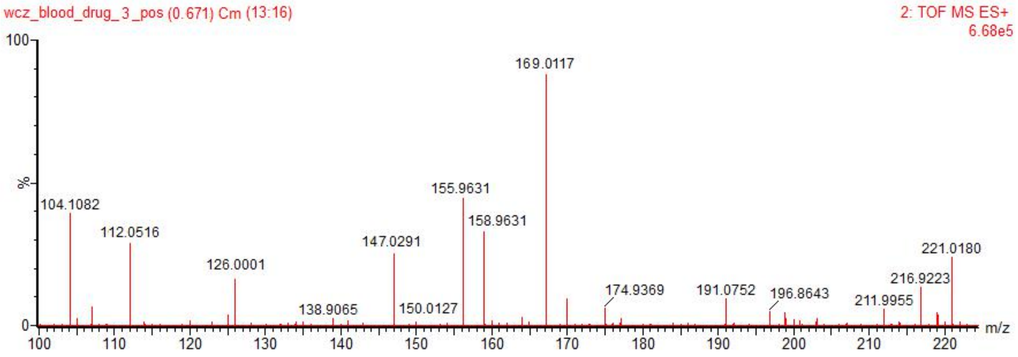

4. MS Spectra of Glutathione in Function 1 and Function 2

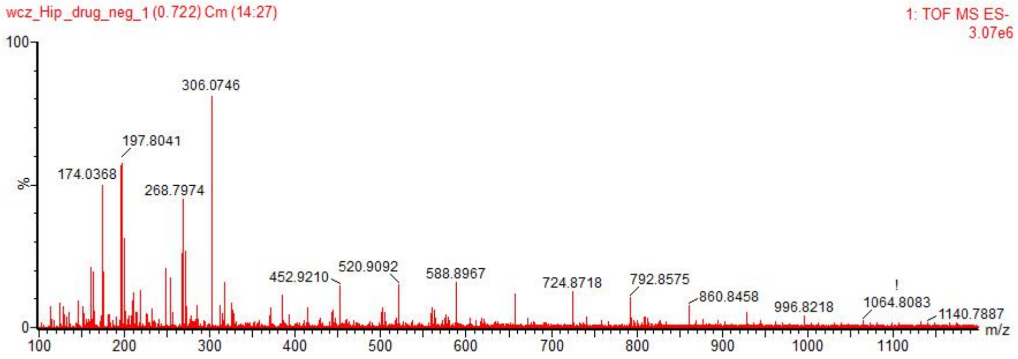

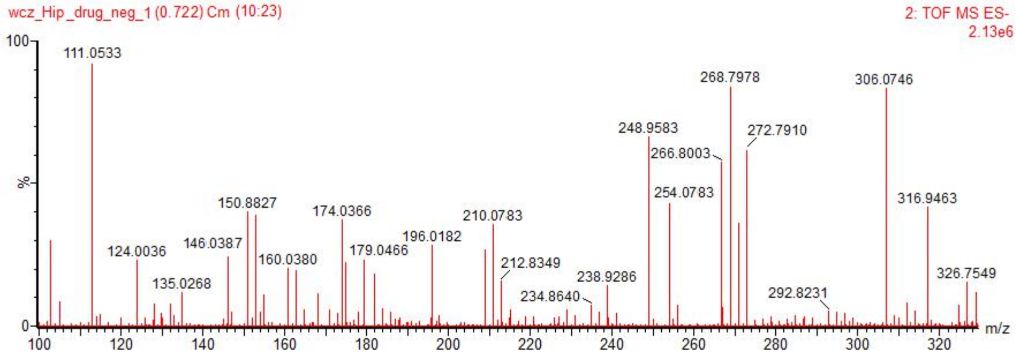

5. MS Spectra of Citric acid in Function 1 and Function 2

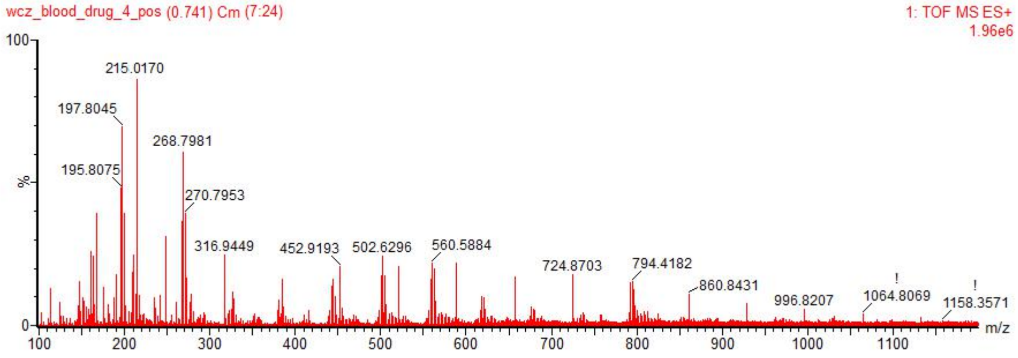

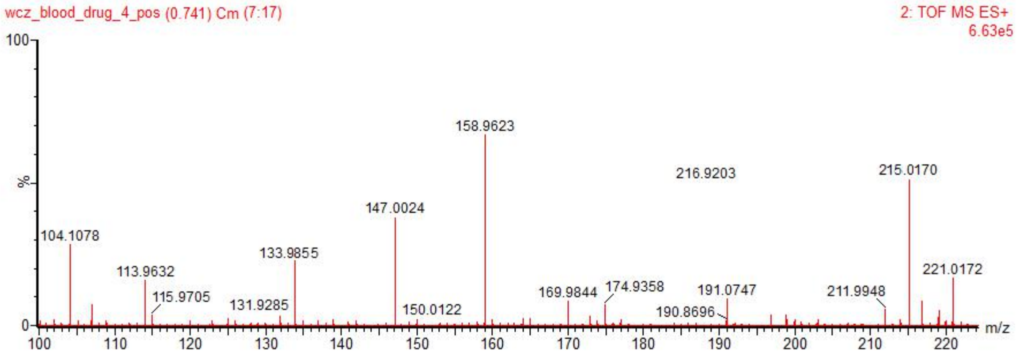

6. MS Spectra of Oxidized glutathione in Function 1 and Function 2

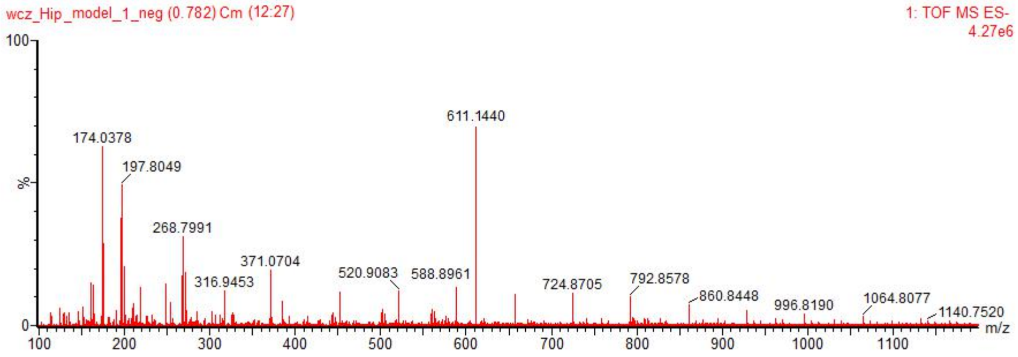

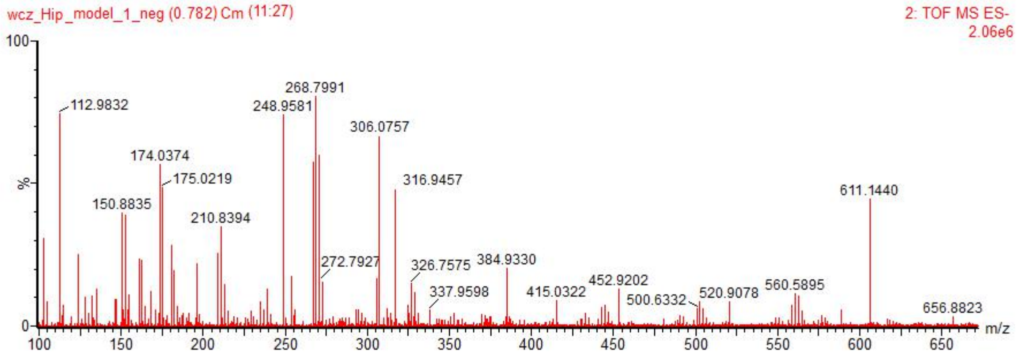

7. MS Spectra of Pantetheine 4'-phosphate in Function 1 and Function 2

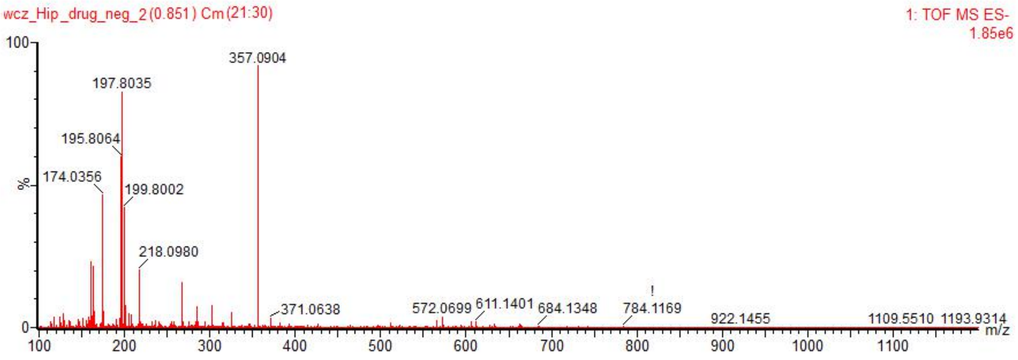

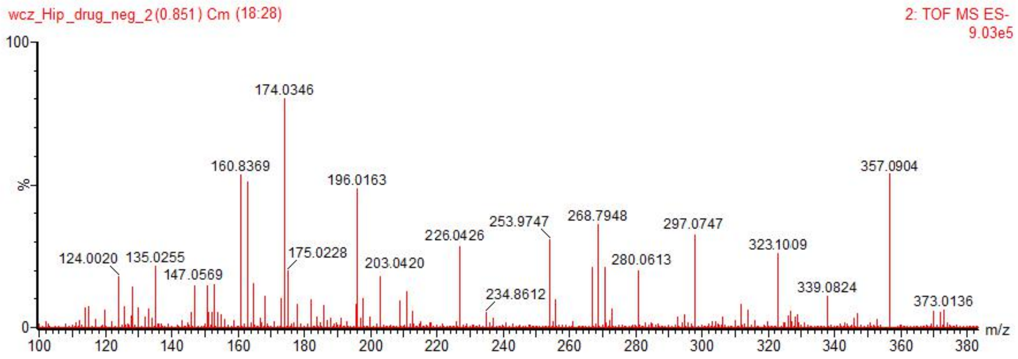

8. MS Spectra of Pantothenic acid in Function 1 and Function 2

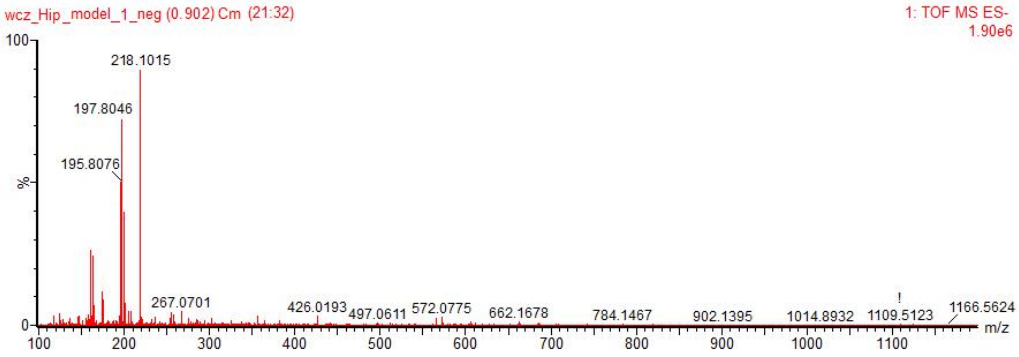

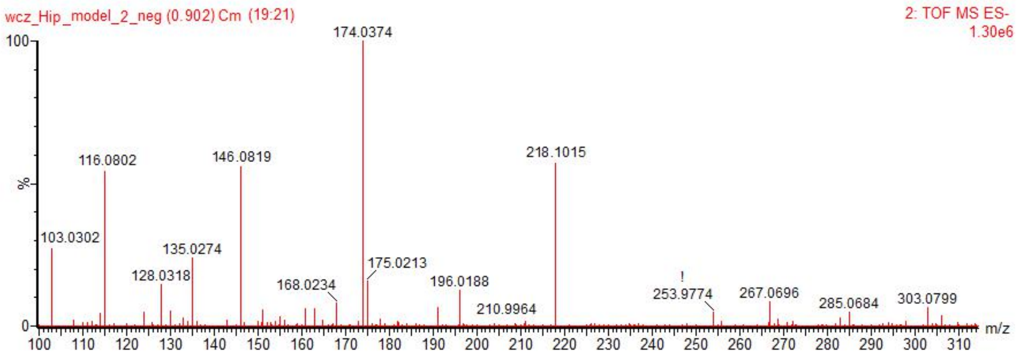

9. MS Spectra of L-Tryptophan in Function 1 and Function 2

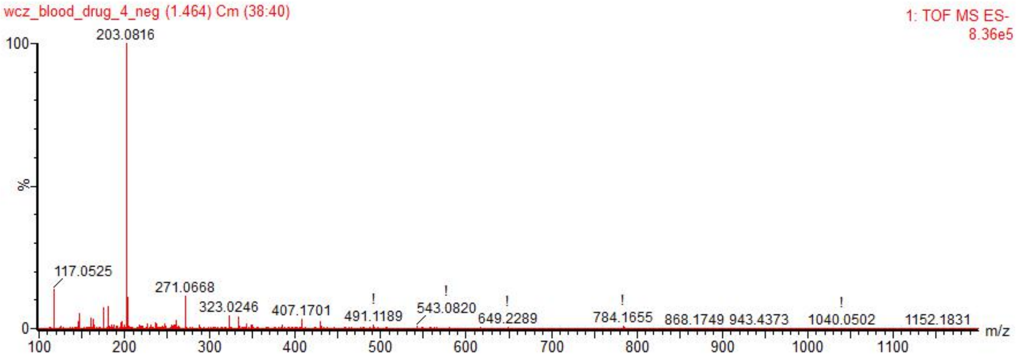

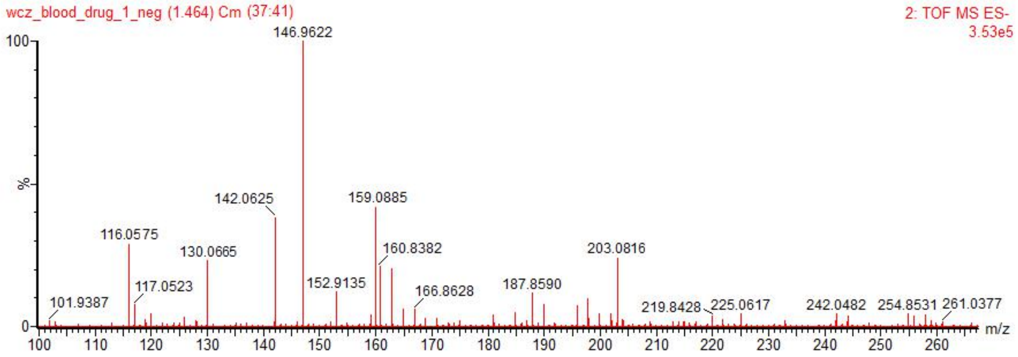

10. MS Spectra of Indoleacetaldehyde in Function 1 and Function 2

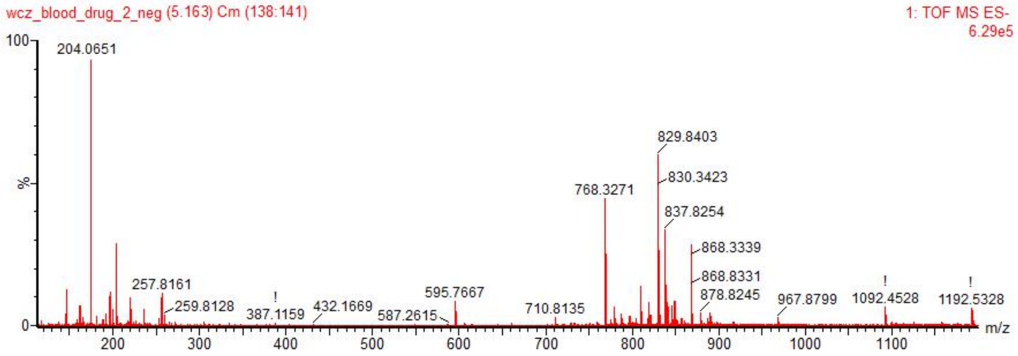

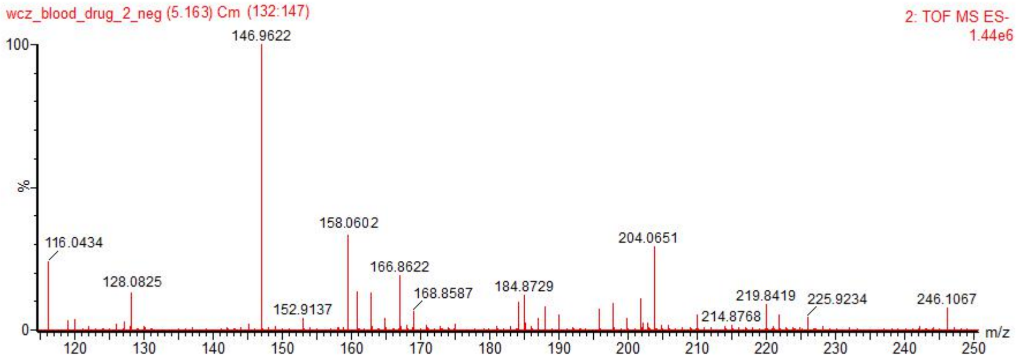

11. MS Spectra of Phytosphingosine in Function 1 and Function 2

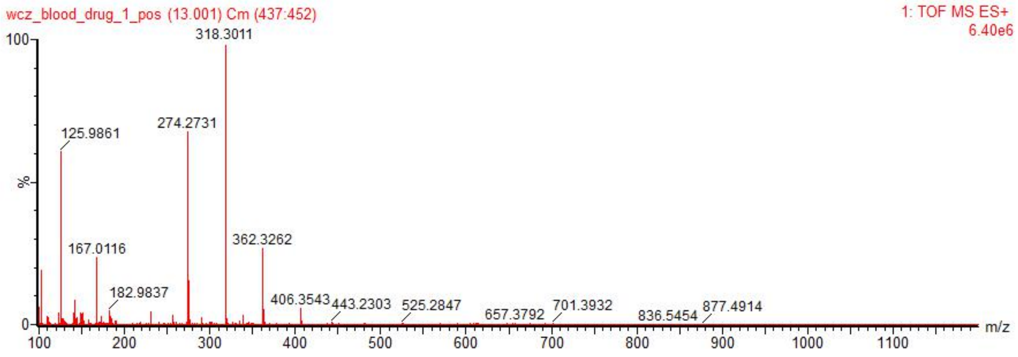

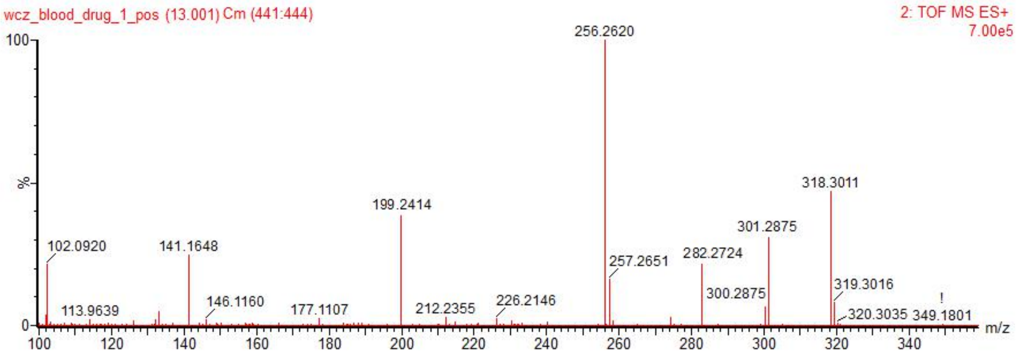

12. MS Spectra of Sphinganine in Function 1 and Function 2

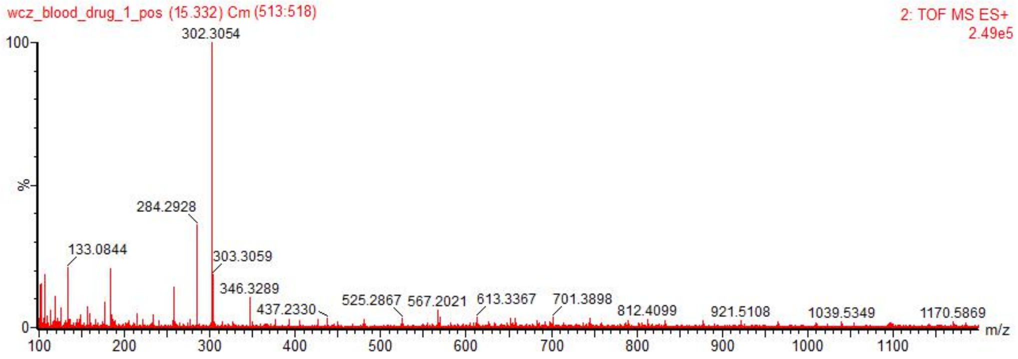

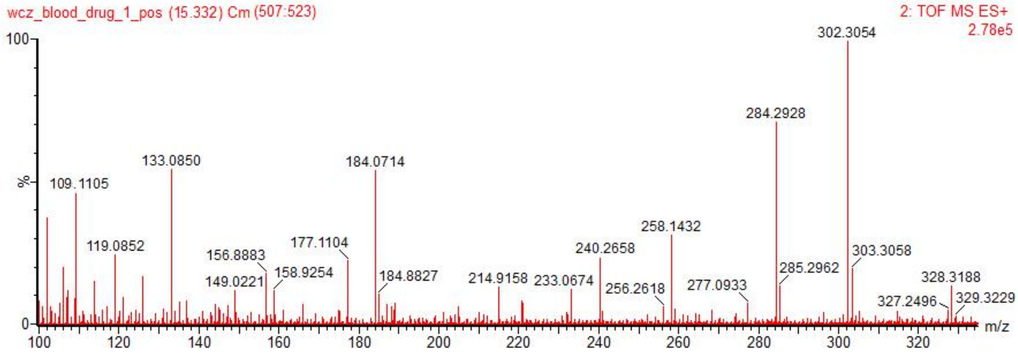

13. MS Spectra of Sphingosine 1-phosphate in Function 1 and Function 2

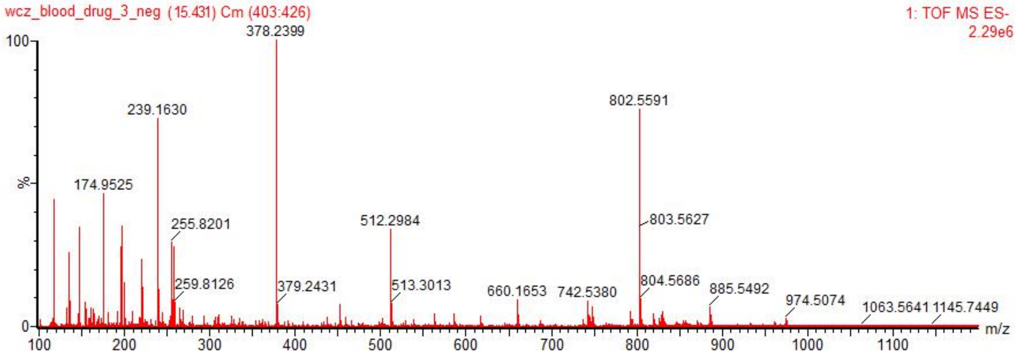

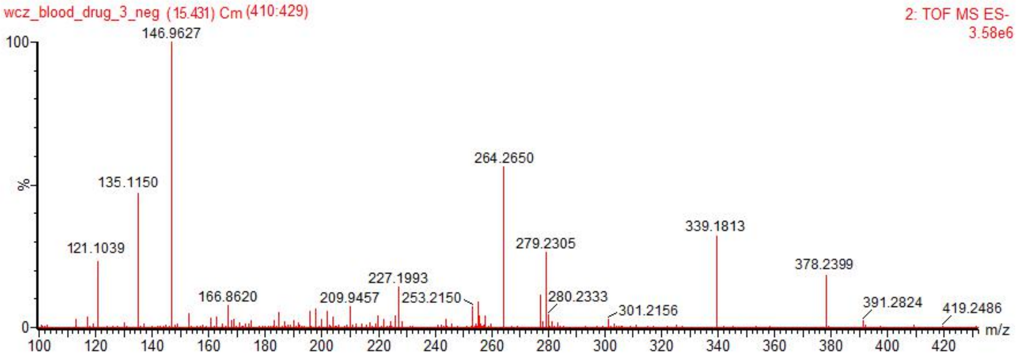

14. MS Spectra of Sphinganine 1-phosphate in Function 1 and Function 2

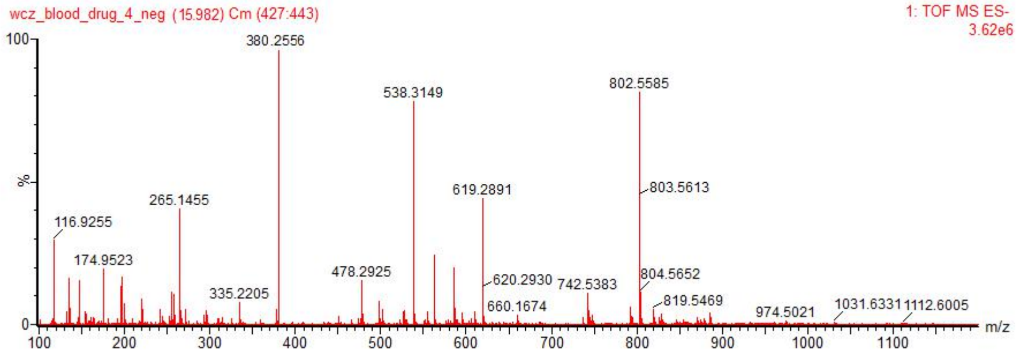

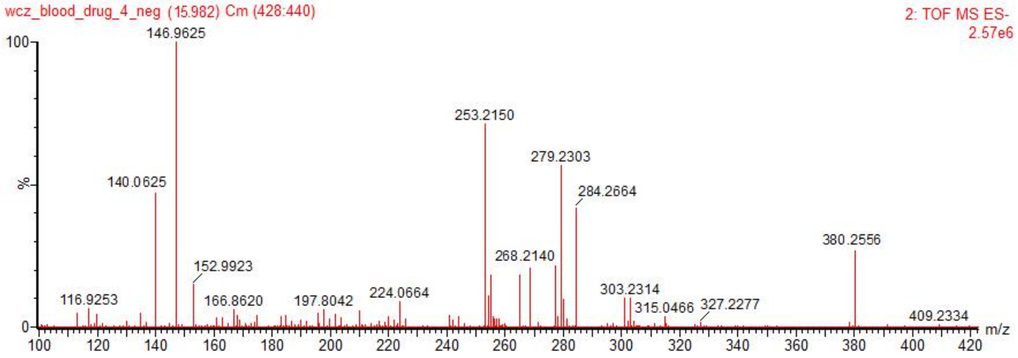

15. MS Spectra of Leukotriene A4 in Function 1 and Function 2

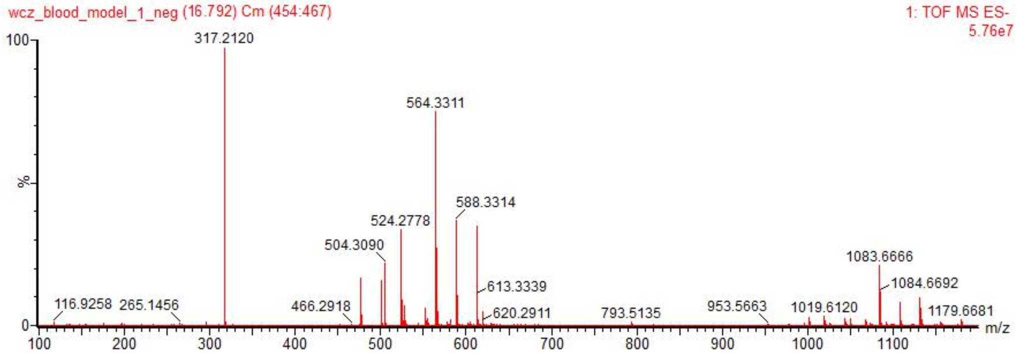

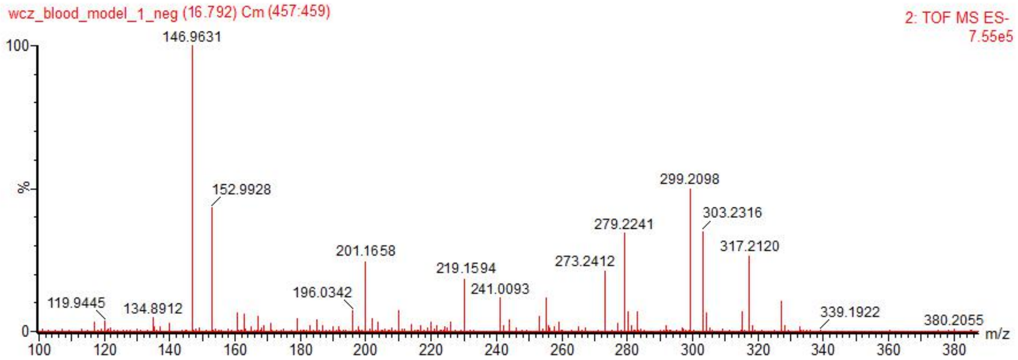

16. MS Spectra of 12,13-EpOME in Function 1 and Function 2

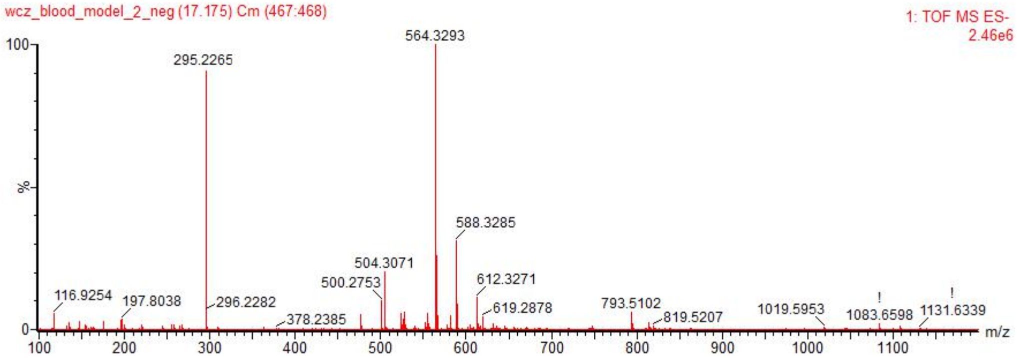

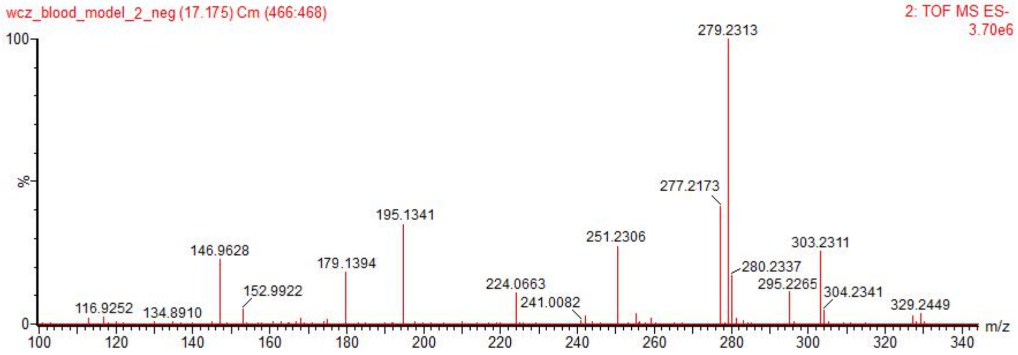

17. MS Spectra of 19 (S)-HETE in Function 1 and Function 2

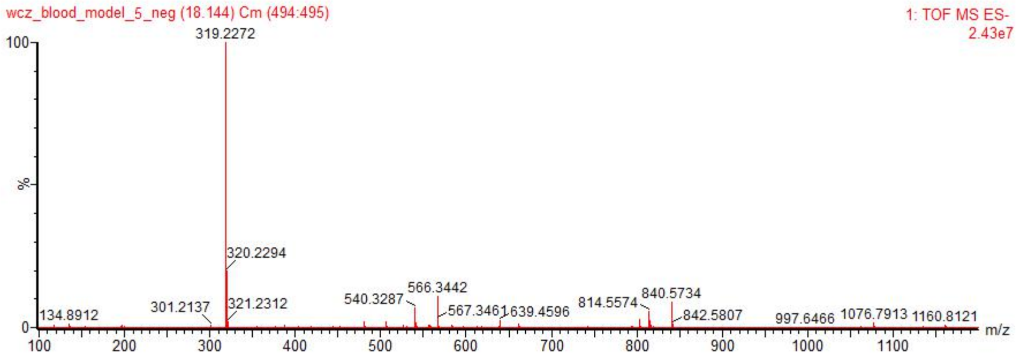

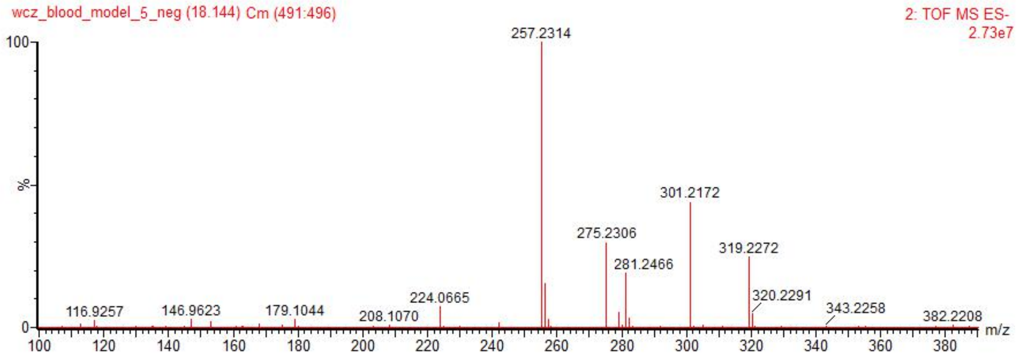

18. MS Spectra of LysoPC (18:1(9Z)) in Function 1 and Function 2

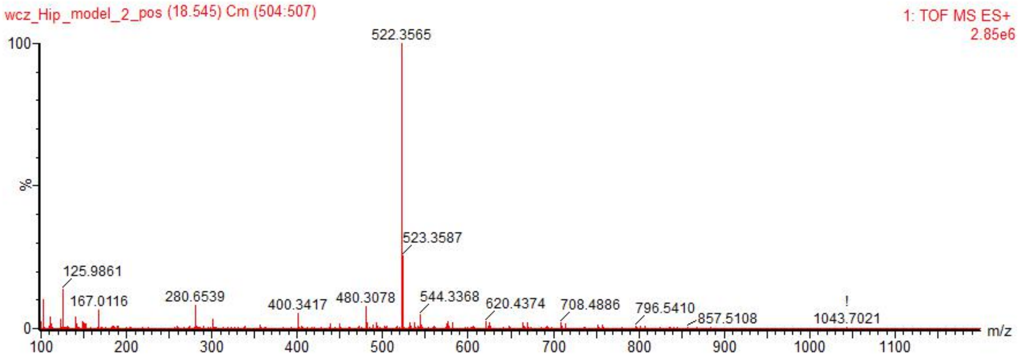

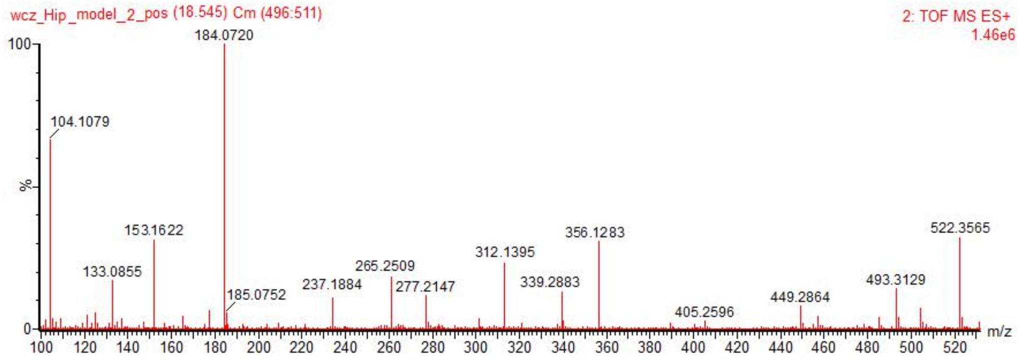

19. MS Spectra of 18:1/18:3 phosphatidylcholine in Function 1 and Function 2

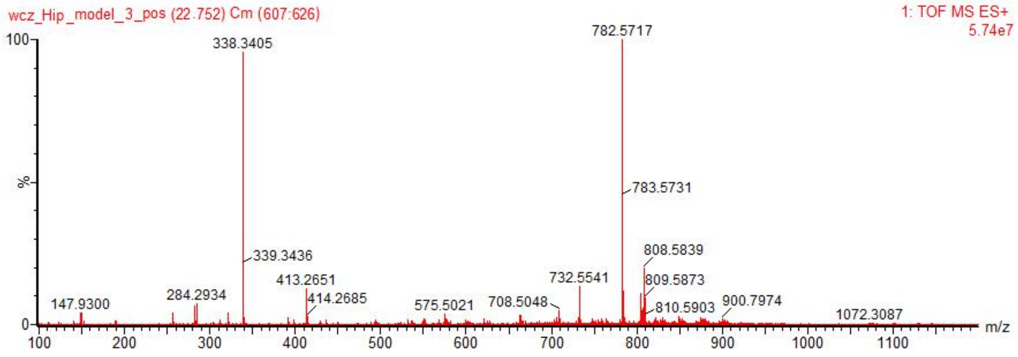

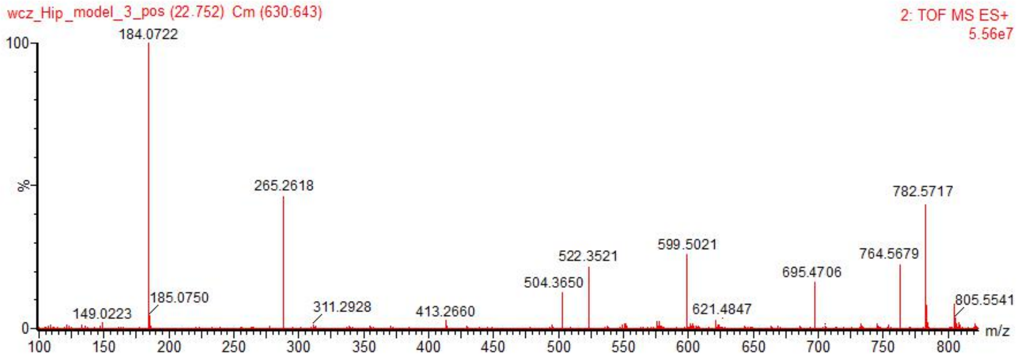

20. MS Spectra of Arachidonic acid in Function 1 and Function 2

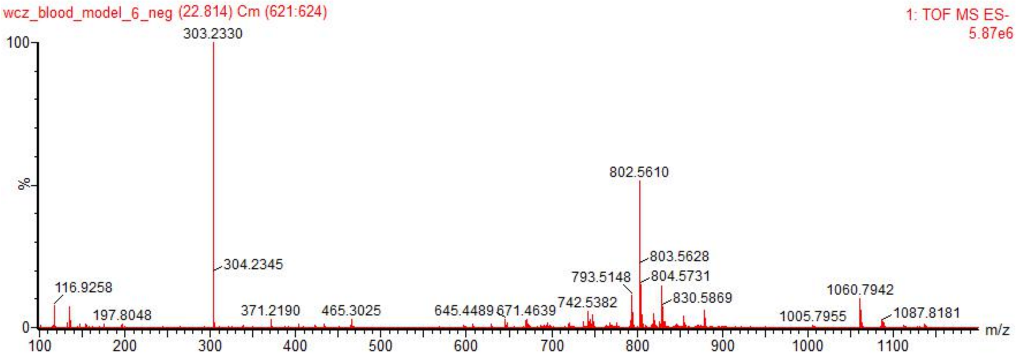

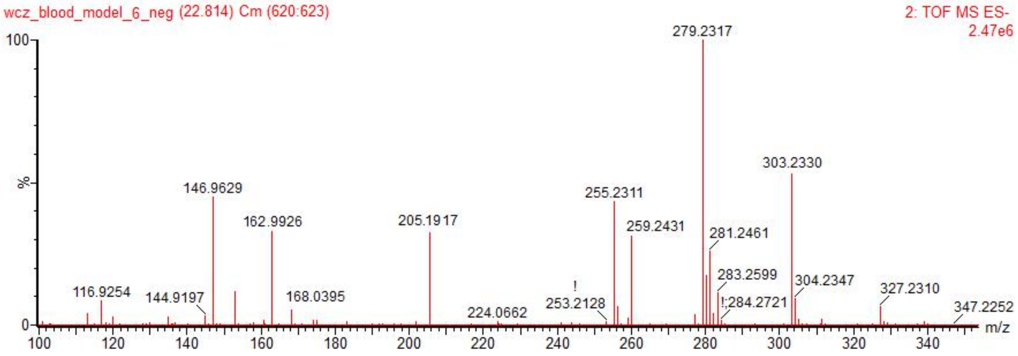

21. MS Spectra of Linoleic acid in Function 1 and Function 2

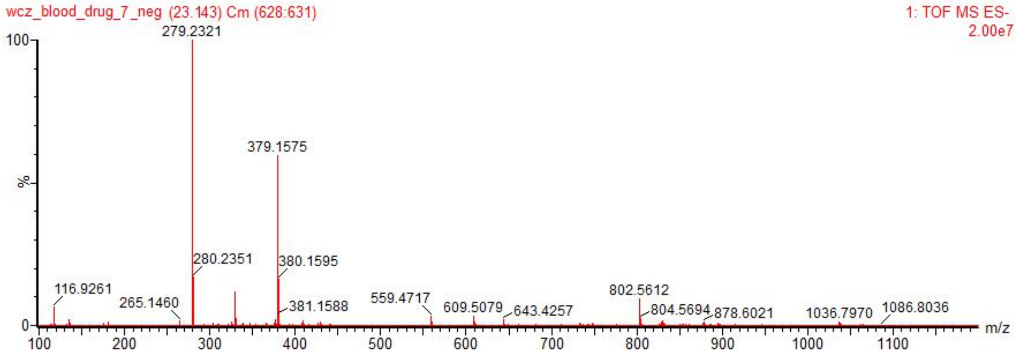

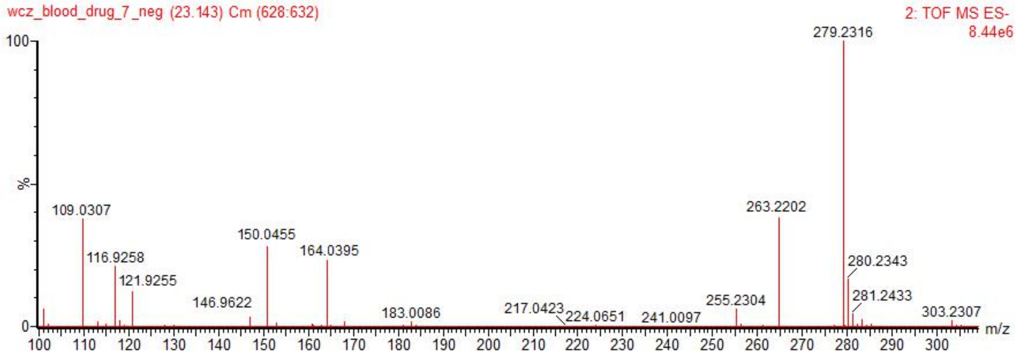

22. MS Spectra of 20-Hydroxy-leukotriene B4 in Function 1 and Function 2

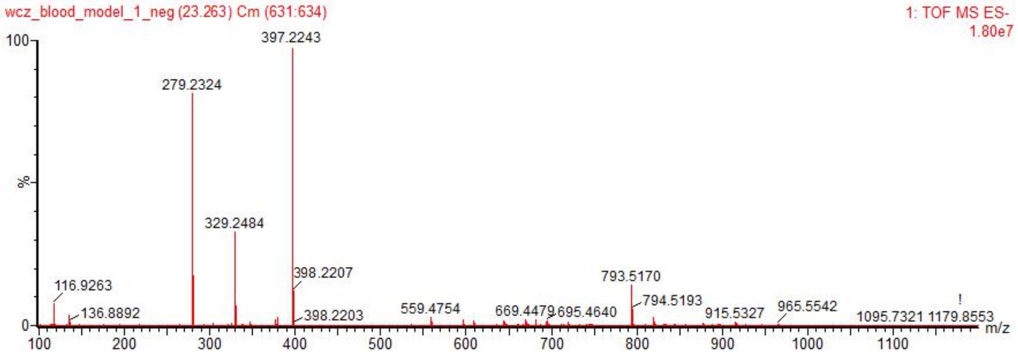

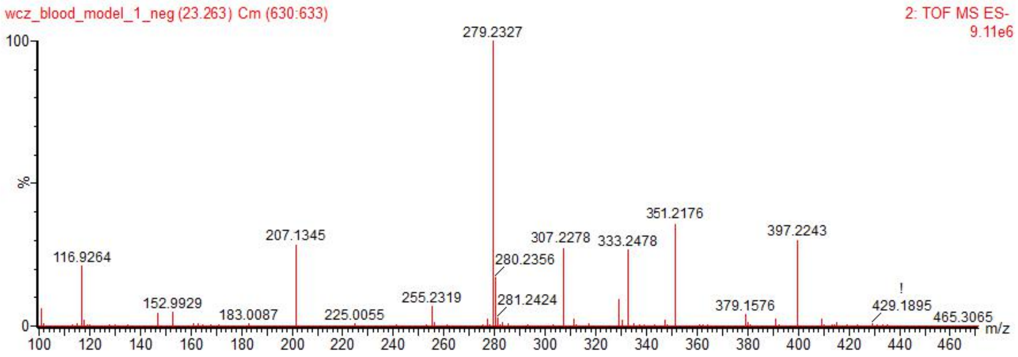

23. MS Spectra of 18:1/18:2 phosphatidylcholine in Function 1 and Function 2

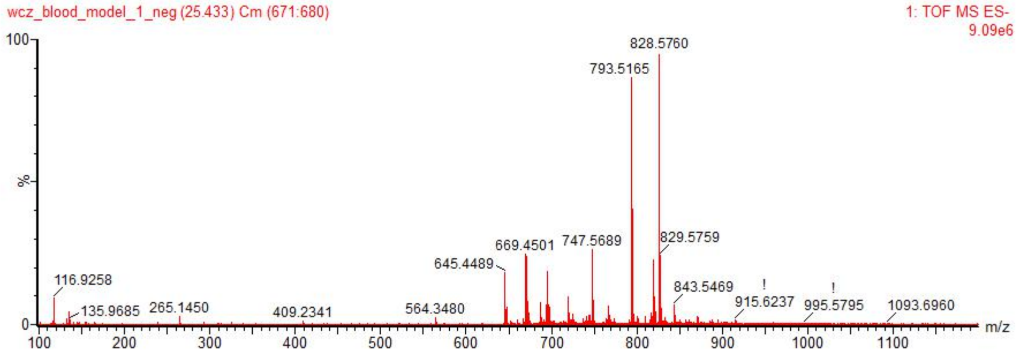

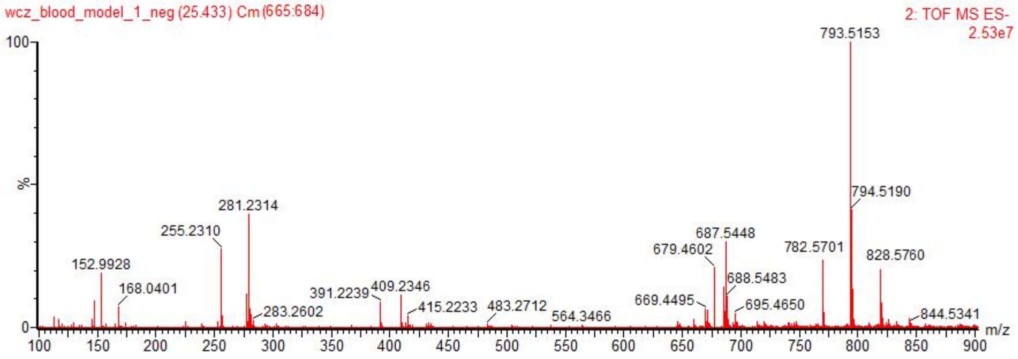

24. MS Spectra of Pantetheine 4'-phosphate in Function 1 and Function 2

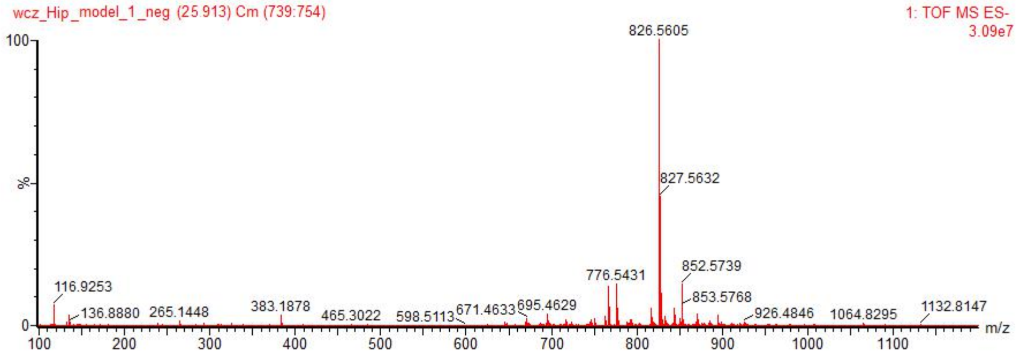

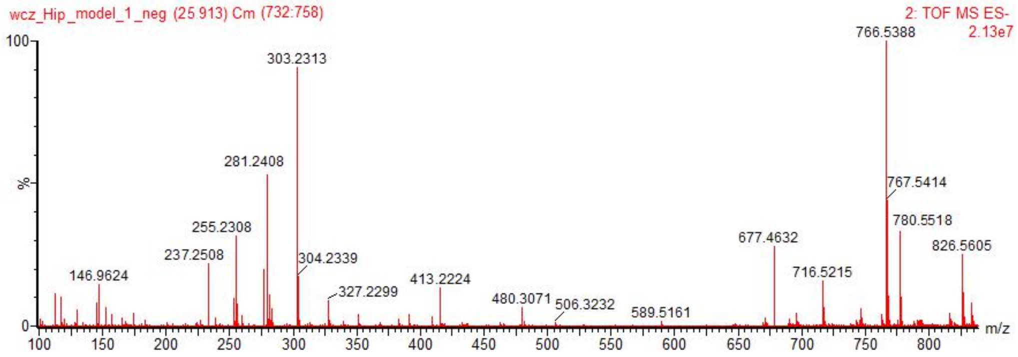

25. MS Spectra of Lactosylceramide in Function 1 and Function 2

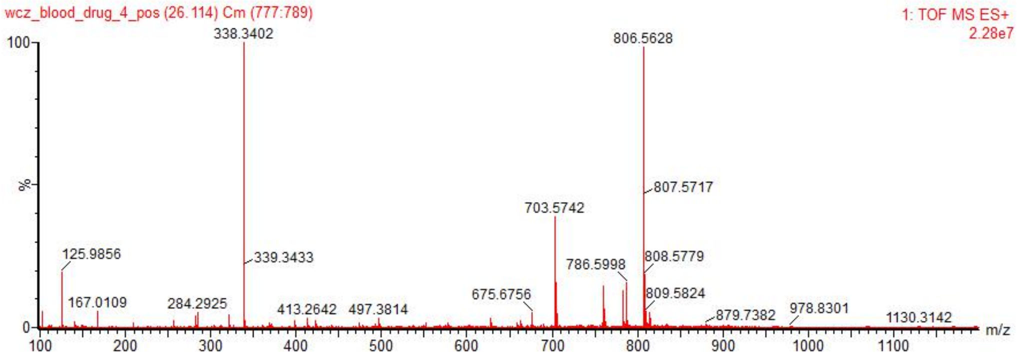

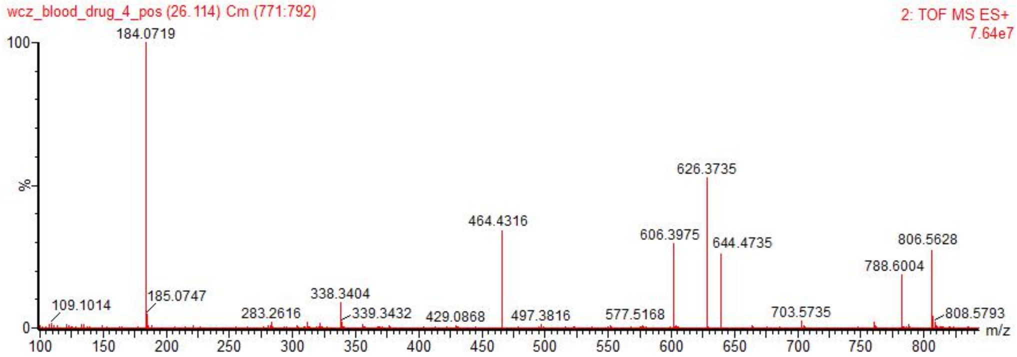

26. MS Spectra of 18:2/18:3 phosphatidylcholine in Function 1 and Function 2

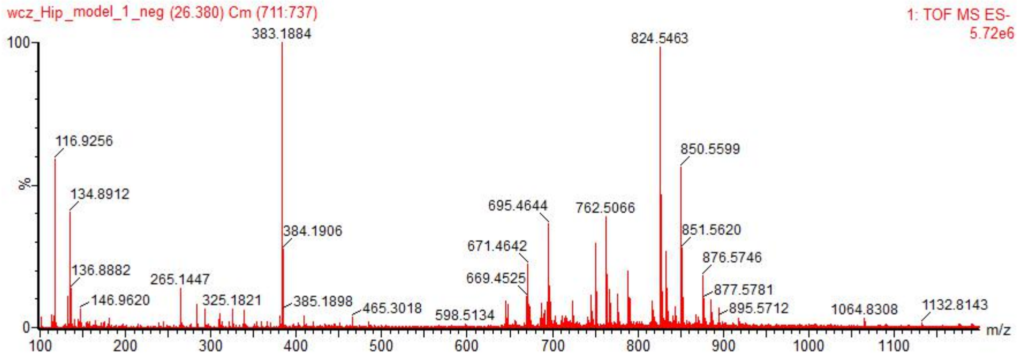

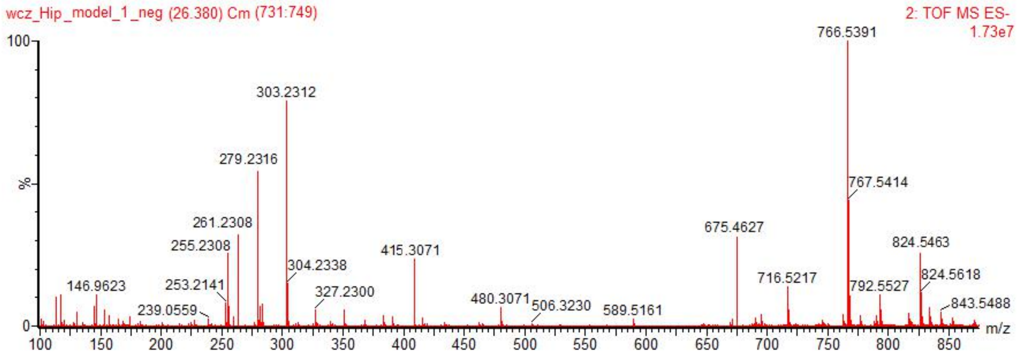

27. MS Spectra of SM (d18:0/16:1) in Function 1 and Function 2

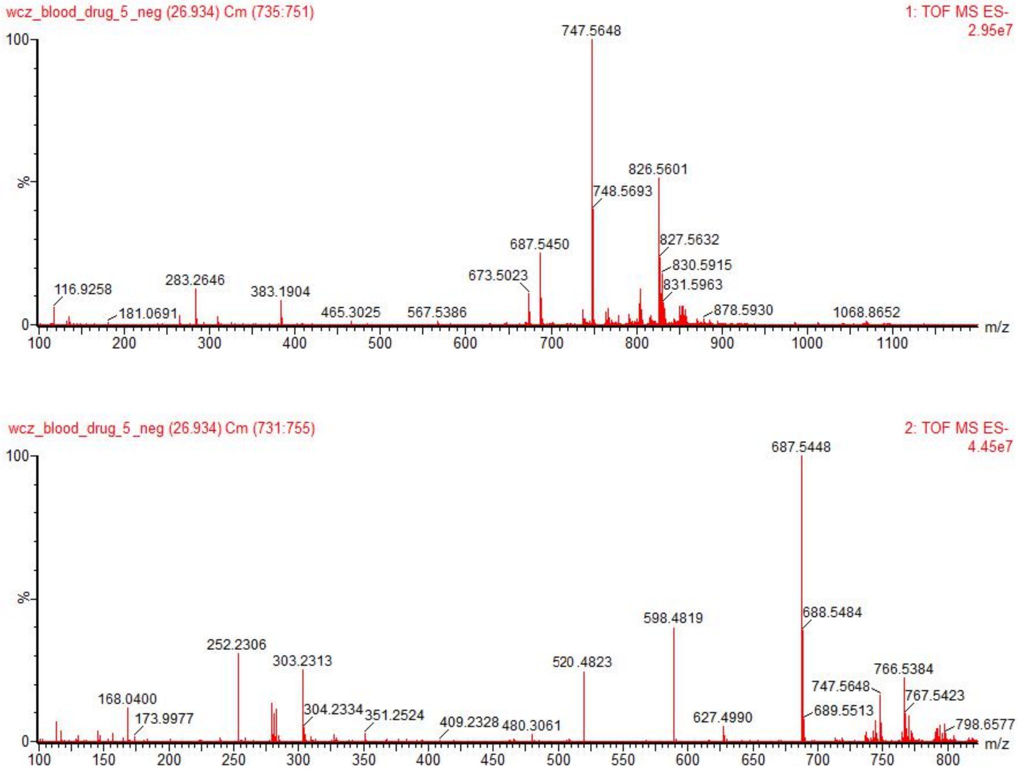

28. MS Spectra of 16:0/18:1 phosphatidylcholine in Function 1 and Function 2

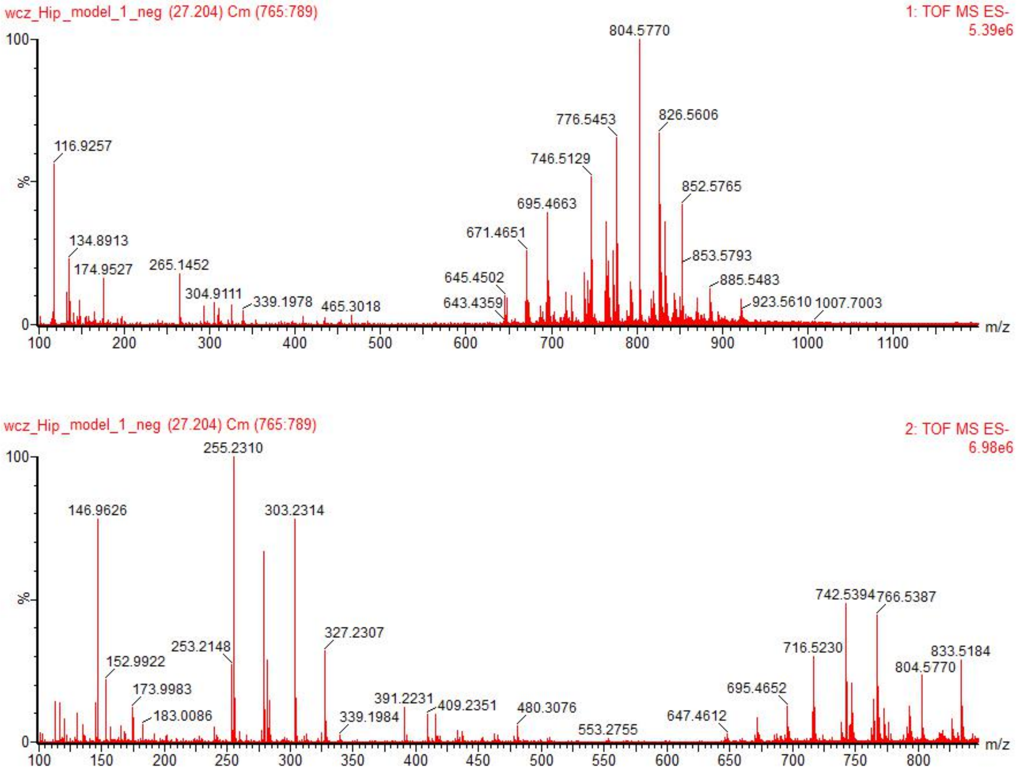

29. MS Spectra of 18:2/18:1 phosphatidylcholine in Function 1 and Function 2

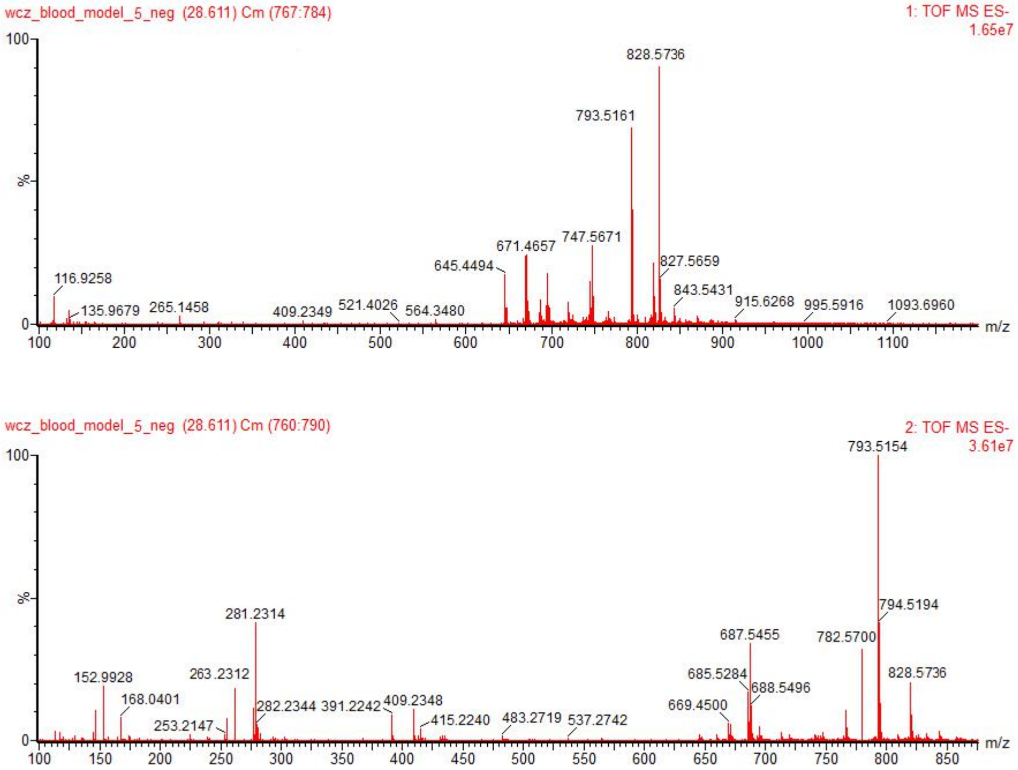

Supplement: Supplementary file 1 [file molecules-24-01712-s001.pdf]
